# Genome-Wide Association Mapping for Yield and Other Agronomic Traits in an Elite Breeding Population of Tropical Rice (*Oryza sativa*)

**DOI:** 10.1371/journal.pone.0119873

**Published:** 2015-03-18

**Authors:** Hasina Begum, Jennifer E. Spindel, Antonio Lalusin, Teresita Borromeo, Glenn Gregorio, Jose Hernandez, Parminder Virk, Bertrand Collard, Susan R. McCouch

**Affiliations:** 1 International Rice Research Institute, Los Baños, Philippines; 2 Department of Plant Breeding and Genetics, Cornell University, Ithaca, NY, United States of America; 3 Crop Science Cluster, University of the Philippines Los Baños, Los Baños, Philippines; 4 International Center for Tropical Agriculture, Cali, Colombia; Pennsylvania State University, UNITED STATES

## Abstract

Genome-wide association mapping studies (GWAS) are frequently used to detect QTL in diverse collections of crop germplasm, based on historic recombination events and linkage disequilibrium across the genome. Generally, diversity panels genotyped with high density SNP panels are utilized in order to assay a wide range of alleles and haplotypes and to monitor recombination breakpoints across the genome. By contrast, GWAS have not generally been performed in breeding populations. In this study we performed association mapping for 19 agronomic traits including yield and yield components in a breeding population of elite irrigated tropical rice breeding lines so that the results would be more directly applicable to breeding than those from a diversity panel. The population was genotyped with 71,710 SNPs using genotyping-by-sequencing (GBS), and GWAS performed with the explicit goal of expediting selection in the breeding program. Using this breeding panel we identified 52 QTL for 11 agronomic traits, including large effect QTLs for flowering time and grain length/grain width/grain-length-breadth ratio. We also identified haplotypes that can be used to select plants in our population for short stature (plant height), early flowering time, and high yield, and thus demonstrate the utility of association mapping in breeding populations for informing breeding decisions. We conclude by exploring how the newly identified significant SNPs and insights into the genetic architecture of these quantitative traits can be leveraged to build genomic-assisted selection models.

## Introduction

Developing new rice varieties that yield well with fewer inputs and under more stressful and unpredictable climatic conditions is essential for the future of food security, and is the major challenge for today's rice breeders [1,2]. Fortunately, the rapid development of new sequencing technologies has created the opportunity to enhance our understanding of the genetic basis of crop productivity. The utilization of this genetic information offers the plant breeding community a range of modern tools and methods for addressing these challenges [3].

Genome wide association studies (GWAS) have been widely used to identify QTL underlying quantitative traits in humans and animals, and has recently also become a popular method of mapping QTL in plants. Association mapping identifies QTL based on the historic recombination in a panel of diverse germplasm via the presence of linkage disequilibrium (LD) between SNPs and QTL, i.e., the non-random association of alleles [4,5,6]. A high density marker panel that covers the genome is required in order to monitor the density of recombination breakpoints in the population [6,7].

GWAS are most commonly performed in diversity panels, i.e., collections of unrelated diverse germplasm, in order to maximize the diversity of alleles and haplotypes [8,9,10,11,12,13]. While this is advantageous in terms of identifying novel QTL and candidate genes that underlie agronomic traits of interest, it also requires that any identified QTL be validated in a breeding population before they can be used for genomics-assisted selection. For this reason, it is of interest to perform GWAS in a population of adapted lines. QTL identified in this way could be more directly utilized for marker assisted selection (MAS) and/or genomic selection in applied breeding programs [14].

Genome-wide prediction, or genomic selection (GS), refers to the process of using genome-wide DNA markers to predict which individuals in a breeding population are most valuable as parents of the next generation of offspring. GS takes the same inputs as GWAS, a phenotype dataset and genotype dataset on a population of lines of interest to plant breeders [14,15,16]. As such, it is possible to perform GWAS and GS on the same population, where all that is needed is additional computation analysis. Such an undertaking has clear advantages. The genetic architecture revealed by association mapping can be used to inform the GS models—for example, if highly significant SNPs are revealed by a GWAS, these SNPs could be fit as fixed effects in a GS model [14,17], and experimenting with different types of genomic selection statistical methods (i.e., linear versus non-linear, additive versus non-additive) can corroborate inferences about the genetic architecture of a trait.

We performed both GWAS and genomic selection on a population of elite breeding lines from the International Rice Research Institute (IRRI) irrigated rice breeding program in order to map QTL for agronomic, morphological and yield-related traits. In this paper, we report the association mapping results, which are leveraged for MAS and used to inform GS modeling on the same population.

## Results and Discussion

### Genotyping and population structure analysis

Genotyping-by-sequencing (GBS) was used to discover and genotype SNPs on 369 advanced breeding lines from the IRRI irrigated rice breeding program. Raw GBS data were imputed and the resulting matrix of SNP calls filtered on call rate and minor allele frequency (MAF) to obtain a set of 73,147 SNPs with call rates > = 90% (no MAF filter) and a set of 71,710 SNPs with call rates > = 75% and MAF > = 0.05. Six individuals with missing data > = 60% were removed from both SNP sets for a total of 363 genotyped lines. Data were transformed to numeric values and the minimal remaining missing data filled using the genotypic means of the lines (S4 Fig., materials and methods).

The majority of the 363 lines were classified *a priori* based on pedigree records as belonging to the *indica* or *indica-admixed* subpopulation groups. To confirm these classifications, assess population structure, and identify outlier individuals, principal component analysis (PCA) was performed using the 73,147 SNP dataset. In rice diversity panels, the first four principle components correspond to the five rice subpopulations. The first principle component separates *indica*/*aus* individuals from *japonica*/*aromatic* individuals, the second principle component separates *aus* and *indica* individuals, the third principle component separates *temperate japonica* individuals from *tropical japonica* individuals, and the fourth principle component separates the *aromatic* individuals from the *japonica* individuals [11].

In our breeding panel, we did not expect all five rice subpopulations to be represented; this assumption was confirmed by our PCA results. The first principle component explained ∼26.3% of the variance, after which the proportion of variance explained by the remaining principle components dropped off sharply (∼2.7% for PC 2, 2.2% for PC3, and 1.9% for PC4), indicating that only 2 out of the 5 subpopulations were present: *indica* and *tropical japonica*. Based on a plot of the first two principle components, 13 individuals were identified as belonging to the *japonica* subpopulation and excluded from subsequent analyses (S1 Fig.), in theory, leaving only *indica* individuals in the dataset. After removing these 13 *japonica* individuals, a second PCA was performed to evaluate remaining subpopulation structure. In this second PCA, the first principle component explained only 5.4% of the variance, indicating that the majority of the remaining individuals belonged, in fact, to a single population. Based on the scatterplot matrix of the first four principle components, however, we removed an additional 18 individuals that showed evidence of admixture (S1 Fig.). After removing these additional lines, a third PCA suggested that no significant subpopulation structure remained in the dataset. Thus, the final set of individuals used for the GWAS contained 332 *indica* individuals.

As a final check that we had adequately controlled for population structure in the dataset, we ran the GWAS both with and without the first principal component fit as a model covariate and confirmed that it did not have an effect on the results. The QQ plots for the final model indicate that we effectively control for subpopulation structure (S2 Fig.) (materials and methods).

### GWAS for identification of QTL

**Fig 1:**
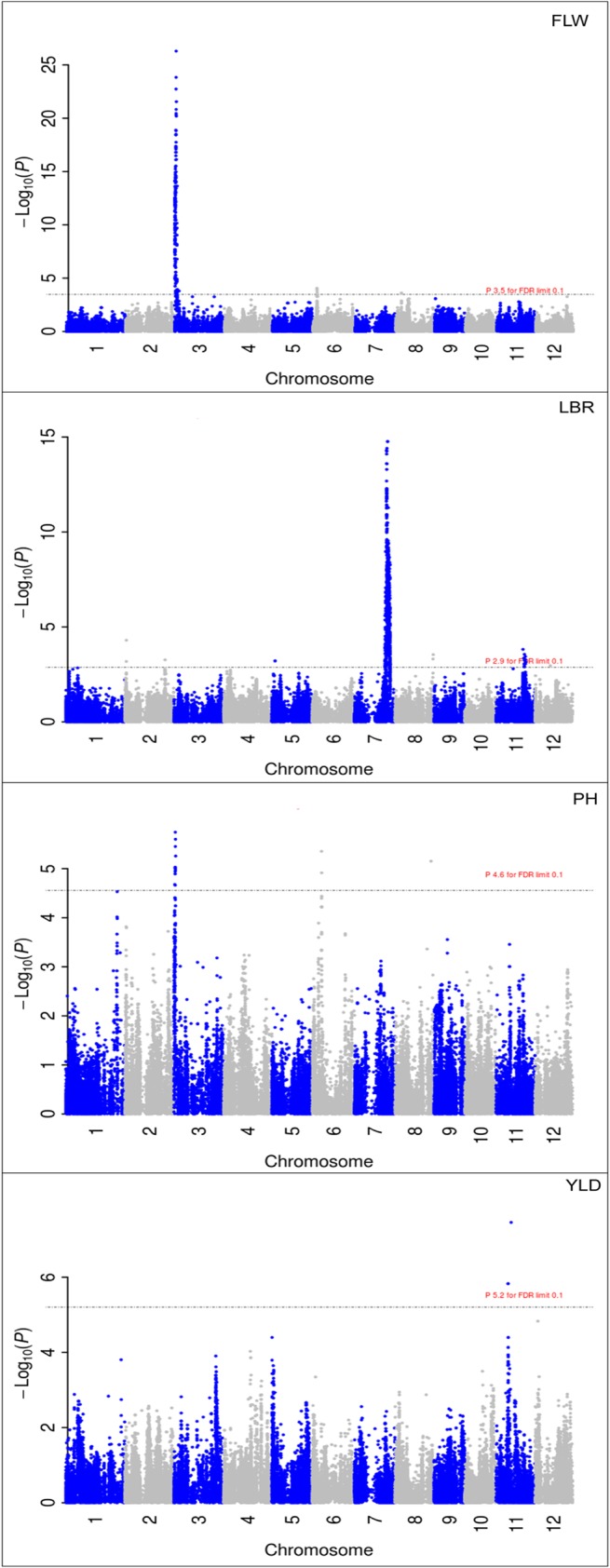
Selected Manhattan plots for flowering time (FLW, top), length-breadth ratio (LBR, top middle), plant height (PH, bottom middle), and grain yield (YLD, bottom). Dashed line shows the 0.1 FDR significance threshold.

**Fig 2:**
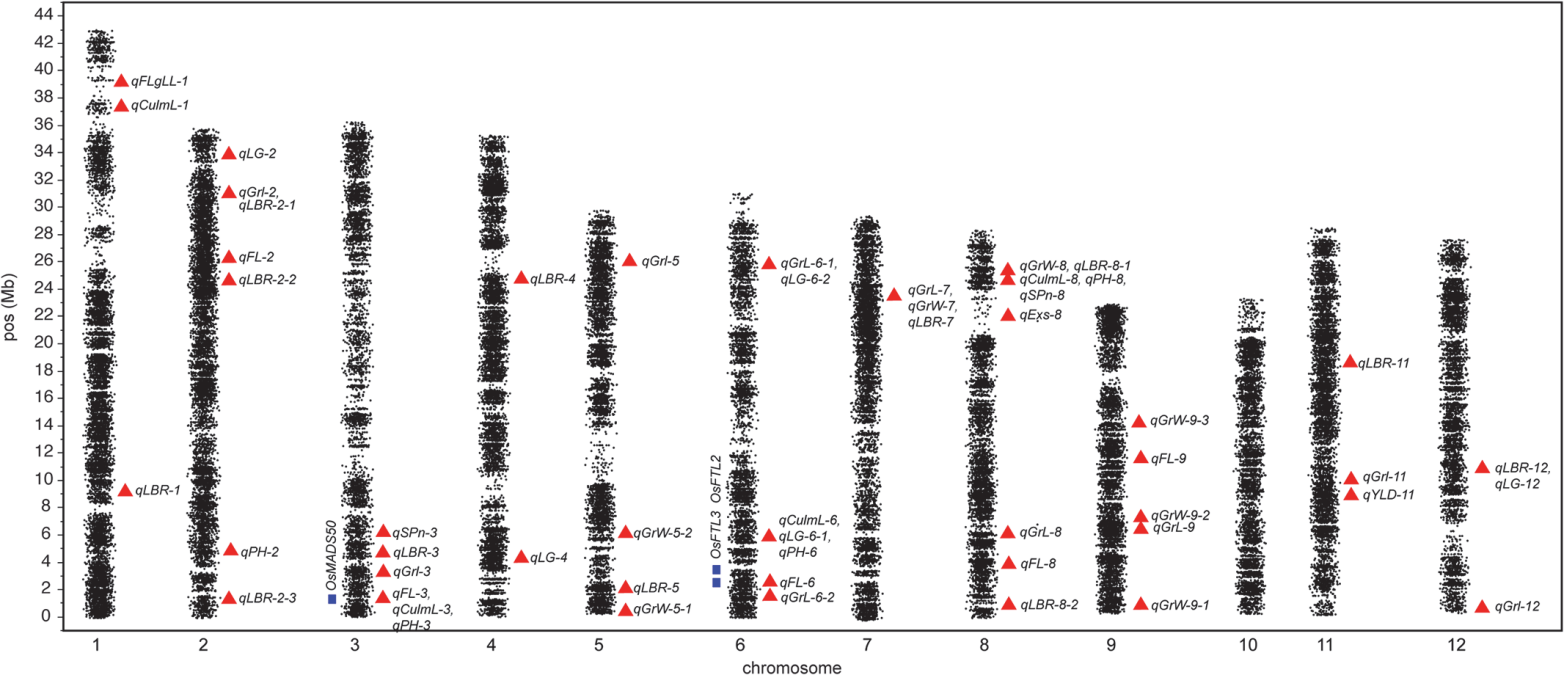
Physical map of significant GWAS QTL. Black points—jittered GBS SNPs, red triangles—physical position of the most significant SNP for a given peak, blue rectangles—physical position of candidate flowering time genes.

A total of 52 QTL were identified for 11 of the 19 agronomic traits evaluated in this study (Table 1). Peaks for the other eight traits did not pass the significance threshold as determined by a false discovery rate (FDR) of 0.1; all traits were evaluated during both the 2012 dry and wet seasons in Los Baños, Philippines (Figs. 1–2, S1 Table, S3 Fig.) (materials and methods). The Manhattan plots for flowering time (FLW), plant height (PH), and length-breadth ratio (LBR) in the dry season and yield (YLD) in the wet season are shown in Fig. 1. Manhattan plots for the rest of the traits are given in S3 Fig.

**Table 1:** Description of phenotypes and phenotyping.

**Trait**	**Description of phenotyping**	**Trait Ontology (TO)**	**TO synonyms**	**Plant Ontology (PO)**
Plant height (PH)	actual measurements in cm from soil surface to tip of tallest panicle (awns excluded)	TO:0000207	Ht, PTHT, shoot height	shoot axis PO 0025029
Flowering date (FLW)	When 50% of flowers were visible in the whole plot	TO:0000344	days to flowering, Delay in flowering time, DTFL	1/2 of flowers open stage PO:0007053
Culm length (CulmL)	Measure from soil surface to panicle base in cm	TO:0000576	CmL, core length, culm height, culm length, CULMLG, stem height, STEMLG	stem PO:0009047
Number of effective tiller or panicle per plant (PN)	Count of number of panicles from each plant	TO:0000152	NOP, NP, number of effective tiller per plant, number of panicle, panicle number per plant, panicle number per tiller, PN, PNNB, seed setting tillers per plant, spike number, TP	inflorescence PO:0009049
Panicle length (PL)	Actual measurements in cm of panicle base to tip of each panicle	TO:0000040	PnL, PNLG	inflorescence PO:0009049
Flag leaf length (FlgLL)	Flag leaf length measured in cm of all flag leaves of each panicle per plant	TO:0002757	FLFLG	flag leaf PO:0020103
Flag leaf width (FlgLW)	Flag leaf width measured in cm of all flag leaves of each panicle per plant, measured at the widest portion of the leaf blade	TO:0000370	LFWD, LW	flag leaf PO:0020103
Flag leaf area (FlgLA)	Calculated as K x L x W., where K is the "adjustment factor". K is dependent on the shape of the leaf which, in turn, is affected by the plant variety, nutritional status, and growth stage of the leaf. Experimental studies at IRRI (IRRI, 1972) have indicated that a value of 0.75 can be used for all stages of growth except at the seedling stage and at harvest, where the value of 0.67 should be used instead	TO:0000540	LFAR	flag leaf PO:0020103
Number of spikelets per panicle (SPn)	the total number of spikelets (whether filled or empty) per plant divided by the panicle number per plant	TO:0000456	number of spikelets per panicle, spikelet number per panicle, SPKNB	inflorescence PO:0009049
Number of filled grain per plant (FGP)	manual count of the filled grain per plant	TO: 0000447	FGRNB, FILGRNB, filled fruit number, filled grain number per panicle, grain number per panicle, GRNBPPN, number of grains per panicle	fruit PO:0009001
Grain length (GrL)	Random selection of ten seeds, individually measured for grain length in mm using an electronic digital caliper with a precision of 0.1 mm	TO:0002626	grain length, GRLG, pod length	fruit PO:0009001
Grain width (GrW)	Actual measurement of width in mm as the distance across the fertile lemma and palea at the widest point on the same grains measured for length	TO:0000402	dehulled grain curved width, dehulled-grain width, DHULGRWD, GRWD, width of grain without hull	fruit PO:0009001
Grain length-breadth ration (LBR)	grain length divided by grain width	TO:0002731	dehulled-grain length to width ratio, DHULGRLGWDRO, GRLHWDRO	fruit PO:0009001
Lodging score (LG)	percent of plants that lodged	TO:0000068	Lg, LOI	
Peduncle length (PedL)	The upper most internode length of the panicle or peduncle length measured in cm as the length of peduncle from the base of the flag leaf to the base of basal spikelet of a spike	TO:0002691	neck internode length	peduncle PO:0009053
Panicle exertion rate (Exs)	The ratio of the total panicle length that comes out from flag leaf sheath to the total panicle length. Scored as 1 for fully exerted, 3 for moderately exerted, 5 for recently exerted, 7 for partially exerted and 9 for enclosed	TO:0000165	Exs, PNEX	inflorescence PO:0009049
1000 grain weight (1000GW)	Measurements in g of 1000 well developed whole grains, dried to 13% moisture content, weighed on a precision balance	TO:0000382	1000-grain weight, TGRWT, thousand grain weight, thousand seed weight, TO:0000533, TSDWT	fruit PO:0009001
Yield per plant (YPP)	Measurements in g of total grain for one plant (randomly selected five plants from one plot) dried to 13%MC, weighed on a precision balance	TO:0000449	GRYLDPPL	fruit PO:0009001
Grain yield per plot (YLD)	Yield in kg per hectare at 14% moisture, calculated from the harvest of a 5 meter square per plot (boarder rows discarded)	TO:0000396	GRYLD, Yld	fruit PO:0009001

The genetic effects of the majority of QTL identified were relatively small (<20% phenotypic variance explained, PVE). The four QTL identified for culm length in the dry season, for example, explained a total of ∼59% of the phenotypic variance. Collectively, the 10 QTL identified for grain length in the dry season explained only ∼31% of the phenotypic variance, the seven QTL for grain width in the dry season explained ∼28% of the variance, and the five QTL for lodging explained 26% of the variance in the wet season but only 13% in the dry season (S1–S2 Tables).

Large effect QTL were, however, identified for some traits. A very large effect QTL for flowering time was identified on chromosome 3 that explained ∼43% of the variance for flowering time during the dry season and 45% during the wet season, and had the effect of decreasing flowering time by an average of six days. Another large effect QTL was identified for grain length, grain width, and length-breadth ratio on chromosome 7 that explained ∼12%, ∼16%, and ∼28% of the variance for grain length, grain width, and length-breadth ratio in the dry season, respectively, and which had the effect of decreasing grain length by an average of ∼0.3 mm, increasing grain width by an average of ∼0.1 mm, and decreasing the grain length-breadth ratio by an average of ∼0.4 mm (S1 and S3 Tables).

Only four out of the 52 identified QTL were significant for both the dry season and the wet season, three of which were the large effect QTL mentioned above for flowering time, grain length, and length-breadth ratio. The other QTL identified in both seasons was an additional QTL for length-breadth ratio on chromosome 2 (qLBR2-2) (S1 Table). A greater number of QTL were identified in the dry season than the wet season due to the fact that heritability is generally higher in the dry season due to more stable climactic conditions. Other differences in the identified QTL and QTL effect sizes between the two seasons are also explained by the divergent environmental conditions of the two seasons, i.e., the wet season is subject to heavy rains, typhoons, and increased disease/pest pressures, while the dry season has a higher average solar radiation and generally more favorable conditions (under irrigation). Interestingly, the one significant QTL identified for yield was identified in the wet season only, suggesting that it is specific to high yields under wet season conditions (S1 Table).

**Fig 3:**
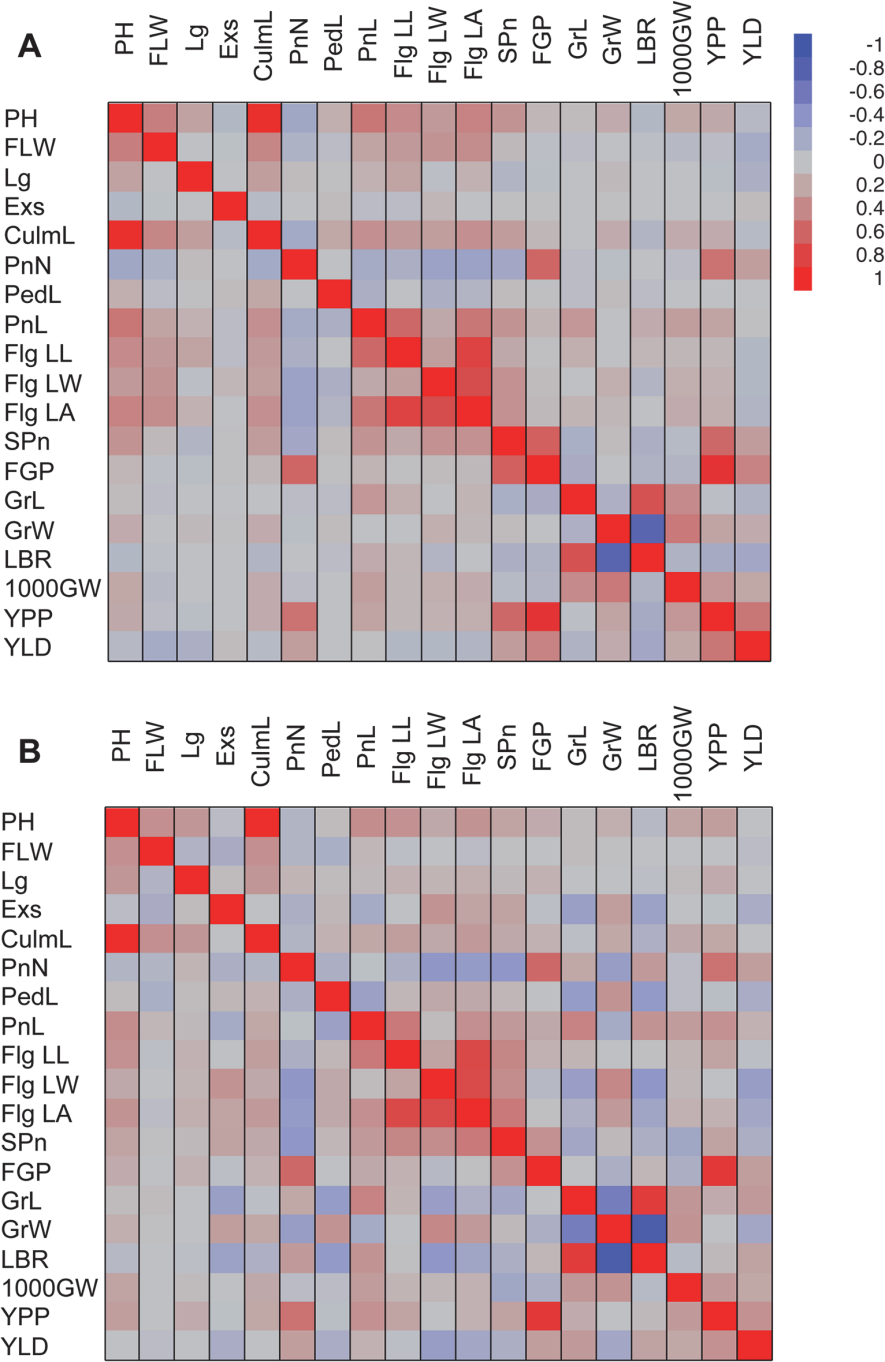
Graphic representation of the correlation matrices of phenotype values for the (A) dry season, and (B) wet season.

Many of the QTL regions were significant for more than one trait. In most of these cases, the traits with co-localized QTL were correlated with each other in one or both seasons (Fig. 3). For example, the grain morphological traits, grain length, width, and length-breadth ratio were correlated with each other (r^2^ = 0.71 for grain length and length-breadth ratio, r^2^ = −0.75 for grain width and length-breadth ratio, and r^2^ = −0.15 for grain width and grain length in the dry season) and shared the major QTL on chromosome 7 mentioned above (*qGrL-7*, *qGrW-7*, *qLBR-7*). Grain length and length-breadth ratio also shared a QTL on chromosome 2 (*qGRL-2*, *qLBR-2-1*), and grain width and length-breadth ratio shared a QTL on chromosome 8 (*qGrW-8*, *qLBR-8-1*). Culm length and plant height were highly correlated in both seasons (r^2^ = 0.95 and 0.98 for the dry and wet seasons, respectively), and shared a QTL on chromosome 3 along with the major flowering time QTL (*qFL-3*, *qCulmL-3*, *qPH-3*), a QTL on chromosome 6 along with a lodging QTL (*qCulmL-6*, *qLG-6-1*, *qPH-6*) and a QTL on chromosome 8 (*qCulML-8*, *qPH-8*) (Figs. 2–3, S1 Table). For such correlated traits with shared QTL, it is likely that either the same causal polymorphism underlies the identified QTL (pleiotropy) or that the genes underlying the QTL are linked.

Some of the QTL for lodging co-localized with QTL for either plant height, grain length, or grain length-breadth ratio. In contrast to the above cases, the correlations of these traits were either absent or weak (r^2^ = 0.24 for plant height and lodging, 0.02 for grain length and lodging, and −0.03 for length-breadth ratio and lodging in the dry season), yet in addition to the QTL described above, a single QTL for grain length and lodging was identified on chromosome 6 (*qGrL-6-1*, *qLG-6-2*) and a QTL for grain length-breadth ratio and lodging was identified on chromosome 12 (*qLBR-12*, *qLG-12*) (Figs. 2–3, S1 Table). These results would indicate that these traits are truly quantitative and teh variance explained by the shared QTL is less significant than that explained by loci elsewhere in the genome that segregate independently.

### Application to breeding

Performing association mapping on a panel of adapted breeding lines rather than on a diversity panel provides the opportunity to apply the results directly to breeding programs. Unlike the results from studies using diversity panels, our association mapping results can be readily used to identify favorable or unfavorable haplotypes that are currently segregating in our elite breeding material. These haplotypes could be used to determine the most suitable parents for crossing in order to exploit transgressive segregation and/or to increase the frequency with which favorable haplotypes appear in the progeny. MAS for favorable haplotypes among the progeny would allow us to increase breeding efficiency and decrease cost by reducing the number of plants advanced to the next generation of breeding or that need to be phenotyped. In this way, we aim to increase the rate of genetic improvement by increasing gain from selection.

MAS based on the haplotypes identified in an association mapping study such as ours necessitates a strong association between that haplotype and the phenotype-of-interest, and, ideally, a large effect of the haplotype on the phenotypic variation of the trait. When the association of haplotype and phenotype is non-perfect, as would be expected in many cases, there will be an r^2^ value at or above which marker assisted selection would be more efficient than phenotypic selection, i.e., at or above this value, the increased efficiency of marker based selection would be expected to outweigh the drag on gain from selection that would result from mistakenly eliminating favorable individuals, or vice versa. Below this threshold, phenotypic selection would be preferable. Breeders generally expect marker assisted selection accuracy to be high (> = 95%) for traits with major genes or that are easy to phenotype. However, when a trait is laborious, time consuming, or technically difficult and/or expensive to phenotype, a lower accuracy ∼70% could be acceptable to breeders [18].

We used PLINK to identify haplotypes that were associated with the significant peaks identified in our GWAS for three of our phenotypes that are routinely collected by breeders—flowering time, plant height, and yield (S4–S6 Tables). In many breeding programs in Southeast Asia, short stature, early flowering time (to allow for plant of multiple crops per year), and, of course, high yields are desirable breeding goals [19], so we therefore focus on performing selection to increase the frequency of favorable alleles at QTLs associated with these traits in our population.

#### Flowering time

For flowering time in the dry season, 17 significant haplotypes were identified, all spanning the region of the large-effect QTL on chromosome three, *qFL-3*. The haplotypes ranged from 2–124 SNPs in length, and spanned regions ranging from 233 bp—900.4 Kb (S4 Table). The early-flowering variant of haplotype H1 was the single most significant haplotype identified, and explained 34% of the phenotypic variance. Haplotype H1 spanned 58 SNPs and 202.9 Kb. The most significant haplotype associated with late-flowering was the late variant of haplotype H3, which consisted of 8 SNPs spanning 49.3 Kb, and explained ∼33% of the phenotypic variance. In the wet season, 22 significant haplotypes were identified, all of which also co-localized with *qFL-3* (S4 Table).

**Fig 4:**
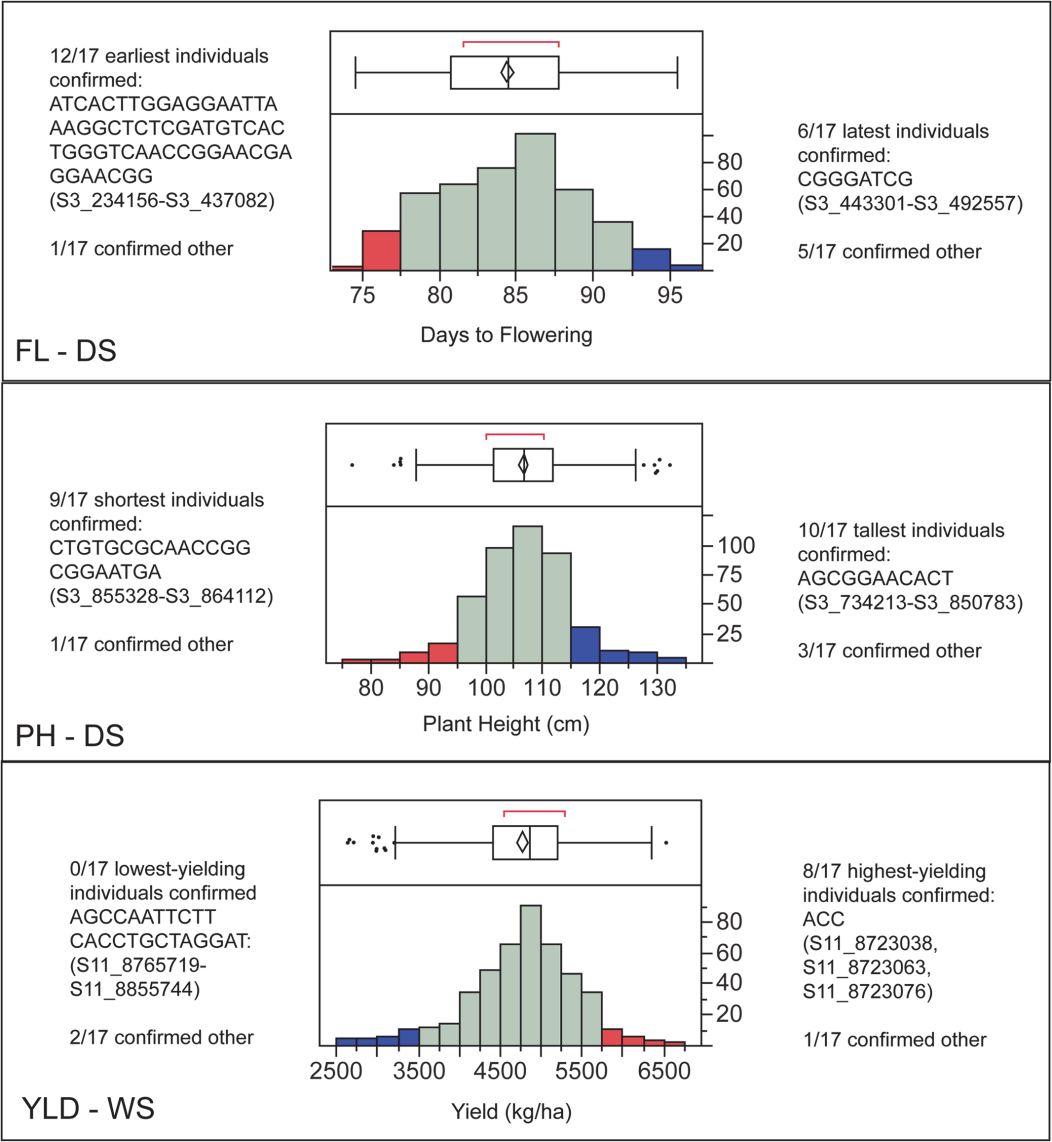
Phenotypic distributions for flowering time in the dry season (top), plant height in the dry season (middle), and yield in the wet season (bottom), showing the most desirable 5% of individuals (red) and least desirable 5% of individuals (blue). The most significant haplotypes associated with each end of the distribution are shown to the left and right of the histogram with the number of individuals in the best or worst 5% that carry the respective haplotypes. "Confirmed other" refers to individuals that were known NOT to carry the most significant haplotype. Individuals that were neither confirmed to carry the significant haplotype or confirmed to carry other haplotypes had missing data at one or more SNPs that did not preclude the possibility of the individual carrying the significant haplotype.

Of the 5% earliest flowering individuals in the dry season (17/342), 12 were confirmed to carry the early H1 haplotype (see S4 Table for exact SNPs/alleles). Only 1 out of the 17 individuals was confirmed *not* to have this haplotype. The other four individuals had missing data in the region of the haplotype which did not preclude the presence of the early H1 haplotype, but prevented confirmation of haplotype presence. For the 5% latest flowering individuals in the dry season, 6/17 were confirmed to have late haplotype H3, and 5/17 were confirmed to have other haplotypes (Fig. 4). Due to this high degree of correlation and the large effect of these haplotypes, a breeder could potentially perform either positive selection for early flowering or negative selection against late flowering using a set of linked SNPs derived from either of these haplotypes.

#### Plant height

For plant height in the dry season, 10 haplotypes were identified, all of which co-localized with *qPH-3* and *qFL-3*. The haplotypes ranged from 3–35 SNPs in length, and spanned between 4.2 and 416.2 Kb. The short stature variant of haplotype H2 was the most significant haplotype associated with shortness, and was 22 SNPs in length, spanned 8.8 Kb, and explained ∼12% of the phenotypic variance. The tall-stature variant of haplotype H1 was the most significant for tallness. H1 was 11 SNPs in length, spanned 116.6 Kb, and explained ∼10% of the phenotypic variance (S5 Table). For the 5% shortest individuals, 9/17 were confirmed to carry the H2 short haplotype, and only 1 was confirmed to carry a different haplotype. For the 5% of tallest individuals, 10/17 were confirmed to carry the H1 tall haplotype, and only 3/17 were confirmed to carry a different haplotype (Fig. 4). These results for the tall haplotype, in particular, suggest that markers linked to haplotype H1 could be developed by breeders to cull undesirably tall individuals from their program.

For the wet season, only one haplotype (H1) was identified that co-localized with qPH-2, the plant height QTL identified in the WS GWAS. H1 was 3 SNPs in length, spanned 7 bp, and explained about 7% of the phenotypic variance (S5 Table). All 12 of the shortest 5% of individuals that did not have missing data at the three SNPs that make up the haplotype were confirmed to carry the short H1 haplotype. Of the 5% tallest individuals, 4/17 had the tall stature H1 haplotype, and the 8/17 had the short haplotype. These data suggest that this haplotype would also be most useful for performing negative selection, i.e., the tall variant of H1 could be used to select against tall individuals in the wet season (Fig. 4).

#### Yield

Two significant haplotypes were identified each for the dry season and the wet season for grain yield. In the dry season, both significant haplotypes were associated with lower yield and were located at the top of chromosome six, co-localizing with a GWAS peak that fell just below our significance threshold. The most significant of these two haplotypes, H2, was 8 SNPs long, spanned a region of 47.9 Kb, and explained ∼7% of the phenotypic variance (S6 Table). Of the 5% lowest yielding individuals, 3/17 were confirmed to carry this haplotype, and 7/17 were confirmed to carry a different haplotype. Of the 5% best yielding, individuals, however, only 1/17 individuals carried this haplotype, while 13/17 were confirmed to carry other haplotypes.

The results are more interesting for the wet season. Two significant haplotypes (H1 and H3) were identified on chromosome 11, one of which, H1, was associated with higher yields. The H1 high-yield haplotype consisted of only 3 SNPs, spanned 38 bp, and explained ∼9% of the phenotypic variance (S6 Table). Of the 5% highest yielding individuals in the wet season, 8/17 were confirmed to carry high-yielding haplotype H1, and only 1/17 individuals were confirmed to carry a different haplotype, suggesting this could be a particularly useful haplotype for performing positive selection for high yield in the wet season (Fig. 4). Low yielding haplotype H3 spanned 24 SNPs and ∼90 Kb, and explained ∼6% of the phenotypic variance, however it was not possible to confirm its presence among any of the 5% lowest yielding individuals due to missing data at the relevant loci. 2/17 of the lowest yielding individuals were confirmed to carry alternative haplotypes.

### Candidate genes

To identify candidate genes underlying the above QTLs/haplotypes of interest, we searched rice genome browsers and existing literature at regions that were either near the most significant GWAS SNPs reported in S1 Table, or in the regions spanned by the significant haplotypes under selection (i.e., early flowering, short stature, high yields) reported in S4–S6 Tables (materials and methods). For flowering time, the large effect QTL identified on chromosome 3 (*qFL-3*) corresponds to the location of the QTL *Hd9* identified in early QTL mapping studies [20]. While there were a number of possible candidate genes underlying *qFL-3* (S7 Table), the *OsMADS50* gene (LOC_Os03g03100), an important activator of flowering in rice, and a homolog of *Arabidopsis* gene the *SUPPRESSOR OF OVEREXPRESSION OF CONSTANS1* (*SOC1*) was identified as the best candidate. QTL for plant height (*qPH-3*) and culm length (*qCulmL-3*) also co-localized with *qFL-3*, suggesting that *OsMADS50* may also play a role in the expression of these traits as well as for flowering time. Indeed, rice plants in which this gene was knocked down using RNAi exhibited elongated internodes, suggesting true pleiotropy may underlie these traits in rice [21].

*OsMADS50* co-localizes with both *Hd9* and our peak association marker (S1 and S7 Tables), but it does not fall within the most significant flowering time haplotype (S4 Table)[21,22,23,24]. This suggests that a second genetic feature located ∼800 Kb downstream of *OsMADS50* is a target of selection in this breeding population. No obvious candidate genes for flowering time were identified within this haplotype but the data suggest this is potentially a novel flowering time-related QTL.

Several known, important, rice flowering time genes also underlie the flowering time QTL identified on chromosome 6 (*qFL-6*), including *Hd1*, *RFT1* (a.k.a. *FT-3*), and *FT-2*. *Hd1*, however, is ∼6Mb away from the peak QTL marker, and thus is not the best candidate (S7 Table) [25,26,27]. *RFT1*, a homolog of an Arabidopsis florigen gene, is located directly beneath the association peak (S1 Table) [25,26], while *FT-2* is located nearby ∼11 Kb away. The flowering time QTL on chromosome 8 (*qFL-8*) also corresponded to a previously mapped QTL, *Hd5*/*DTH8*, which encodes a *HAP3* subunit of the CCAAT-box-binding transcription factor and has been shown to play an important role in early flowering [28,29,30]. Another possible candidate gene that encodes a putative cullin protein lies nearby; cullins are F-box protein subunits with the potential to regulate flowering time (S7 Table)[31]. Additional candidate genes for the flowering time QTL identified in the wet season only (*qFL-2*, *qFL-9*) are shown in S7 Table.

*OsMADS50* is also the best candidate gene for the plant height QTL on chromosome 3 (*qPH-3*), as mentioned previously, although several other candidates were also identified (S7 Table)[21]. *qPH-6* and *qPH-8* were both were very near putative zinc finger domain containing proteins that provide likely candidates. *qPH-2* was near a putative GA encoding gene, which makes a good candidate for this wet season QTL (S7 Table).

No best single candidate gene was identified for the wet season yield QTL on chromosome 11 (*qYLD-11*), but several putative stress tolerance genes were identified in the vicinity of the significant haplotype which warrant further investigation, see S7 Table.

### Toward Genomic Selection for rice improvement

The lack of perfect association between the haplotypes described above and their respective phenotypes, as well as the limited number of QTL identified for many of the phenotypes in this study, highlight the complexity of the genetic architecture underlying many agronomic traits in rice. Even the haplotypes for flowering time, for which we identified a large effect QTL, did not explain 100% of the phenotypic variance. This poses a problem for the implementation of MAS in rice breeding programs. While genetic gain could potentially be increased over phenotypic selection alone, the probability of eliminating favorable individuals or selecting unfavorable individuals will limit overall breeding progress.

Genomic selection (GS), first described by Meuwissen et al., in 2001 could solve this problem [32]. Instead of making selections based on a subset of previously identified significant QTL, in a genomic selection breeding scheme, selections are based on the output of statistical models that are fit using all available high density genotyping data [15,16,32]. Our recently published study in PLoS Genetics explores the efficacy of performing genomic selection in this same population [33], but the GWAS results presented here by themselves also suggest that GS could be an effective strategy for rice improvement. The GWAS results are also informative for thinking about how GS could best be implemented for this population. The lack of any large-effect QTL for many of the agronomic traits studied here suggest that for some agronomic traits, linear, additive statistical models such as RR-BLUP could be the most effective means of predicting breeding value. The presence of large-effect QTL for other traits such as flowering time, however, suggest that for other traits in rice, non-additive genomic selection models and/or models that can account for differences in marker variance may be more accurate than the simple RR-BLUP models. The results also suggest that a model in which the large effect QTL identified from this GWAS are fit as fixed effects could further improve accuracy. Simulation experiments and a preliminary studies in wheat and cattle have shown this to be the case for high heritability traits with large fixed effects [14,17,34].

GWAS have been used to identify many genes that underlie a variety of agronomic traits in rice [8,9,10,11,12,13,35,36]. Here we performed a GWAS on a breeding population of elite inbred rice lines for the IRRI irrigated rice breeding program in order to a.) more directly apply the results to MAS in this breeding population, and b.) inform the genomic selection results presented in the companion paper. The successful identification of haplotypes for flowering time, plant height, and grain yield, as well as the elucidation of the genetic architecture underlying a variety of the agronomic traits in this population suggests that this can be a powerful strategy for subsequent rice improvement.

## Materials and Methods

### Plant material

363 elite breeding lines were selected for genotyping from the International Rice Research Institute (IRRI) irrigated rice breeding program based on the planned inclusion of the lines in the 2011 Multi-Environment Testing Program and in the 2011 and 2012 Replicated Yield Trials (RYT) at IRRI (Los Baños). Approximately half of the lines were also included in the 2009–2010 RYTs at IRRI (S1 Table). The other lines were promoted from the observational yield trial (OYT) to the RYT in 2011. The lines were all derived from the pedigree breeding method. Information on pedigrees and selection history is presented in S8 Table.

### Phenotyping

A total of 19 agronomic, morphological, grain and yield-related traits were evaluated in the panel in 2012 (dry and wet seasons) as described in Table 1. A detailed analysis of these traits will be reported elsewhere. Briefly, all measurements were performed using two replications with 5 plants per plot, except plot yield, which was measured using a 6 m^2^ harvest area. All measurements were made on a quantitative scale, with the exception of lodging score and panicle exertion rate, which were nominal. Methods used followed IRRI’s standard evaluation system (SES) or other routinely used protocols. Predicted means and variance components were calculated using linear mixed models in Genstat v. 16. Entries were considered as fixed effects and replication and entry by replication interactions were considered as random effects. A detailed trait analysis will be reported elsewhere.

### Genotyping

#### DNA extraction

Young leaf tissue was collected from each of the 369 breeding lines from plants grown in Gutterman Greenhouse in Ithaca, NY. DNA was extracted using the Qiagen 96-plex DNeasy kit as per the Qiagen fresh leaf tissue 96-plex protocol (www.qiagen.com/HB/DNeasy96Plant).

#### Library preparation

384-plex genotyping-by-sequencing (GBS) libraries were prepared using the protocol by Elshire *et al*. 2011 [37], as described previously in Spindel and Wright *et al* 2013 [38].

#### GBS data analysis

SNPs were discovered and called from the raw 384-plex GBS data using the TASSEL3.0 GBS pipeline with physical alignment to the MSU version 6.0 Nipponbare rice reference genome using Bowtie2, as described in Spindel and Wright *et al* 2013 [38,39,40] (S4 Fig.). The IRRI breeding materials genotyped here are a collection of multi-parent related and unrelated inbred lines, so the GBS-PLAID algorithm for imputation, which was developed specifically for imputation of biparental rice mapping populations, was not useful [38]. Imputation of missing data was instead performed using the TASSEL3.0 FastImputationBitFixedWindow plugin with default settings [41]. The algorithm works by dividing the entire SNP dataset into small SNP windows, then identifying the most similar inbred line within each window to fill the missing data. The algorithm takes advantage of small IBD regions shared between pairs of inbred lines in the collection; if the window from the closest neighbor has more than 5% difference from the line being imputed, the data point is left as missing [41]. The imputation error rate using this algorithm was estimated for each chromosome in our dataset by masking a fraction of the un-imputed allele calls and comparing the imputed and actual calls. The average imputation error rate across the twelve rice chromosomes was estimated in this way to be less than 1%.

SNPs that still had 10% or more data missing after imputation (or call rates of < 90%) were removed from the dataset along with all monomorphic SNPs, for a total SNP set of 73,147 SNPs. A second genotype dataset was also obtained in which we removed all monomorphic SNPs, SNPs with call rates < 75%, and SNPs with minor allele frequencies (MAF) < 0.05 for a total of 71,710 SNPs. Individuals with more than 60% missing data were dropped from both datasets, which resulted in the removal of six individuals that failed sequencing for the total of 363 genotyped lines used throughout the study (S4 Fig.).

Both final datasets were then transformed from nucleotide genotype coding (i.e., 'A', 'C', 'T', 'G') to numeric coding (1, 0, -1 for class I homozygotes, heterozygotes, and class II homozygotes, respectively) in order to facilitate statistical analysis. The minimal remaining missing data were filled using the numeric genotype means of each line in order to perform PCA and GWAS (S4 Fig.).

### Subpopulation structure analysis

The majority of the 363 lines were characterized apriori from pedigree records to belong to the *indica* or *indica-admixed* subpopulation groups. In order to identify outlier individuals belonging to the *japonica* or *japonica-admixed* groups, principle components analysis (PCA) was performed in R (version 3.0.1, function prcomp) using the imputed 73,147 SNP dataset. The first principle component of high density SNP data in rice can separate the *indica* and *japonica* subgroups [11], so by plotting the first four principle components using JMP Pro 10, 13 *japonica* outliers were identified as pulled in a tight cluster apart from the rest of the 350 lines (S1A Fig.). These 13 lines were removed from the dataset, and a second PCA was performed using the same methodology as the first to identify any admixed outliers, i.e, outlier lines containing greater percentages of *japonica* derived SNPs. By plotting the first four principle components of the second PCA, another 18 lines were judged to be outliers and removed from the dataset, leaving a total of 332 lines to be used for the cross-validation experiments (S1B Fig.). A third PCA was performed using the remaining 332 to confirm that there were no additional subpopulation outliers. Percent variance explained by the first four principle components for each PCA was produced from the R function output.

### Association mapping

Association mapping was performed in R using the GEMMA implementation of the standard linear mixed model y = Wα + Xβ + u + ε, where y is a vector of phenotypes, W = (w1,..wc) is an n × c matrix of covariates (fixed effect), α is a c-vector of the corresponding coefficients, β is the marker effect size, X is a matrix of allele dosages for the imputed variants, u is a vector of random effects and ε is a vector of errors. u is multivariate normal, ∼ MVNn(0, λτ¯^1^ K), with τ¯^1^ as the variance of the residual errors, λ the ratio between the two variance components, and K the m × m genomic relationship matrix. ε is ∼ MVNn(0, λτ¯^1^In) where In is an n × n identity matrix [42]. K was also calculated using GEMMA (parameter-gk = 2). For additional details on the GEMMA implementation of the mixed model, see Zhao et al., 2012 [43].

GWAS models that included the first principle component (PC) as a covariate were compared to models that did not include the first PC using TASSEL v. 4.3.1, and were found not to differ significantly, therefore, we did not include the first PC as a covariate when building the GEMMA model. This makes sense given the small percent variance explained by any of the principle components after removal of outliers (see results). The QQ plots (S2 Fig.) also indicate that the GEMMA model fit the data well. The significance of the GWAS results were determined using a Wald test, and the significance threshold determined by a false discovery rate of 0.1 using the R function p.adjust (method = BH) [44]. Allele effects were calculated as the difference between the average trait value for all lines that were homozygous for the major allele (AA) and the average trait value for all lines that were homozygous for the minor allele (BB) for a given SNP. The percent variance explained by all significant SNPs discovered in each season was output from GEMMA. The percent variance explained by each individual significant SNP was calculated as the squared correlation between the phenotype and genotype of the SNP [45]. QQ plots and Manhattan plots were generated using R. all other plots were produced using the program JMP v. 10.0.

### Haplotype Analysis

Haplotype blocks were calculated for flowering time, plant height, and yield for both seasons of data in PLINK (command—blocks). For all phenotypes except yield in the dry season, the input genotype files contained all SNPs that passed the GWAS significance threshold. For yield in the dry season, the input genotype file contained all SNPs with p-value < = .0001. The resulting PLINK blocks file was then used to calculate the haplotype frequencies in PLINK using the—hap-freq command. To test haplotypes for significant association with phenotype, we used the PLINK—hap-assoc command.

For each trait, the most significant haplotype associated with either low phenotypic value or high phenotypic value (e.g. early flowering and late flowering) was identified. The proportion of either the individuals with the 5% lowest phenotypic values or the 5% highest phenotypic values that carried the most significant haplotypes associated with the respective high/low phenotype were calculated.

### Candidate gene analysis

Candidate genes were identified for the flowering time, plant height and yield QTLs, three of the most important traits to breeders. Previously identified QTL that co-localized with the QTL mapped in this study were identified using the Gramene QTL data archive (http://archive.gramene.org/qtl/). To ascertain whether QTL had been cloned, literature searches were performed. Regions covered by significant haplotypes (S4–S6 Tables) and SNPs were also searched for candidates using the MSU v.7 rice genome browser (http://rice.plantbiology.msu.edu/cgi-bin/gbrowse/rice/). All results are reported in S7 Table.

## Supporting Information

S1 Fig Plots of the first four principle components of selected elite breeding lines using 73,147 SNPs. (**A**) Initial principle components analysis (PCA) using 363 lines to identify 13 *japonica* outliers (purple). (**B**) PCA on remaining 350 lines after removing 13 outliers identified in A. An additional 18 outliers (purple) were subsequently identified and excluded.
(EPS)

S2 Fig QQ plots for final model for all 19 traits including plant height (PH), flowering date (FLW), culm length (CulmL), number of effective tiller or panicle per plant (PN), flag leaf length (FlgLL), flag leaf width (FlgLW), flag leaf area (FlgLA), number of spikelets per panicle (SPn), number of filled grain per plant (FGP), grain length (GrL), grain width (GrW), grain length-breadth ratio (LBR), lodging score (LG), peduncle length (PedL), panicle exertion rate (Exs), 1000 grain weight (1000GW), yield per plant (YPP), and grain yield per plot (YLD), for both the dry (DS) and wet (WS) seasons. (PDF)

S3 Fig Manhattan plots for final model for all 19 traits including plant height (PH), flowering date (FLW), culm length (CulmL), number of effective tiller or panicle per plant (PN), flag leaf length (FlgLL), flag leaf width (FlgLW), flag leaf area (FlgLA), number of spikelets per panicle (SPn), number of filled grain per plant (FGP), grain length (GrL), grain width (GrW), grain length-breadth ratio (LBR), lodging score (LG), peduncle length (PedL), panicle exertion rate (Exs), 1000 grain weight (1000GW), yield per plant (YPP), and grain yield per plot (YLD), for both the dry (DS) and wet (WS) seasons. Dashed line shows the 0.1 FDR significance threshold. Plots with no dashed line did not have any SNPs that passed the significance threshold.
(PDF)

S4 Fig Diagram of genotyping process. 384-plex GBS was used to discover and call SNPs on 369 elite inbred rice lines from the IRRI irrigated rice breeding program. SNPs were discovered and called from the raw GBS data using TASSEL3 with physical alignment to the MSU version 6 Nipponbare rice reference genome using Bowtie2 (yellow boxes). Imputation of missing data was then performed using the TASSEL3 fastimputationbitfixedwindow plugin (materials and methods). After imputation, custom python scripts (green boxes) were used to remove SNPs with call rates < 90% or <75% (two different datasets), remove monomorphic SNPs, remove SNPs with MAF < 0.05, drop individuals with more than 60% missing data, and finally, convert the ACTG nucleotide calls to numeric coding (i.e., homozygote class I = 1, homozygote class II = -1, heterozygote = 0). After genotypes were converted to numeric format, remaining missing genotype values were filled using the numeric line mean.
(EPS)

S1 Table All significant QTL identified by the GWAS. Table shows up to the top five most significant SNPs identified for each season for each QTL, for each trait along with the major and minor alleles for the SNP and the allele effect of the major allele. rs = SNP identifier, p_wald = Wald Test p-value.
(XLSX)

S2 Table The total percent variance explained estimates and standard errors for each trait in the dry and wet season. (CSV)

S3 Table The percent variance explained values for all significant SNPs for all traits in the dry and wet seasons, where a SNP was considered significant if it passed the FDR threshold of 0.1. (CSV)

S4 Table Significant flowering time haplotypes. Locus = haplotype number assigned by PLINK, NANAL = number of individuals in analysis, BETA = regression coefficient, R2 = percent variance explained by haplotype, STAT = T test statistic, P = asymptotic p-value, pop_freq = frequency of haplotype in population, SNPs = SNPs that make up the haplotype. Negative BETA values indicate that the haplotype is associated with earlier flowering, positive BETA values indicate that the haplotype is associated with later flowering. The most significant haplotype associated with early flowering and the most significant haplotype associated with late flowering is highlighted for both seasons.
(XLSX)

S5 Table Significant plant height haplotypes. Locus = haplotype number assigned by PLINK, NANAL = number of individuals in analysis, BETA = regression coefficient, R2 = percent variance explained by haplotype, STAT = T test statistic P = asymptotic p-value, pop_freq = frequency of haplotype in population, SNPs = SNPs that make up the haplotype. Negative BETA values indicate that the haplotype is associated with shorter stature, positive BETA values indicate that the haplotype is associated with taller stature. The most significant haplotype associated with short stature and the most significant haplotype associated with tall stature is highlighted for both seasons.
(XLSX)

S6 Table Significant grain yield haplotypes. Locus = haplotype number assigned by PLINK, NANAL = number of individuals in analysis, BETA = regression coefficient, R2 = percent variance explained by haplotype, STAT = T test statistic P = asymptotic p-value, pop_freq = frequency of haplotype in population, SNPs = SNPs that make up the haplotype. Negative BETA values indicate that the haplotype is associated with lower yield, positive BETA values indicate that the haplotype is associated with higher yield. The most significant haplotype associated with low yield in the dry season and the most significant haplotypes associated with low yield and high yield in the wet season are highlighted.
(XLSX)

S7 Table Candidate genes for flowering time, plant height, and yield. QTL name = name of QTL from this study, gene name = published name of cloned gene, pos **(MSU 7.0)** = position in bp from the MSU v 7.0 rice genome, pos (Gramene) = Ensemble/Gramene position for cloned genes and historic (reference) QTL. Near (marker or haplotype) = the significant SNP marker(s) or haplotype(s) identified in this study nearest the identified candidate gene. Reference QTL name = historic QTL name as recorded in the Gramene QTL archive, Gramene QTL ID = accession number of QTL in the Gramene QTL archive. Gene product description = description of gene product from MSU rice genome annotation project and/or Gramene. Highlighted rows represent particularly good candidate genes.
(XLSX)

S8 Table Germplasm pedigree for 262/263 individuals included in the GWAS. One sample could not be tracked.
(XLSX)
