# Supplementary material for: Genome-Wide Association Mapping for Yield and Other Agronomic Traits in an Elite Breeding Population of Tropical Rice (Oryza sativa)
Source: PLoS One. 2015 Mar 18;10(3):e0119873. doi: 10.1371/journal.pone.0119873 (PMC4364887; doi:10.1371/journal.pone.0119873)
Supplement: S2 Fig — (PDF) [file pone.0119873.s002.pdf]

A

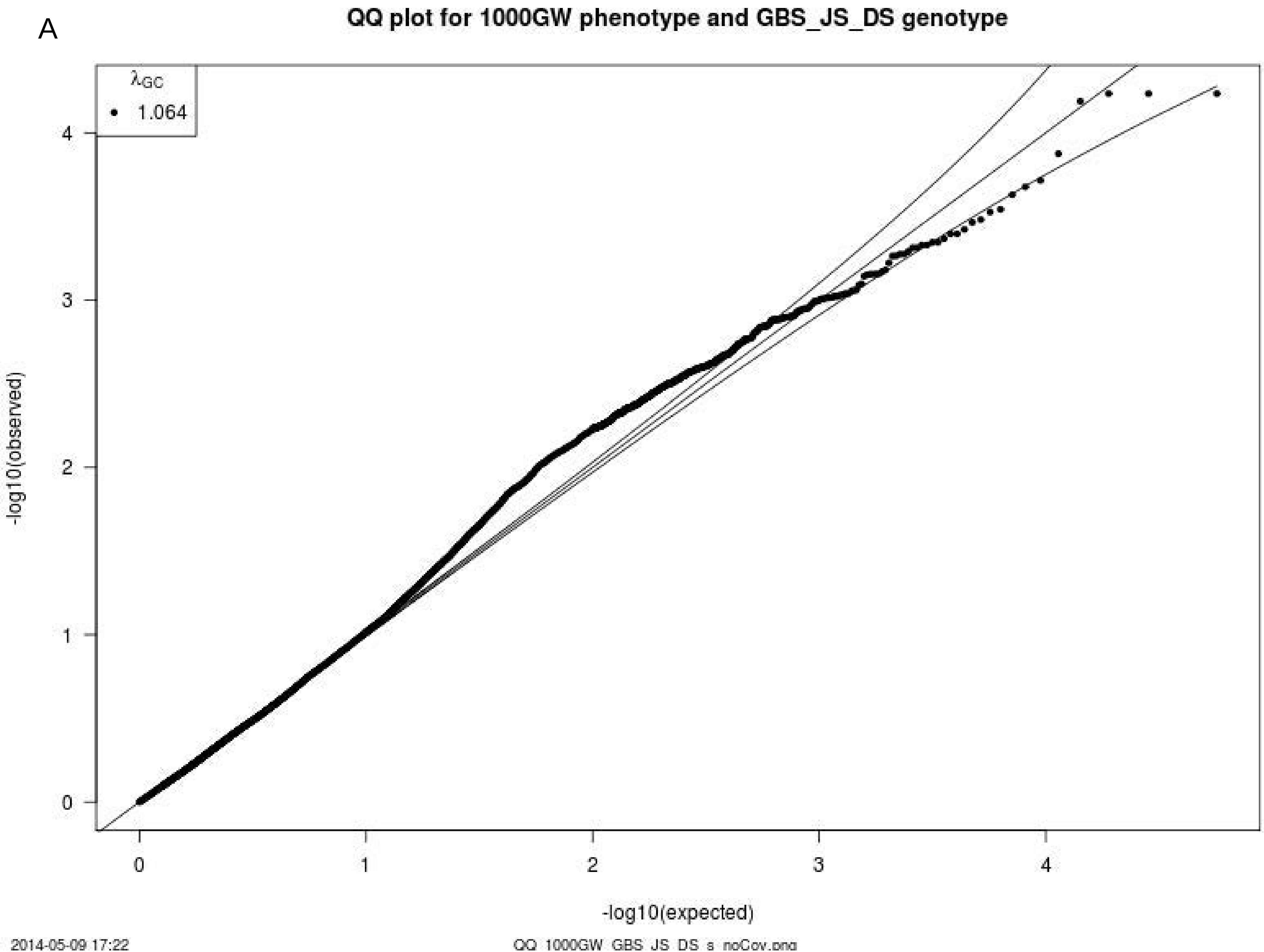

QQ plot for 1000GW phenotype and GBS_JS_DS genotype
λGC
1.064
4
3
2
1
0
-log10(observed)
0
1
2
3
4
-log10(expected)
2014-05-09 17:22
QQ_1000GW_GBS_JS_DS_s_noCov.png

B

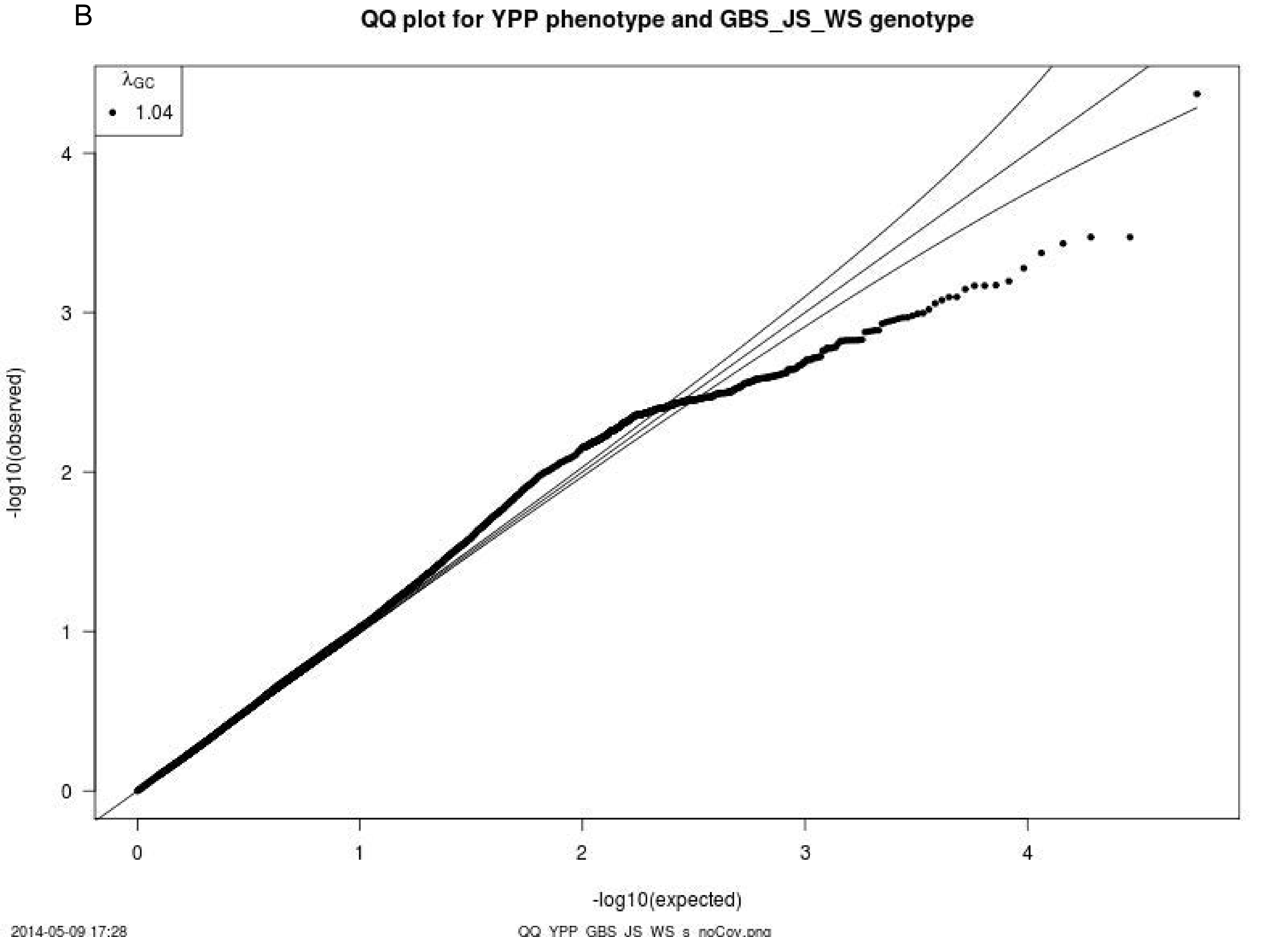
QQ plot for YPP phenotype and GBS_JS_WS genotype
λGC
1.04
-log10(observed)
-log10(expected)
0
1
2
3
4
2014-05-09 17:28
QQ_YPP_GBS_JS_WS_s_noCov.png

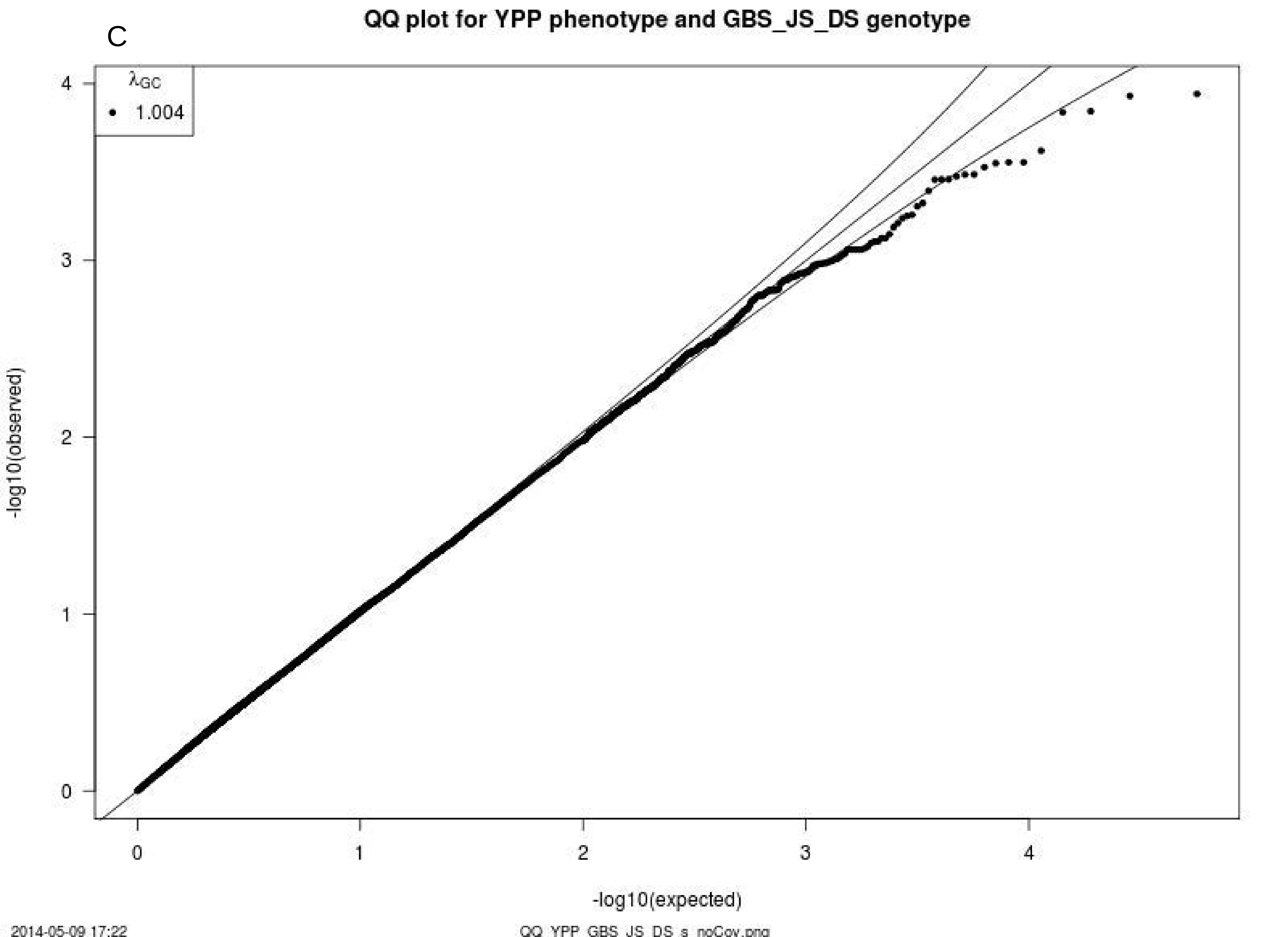
C
QQ plot for YPP phenotype and GBS_JS_DS genotype
λ_{GC}
1.004
-log10(observed)
-log10(expected)
0
1
2
3
4
2014-05-09 17:22
QQ_YPP_GBS_JS_DS_s_noCov.png

D

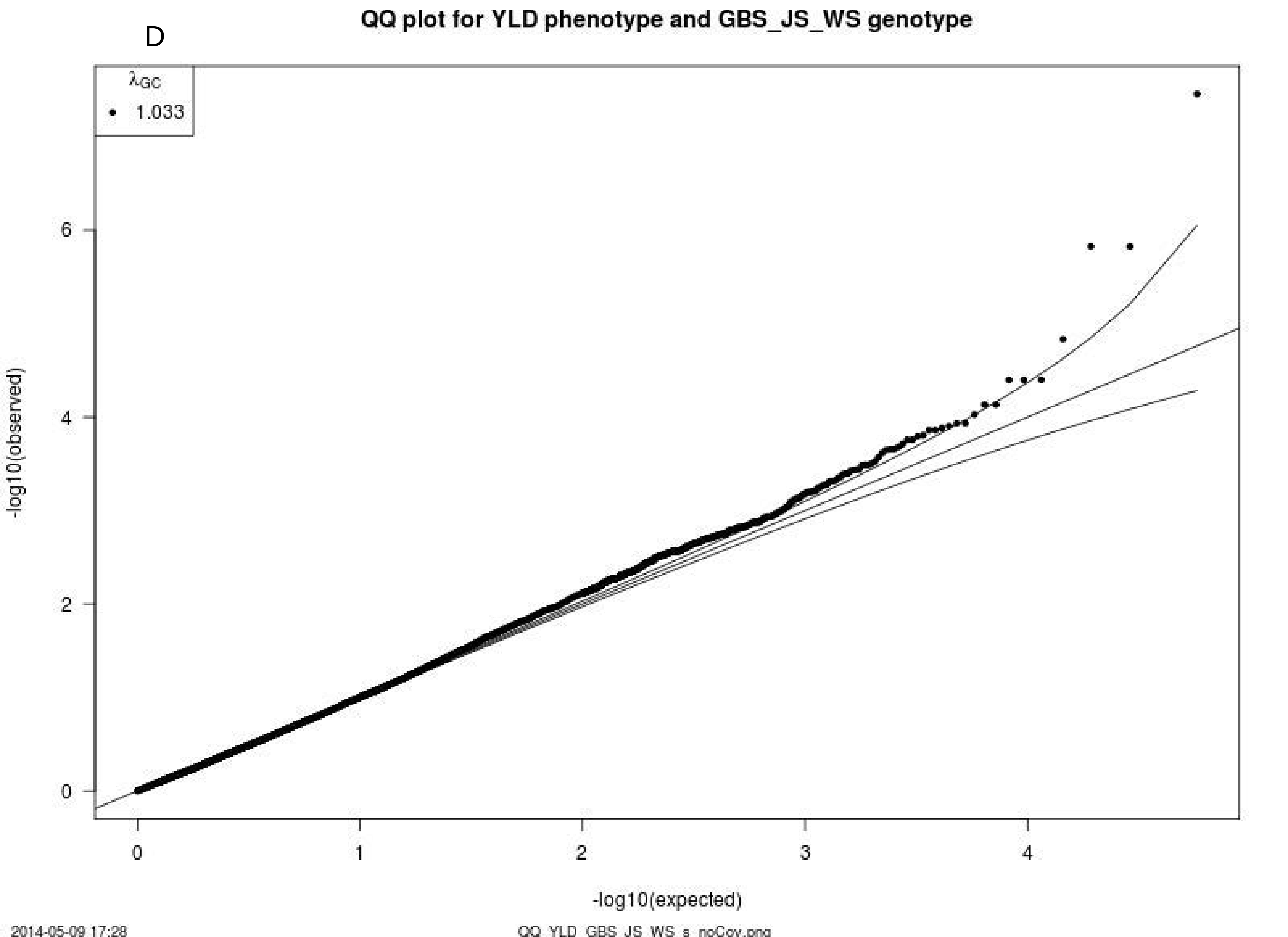

QQ plot for YLD phenotype and GBS_JS_WS genotype
λ_{GC}
1.033
-log10(observed)
-log10(expected)
0
2
4
6
0
1
2
3
4
2014-05-09 17:28
QQ_YLD_GBS_JS_WS_s_noCov.png

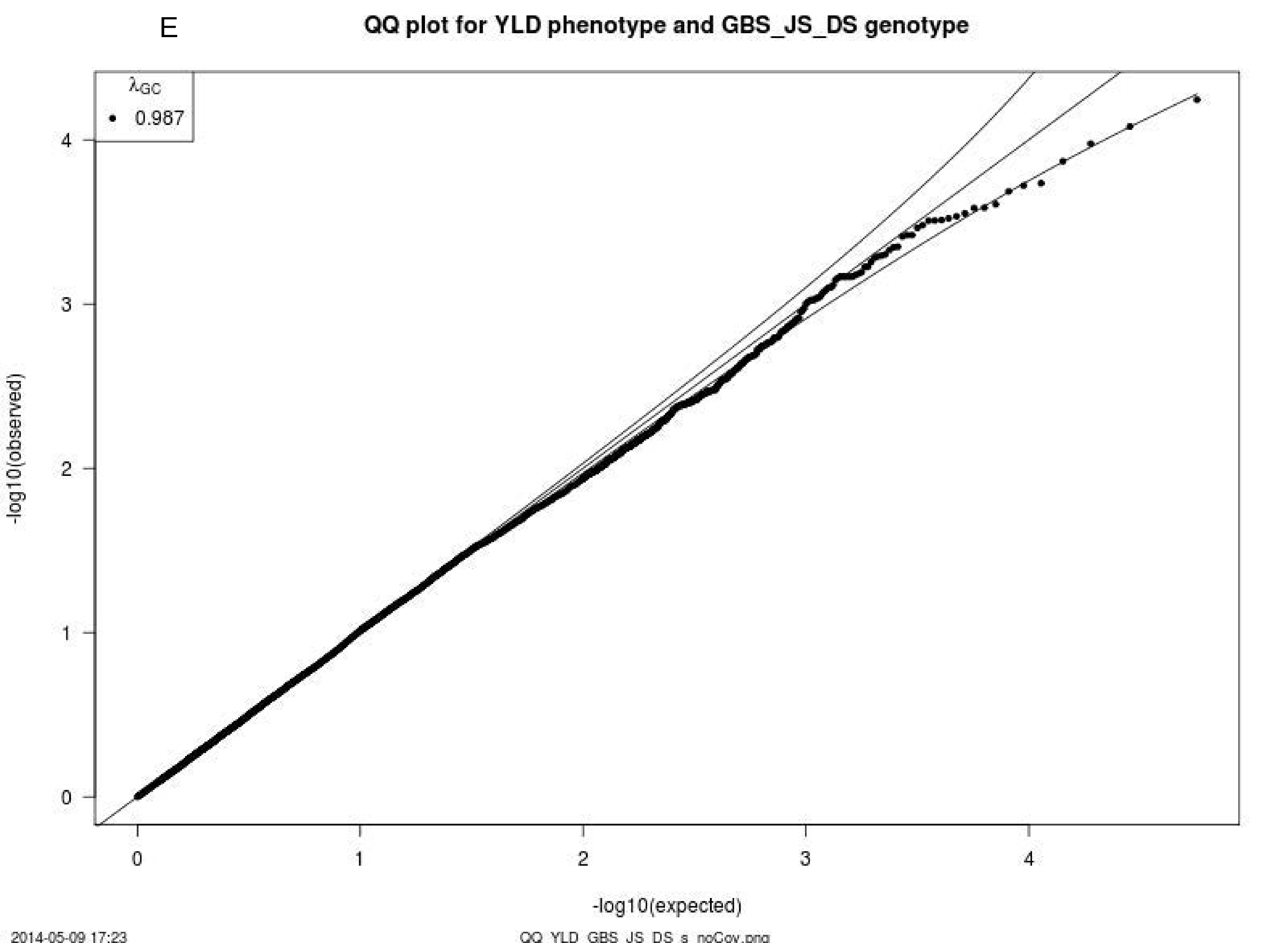
E
QQ plot for YLD phenotype and GBS_JS_DS genotype
λGC
0.987
-log10(observed)
-log10(expected)
0
1
2
3
4
2014-05-09 17:23
QQ_YLD_GBS_JS_DS_s_noCov.png

F

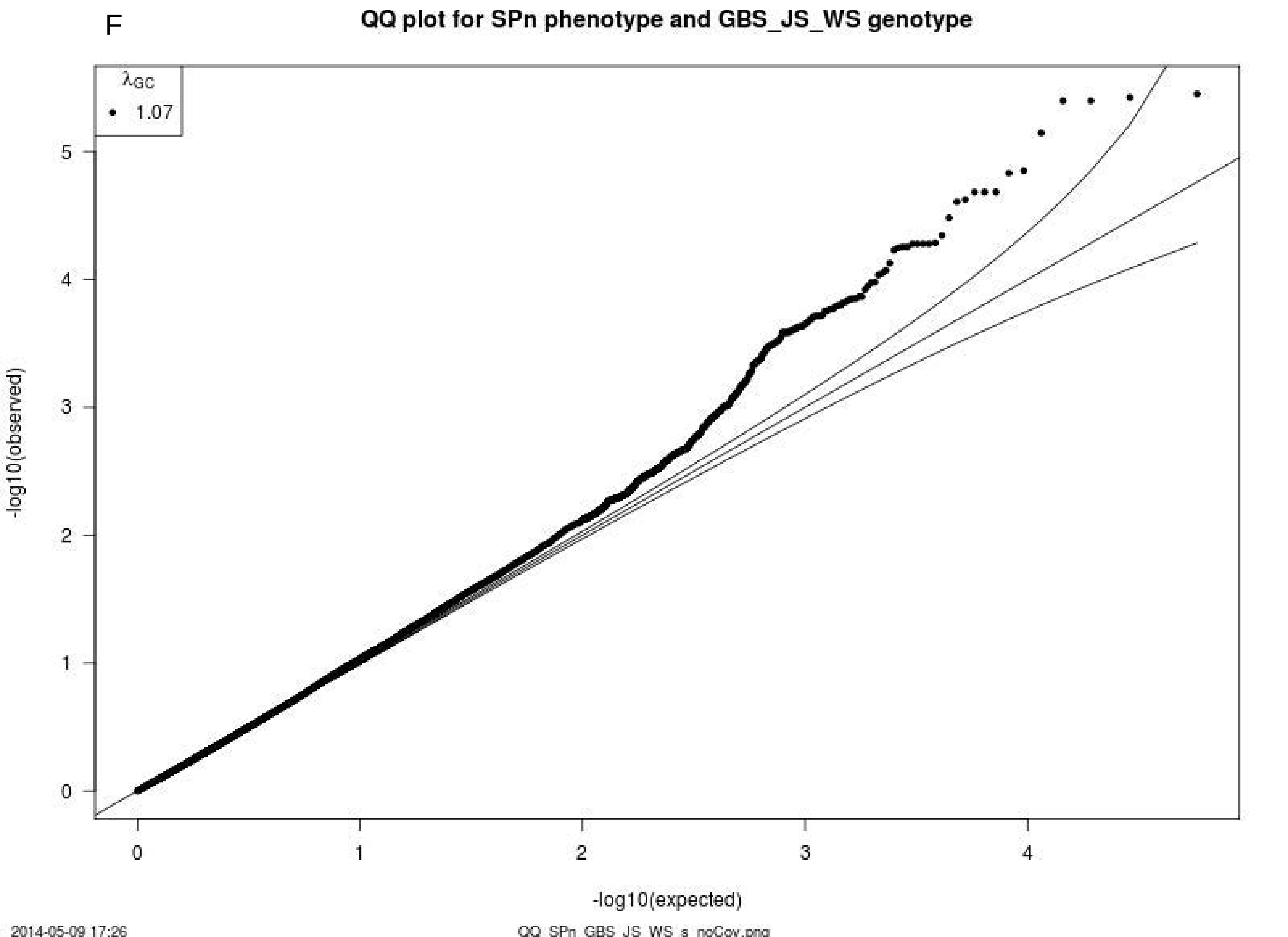
QQ plot for SPn phenotype and GBS_JS_WS genotype
λGC
1.07
5
4
3
2
1
0
0
1
2
3
4
-log10(observed)
-log10(expected)
2014-05-09 17:26
QQ_SPn_GBS_JS_WS_s_noCov.png

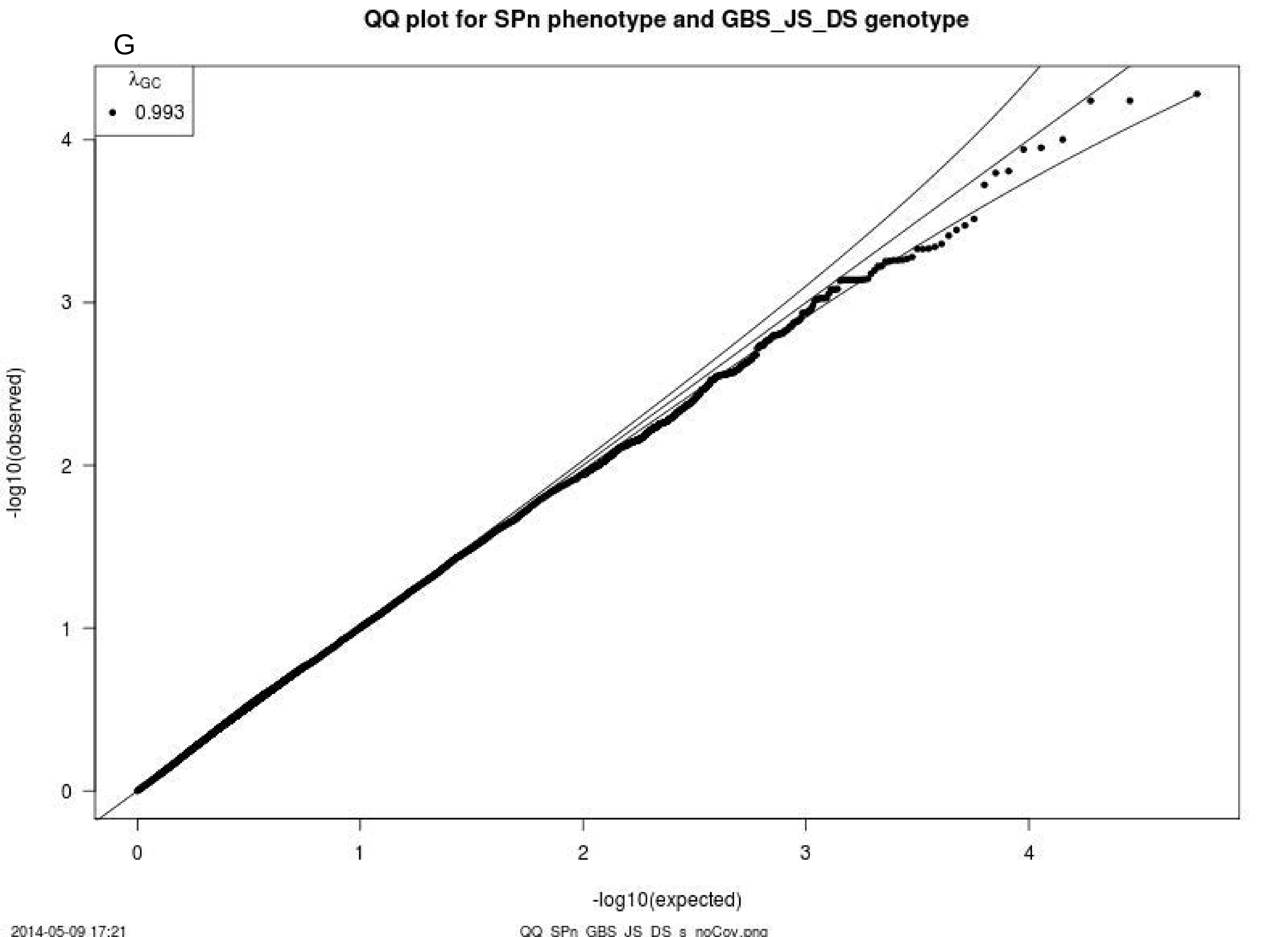

QQ plot for SPn phenotype and GBS_JS_DS genotype
G
λGC
0.993
-log10(observed)
-log10(expected)
0
1
2
3
4
2014-05-09 17:21
QQ_SPn_GBS_JS_DS_s_noCov.png

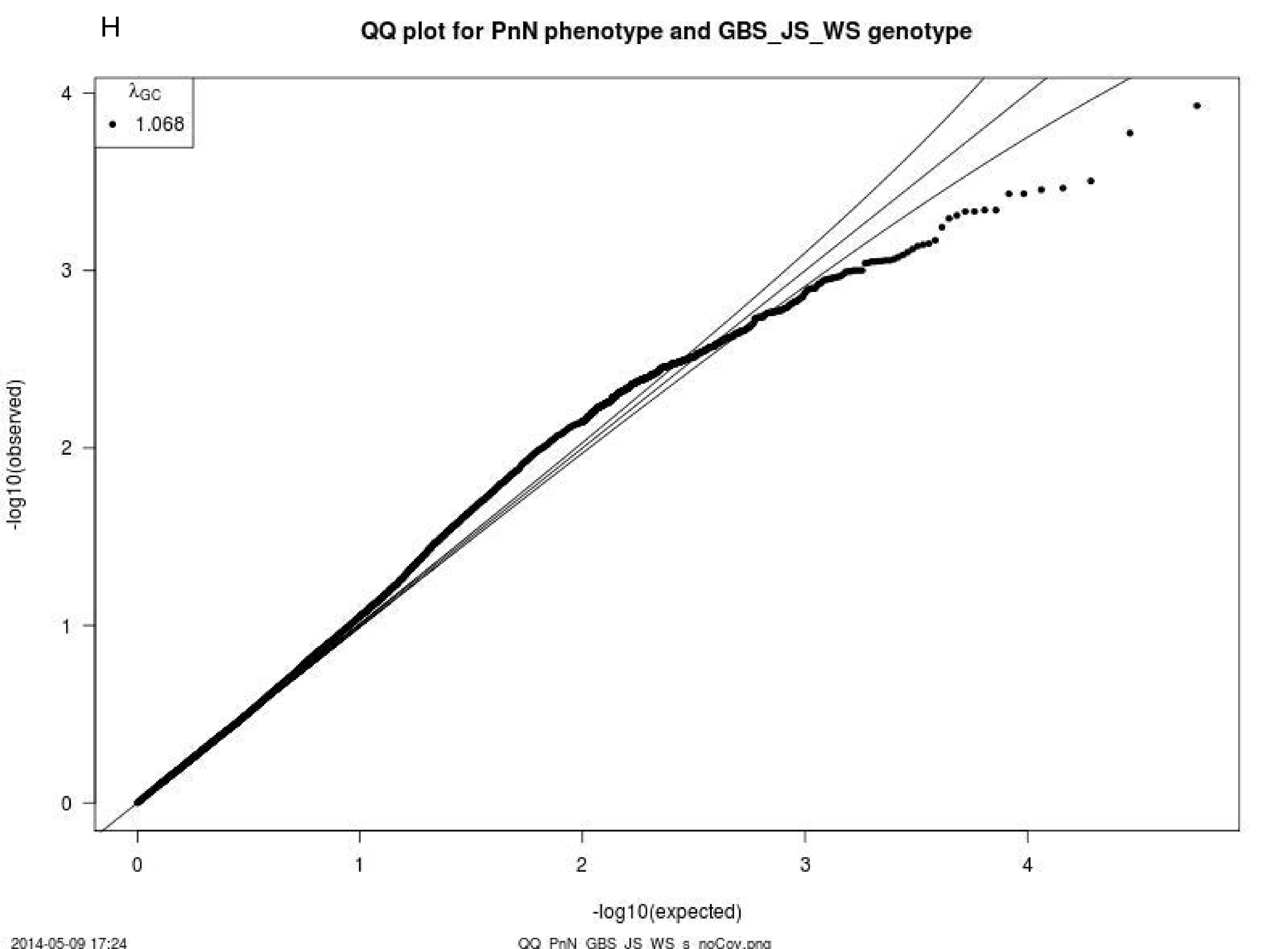
H
QQ plot for PnN phenotype and GBS_JS_WS genotype
λGC
1.068
-log10(observed)
-log10(expected)
0
1
2
3
4
2014-05-09 17:24
QQ_PnN_GBS_JS_WS_s_noCov.png

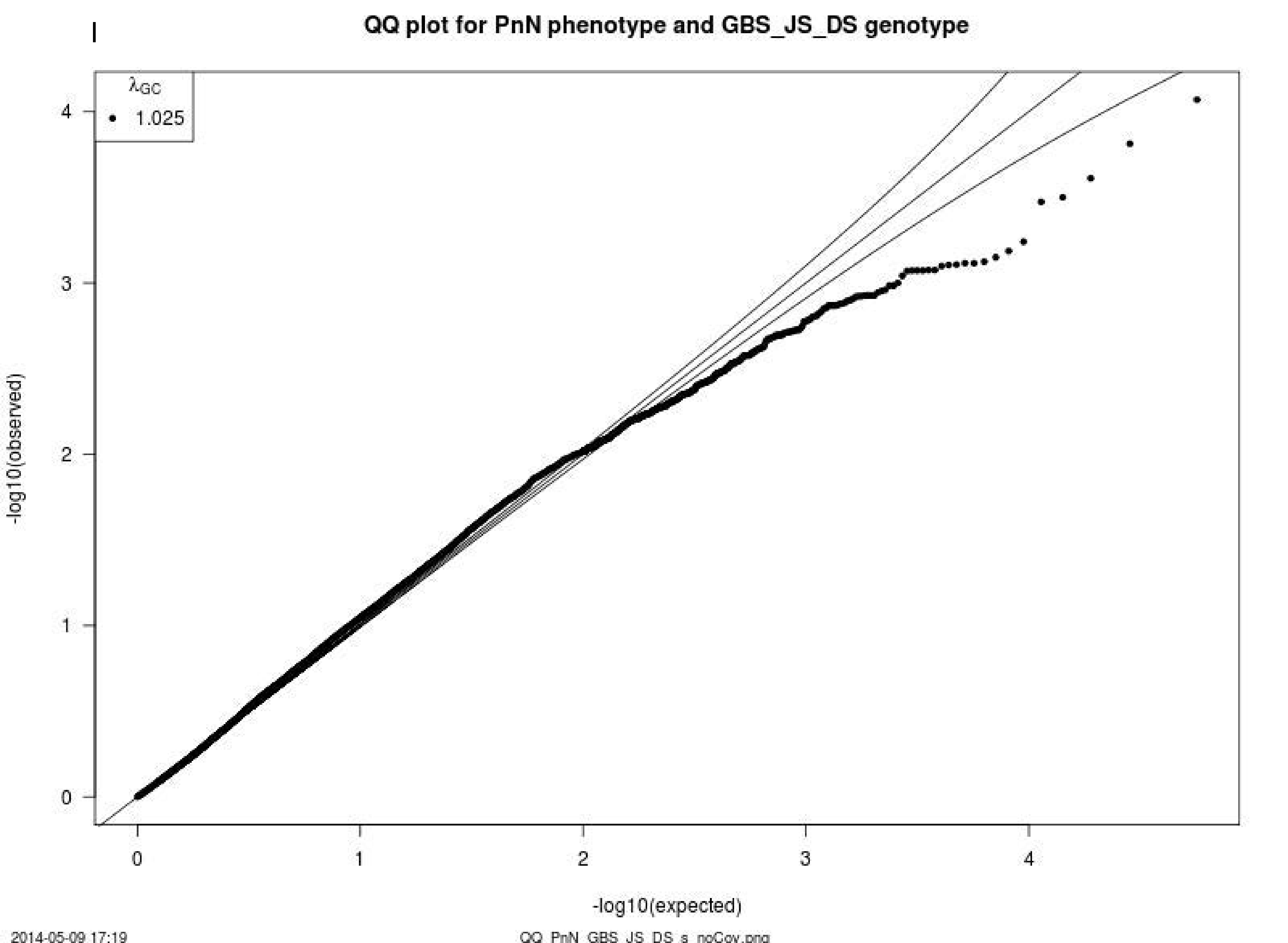
QQ plot for PnN phenotype and GBS_JS_DS genotype
λGC
1.025
-log10(observed)
-log10(expected)
0
1
2
3
4
2014-05-09 17:19
QQ_PnN_GBS_JS_DS_s_noCov.png

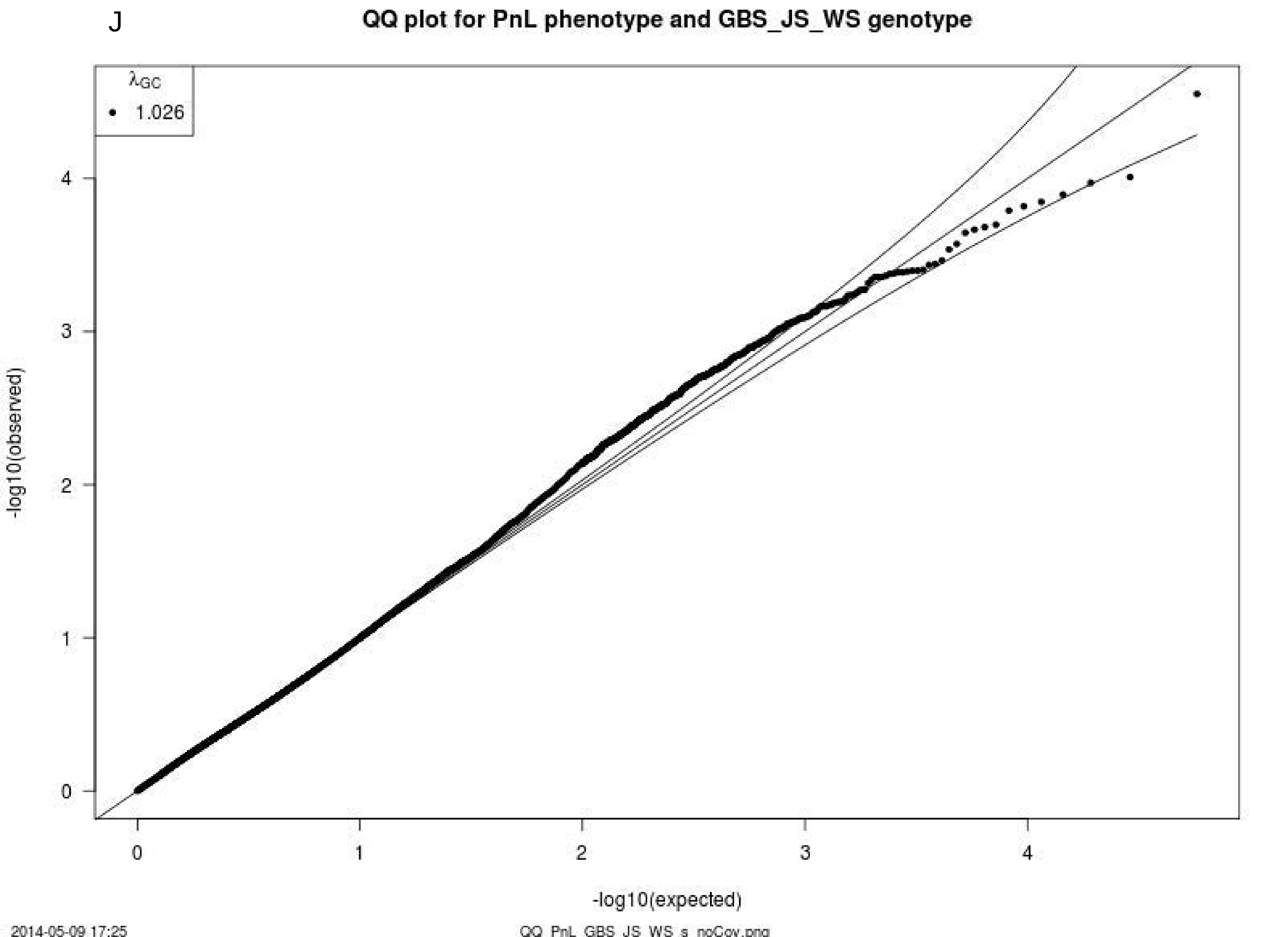
J
QQ plot for PnL phenotype and GBS_JS_WS genotype
λGC
1.026
-log10(observed)
-log10(expected)
0
1
2
3
4
2014-05-09 17:25
QQ_PnL_GBS_JS_WS_s_noCov.png

K

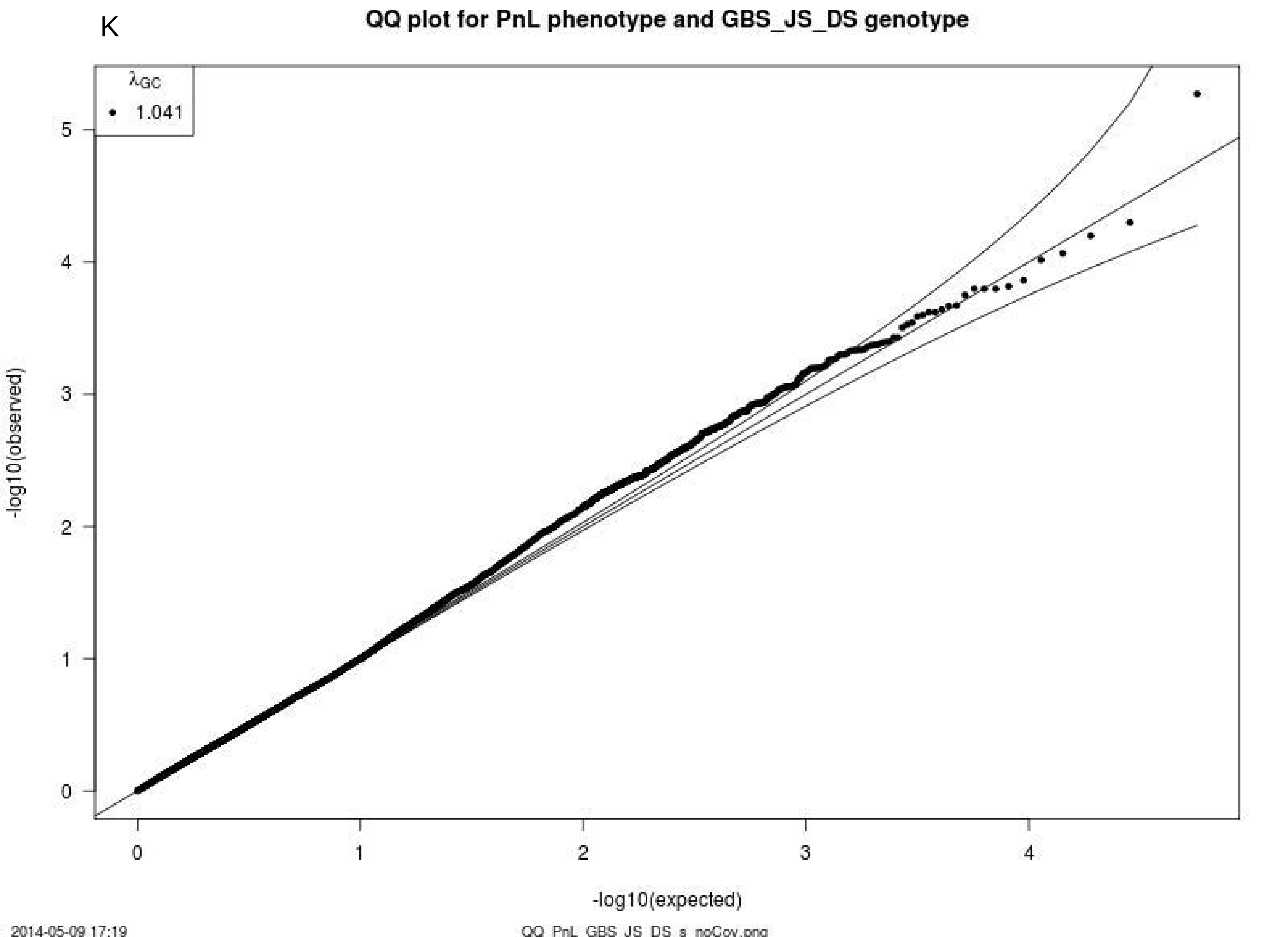
QQ plot for PnL phenotype and GBS_JS_DS genotype
λGC
1.041
-log10(observed)
-log10(expected)
0
1
2
3
4
5
2014-05-09 17:19
QQ_PnL_GBS_JS_DS_s_noCov.png

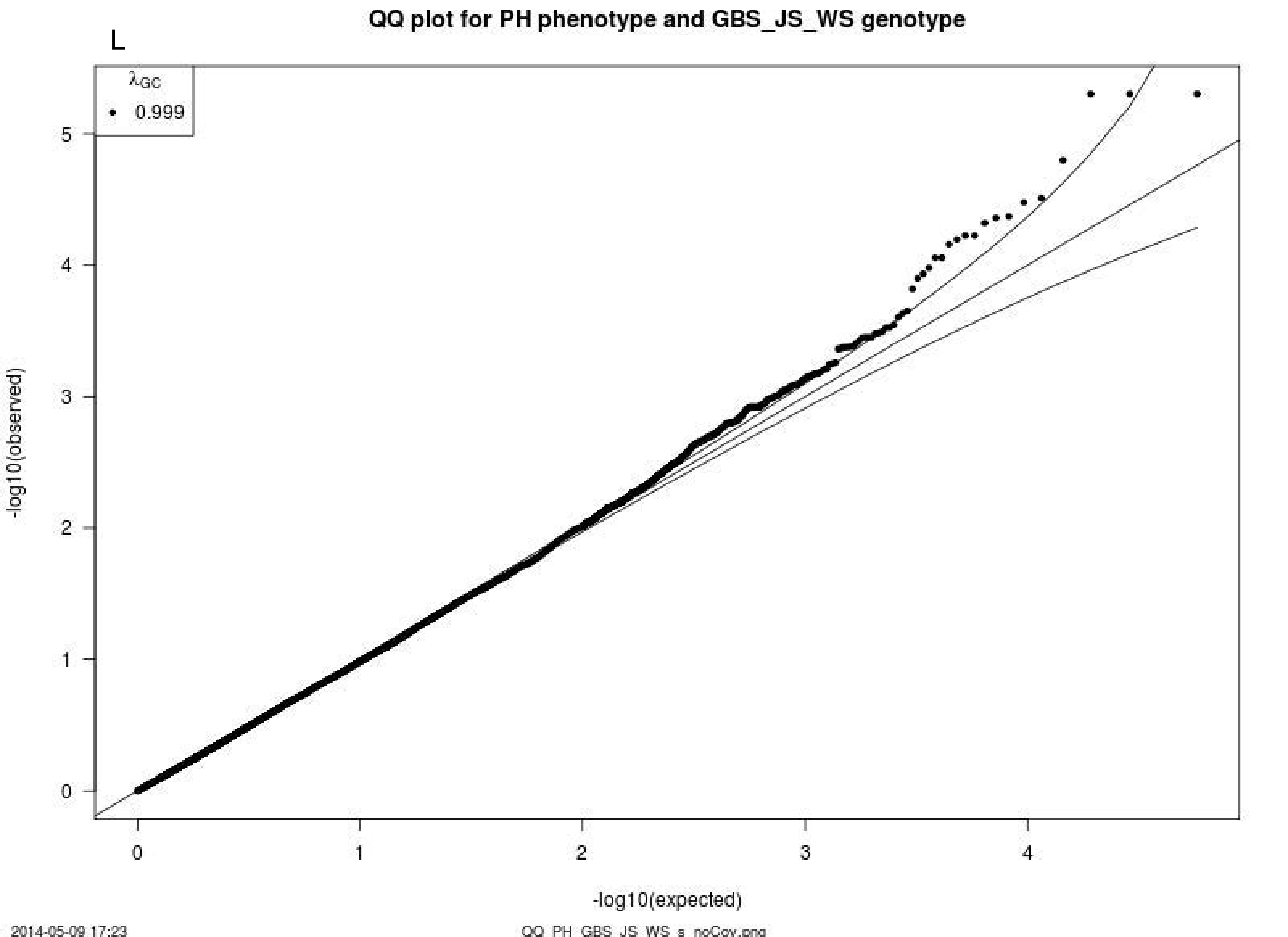
QQ plot for PH phenotype and GBS_JS_WS genotype
L
λGC
0.999
-log10(observed)
-log10(expected)
0
1
2
3
4
5
2014-05-09 17:23
QQ_PH_GBS_JS_WS_s_noCov.png

M

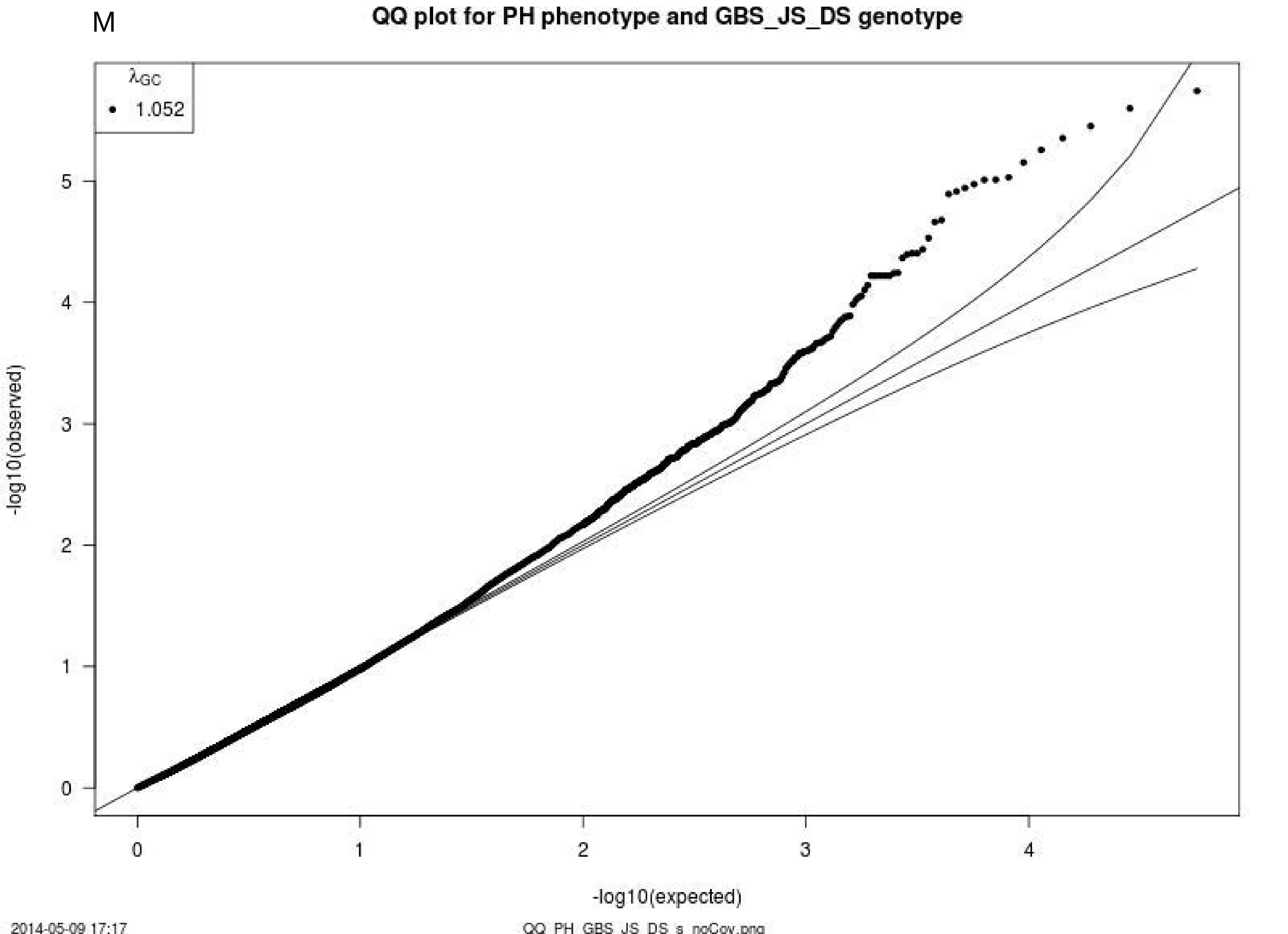
QQ plot for PH phenotype and GBS_JS_DS genotype
λGC
1.052
-log10(observed)
-log10(expected)
0
1
2
3
4
5
2014-05-09 17:17
QQ_PH_GBS_JS_DS_s_noCov.png

N

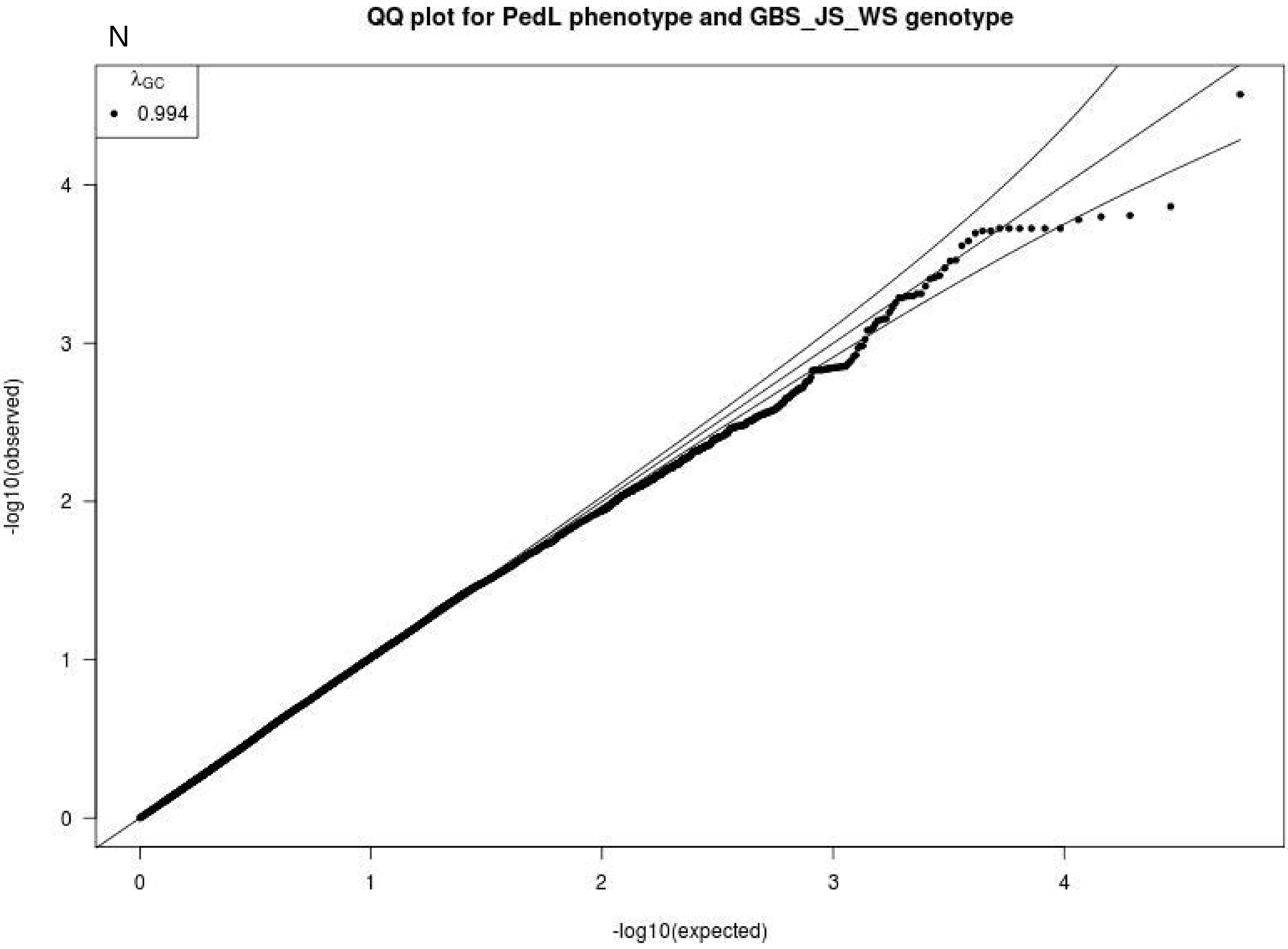
QQ plot for PedL phenotype and GBS_JS_WS genotype
λ_{GC}
0.994
-log10(observed)
-log10(expected)
0
1
2
3
4

O

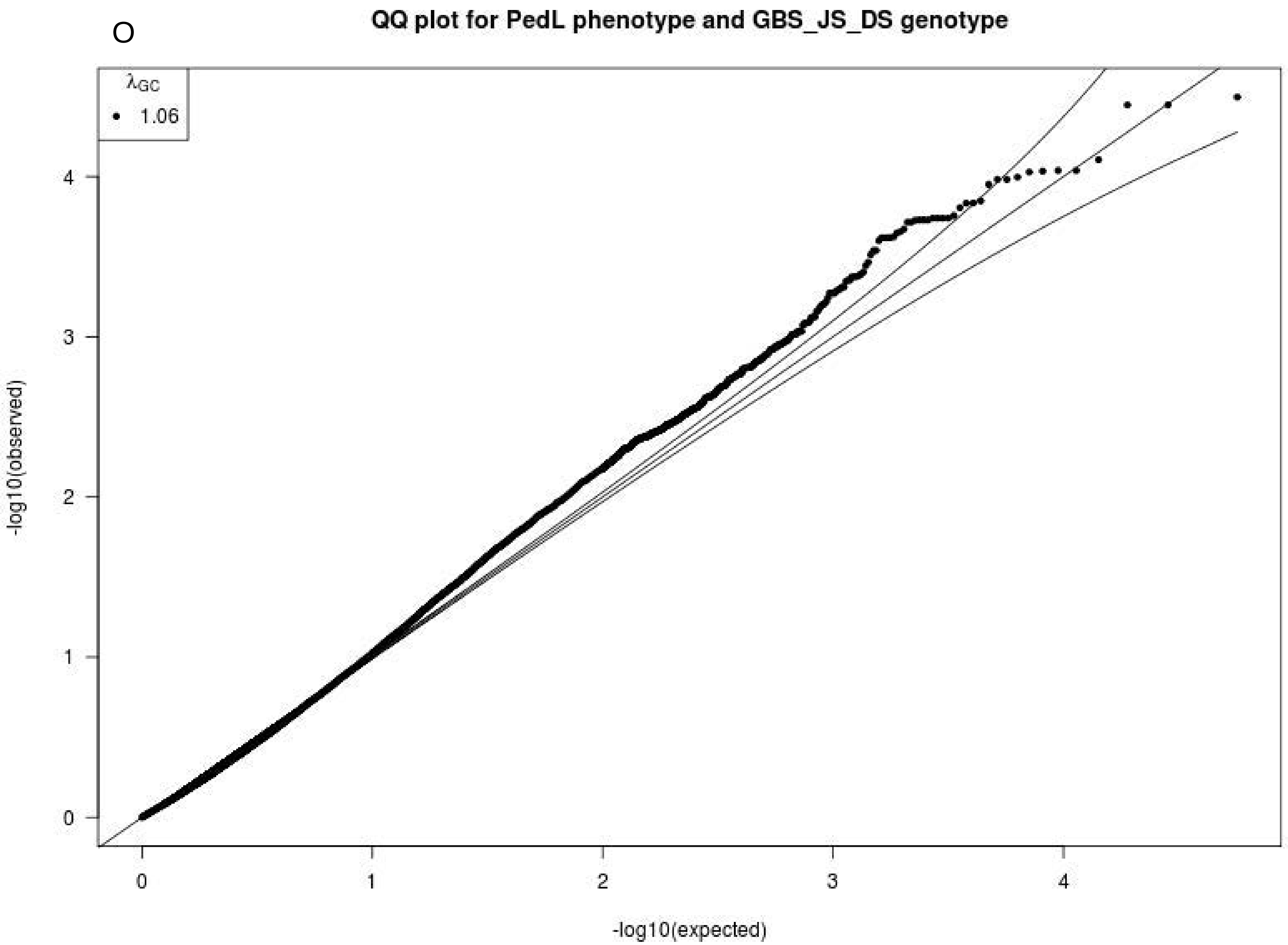

2014-05-09 17:19 QQ_PedL_GBS_JS_DS_s_noCov.png

P

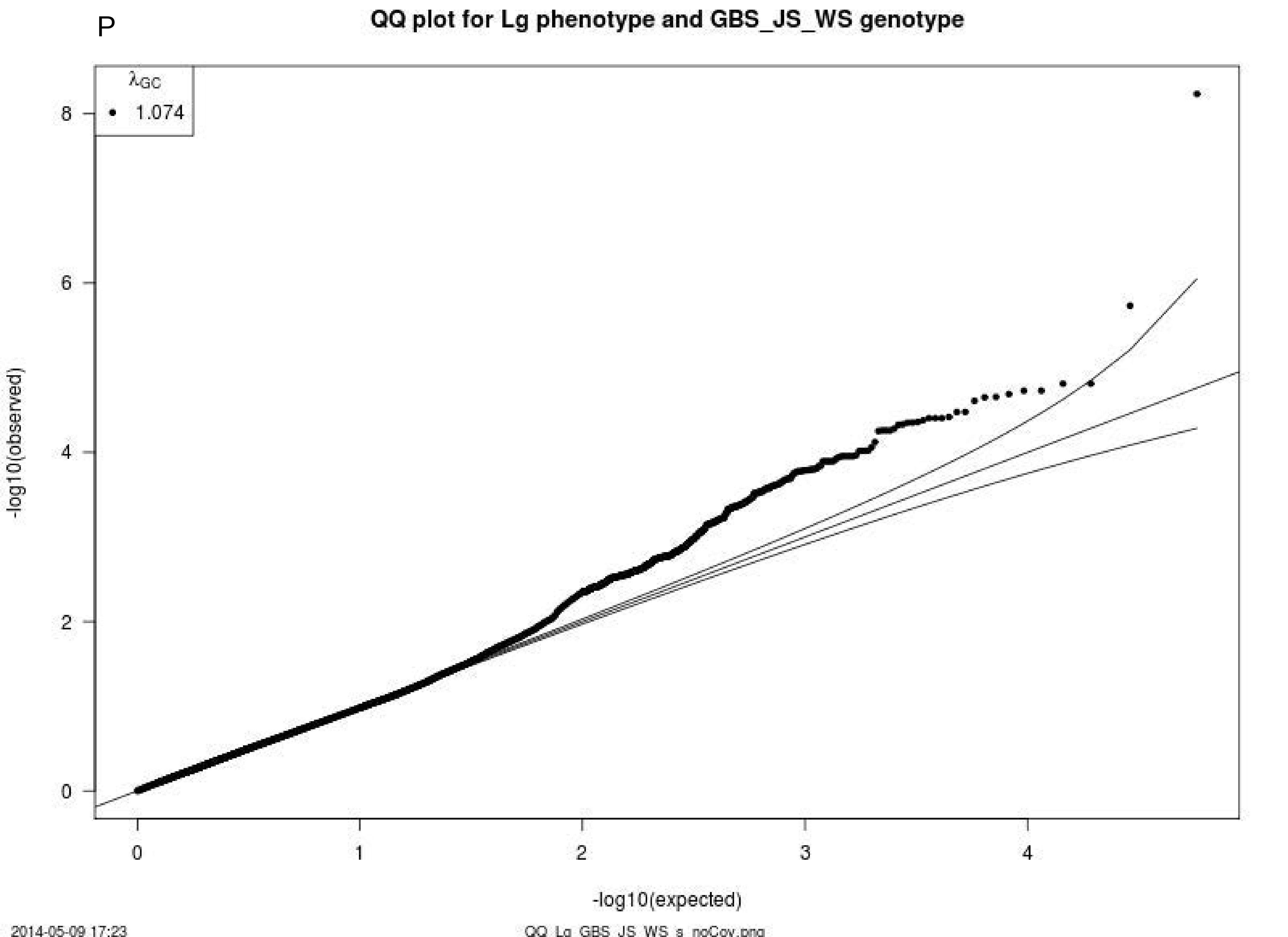

QQ plot for Lg phenotype and GBS_JS_WS genotype
λGC
1.074
-log10(observed)
-log10(expected)
0
2
4
6
8
0
1
2
3
4
2014-05-09 17:23
QQ_Lg_GBS_JS_WS_s_noCov.png

Q

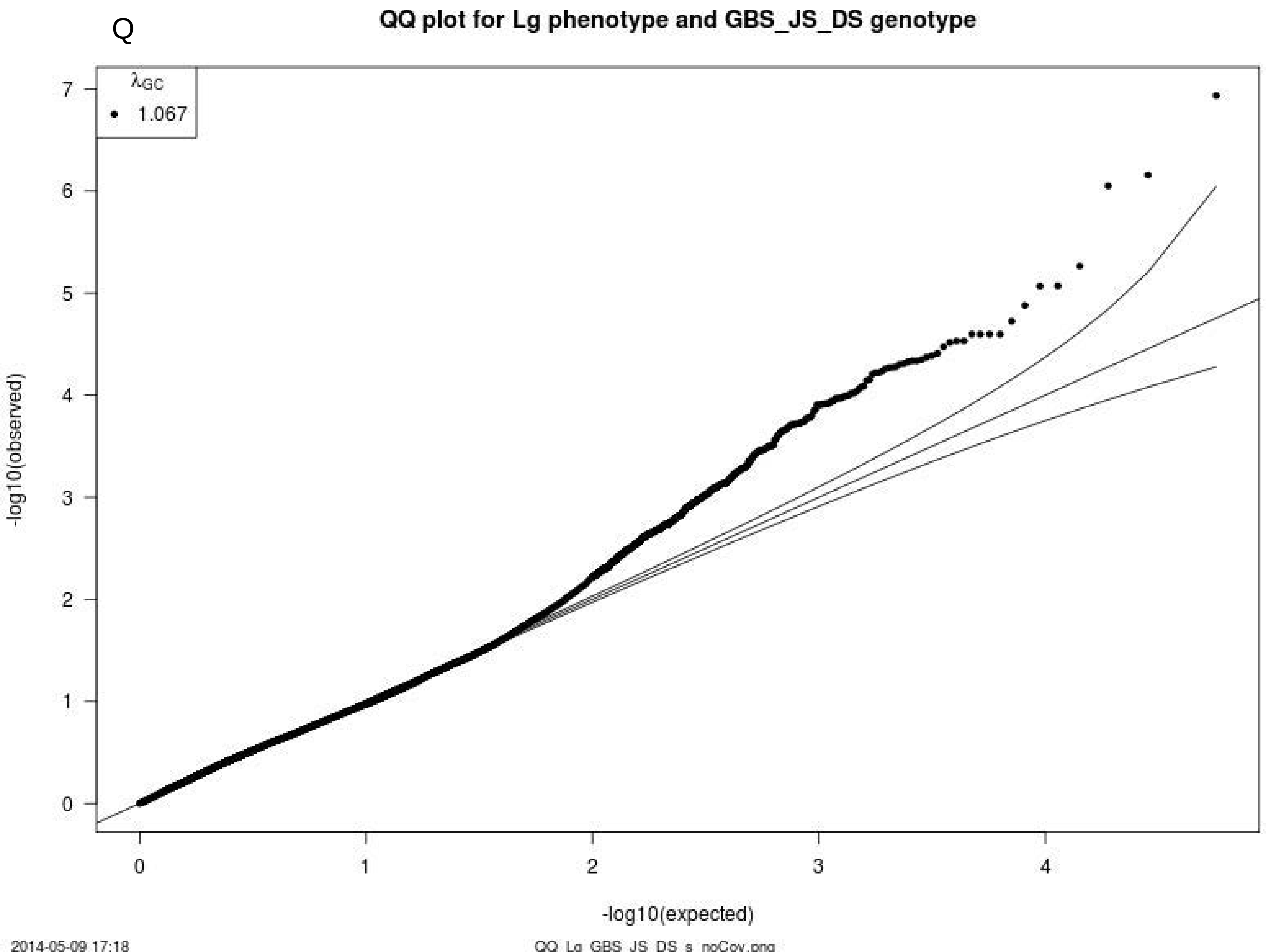
QQ plot for Lg phenotype and GBS_JS_DS genotype
λGC
1.067
-log10(observed)
-log10(expected)
0
1
2
3
4
5
6
7
2014-05-09 17:18
QQ_Lg_GBS_JS_DS_s_noCov.png

R

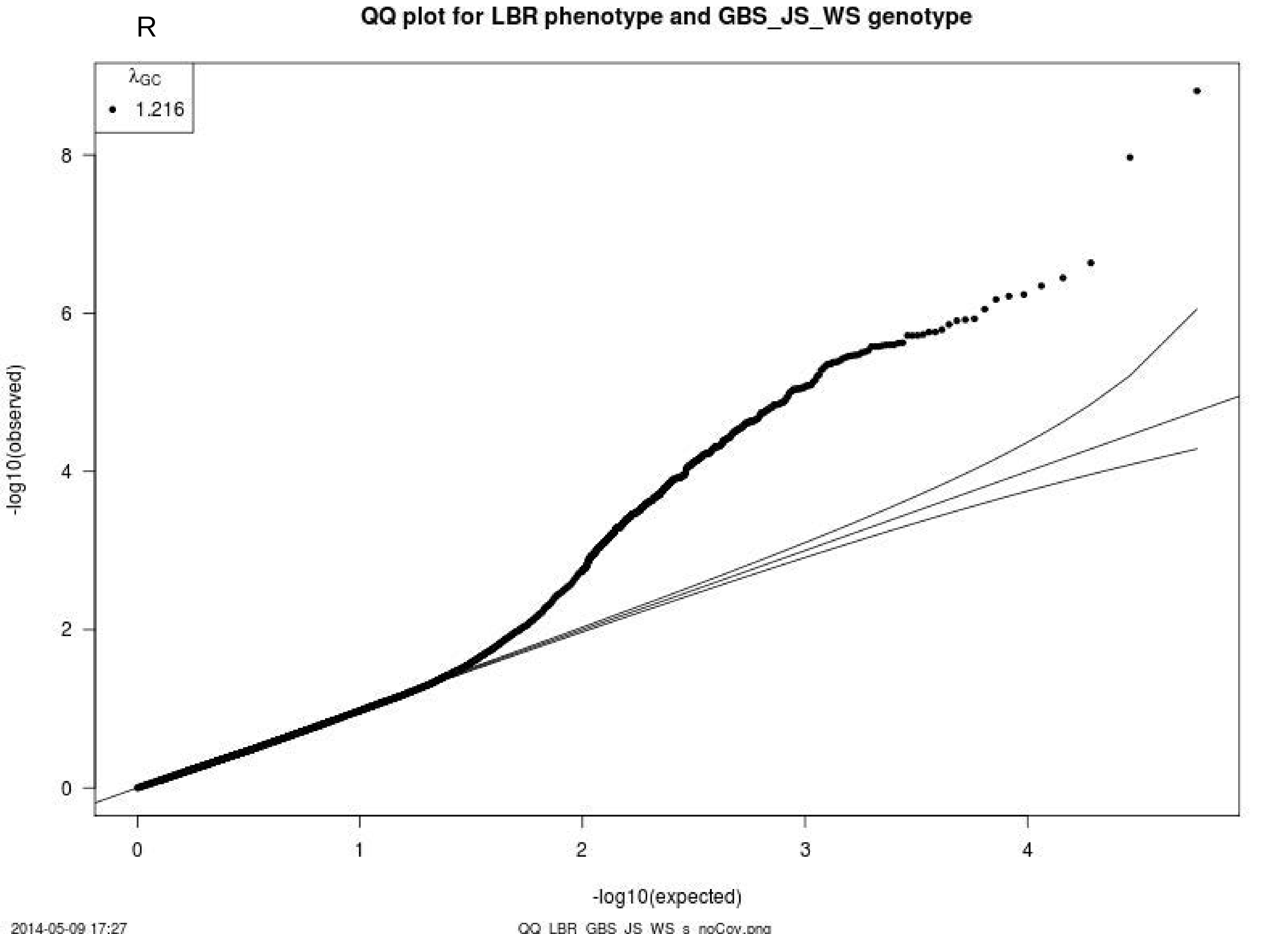

QQ plot for LBR phenotype and GBS_JS_WS genotype
λGC
1.216
8
6
4
2
0
-log10(observed)
0
1
2
3
4
-log10(expected)
2014-05-09 17:27
QQ_LBR_GBS_JS_WS_s_noCov.png

S

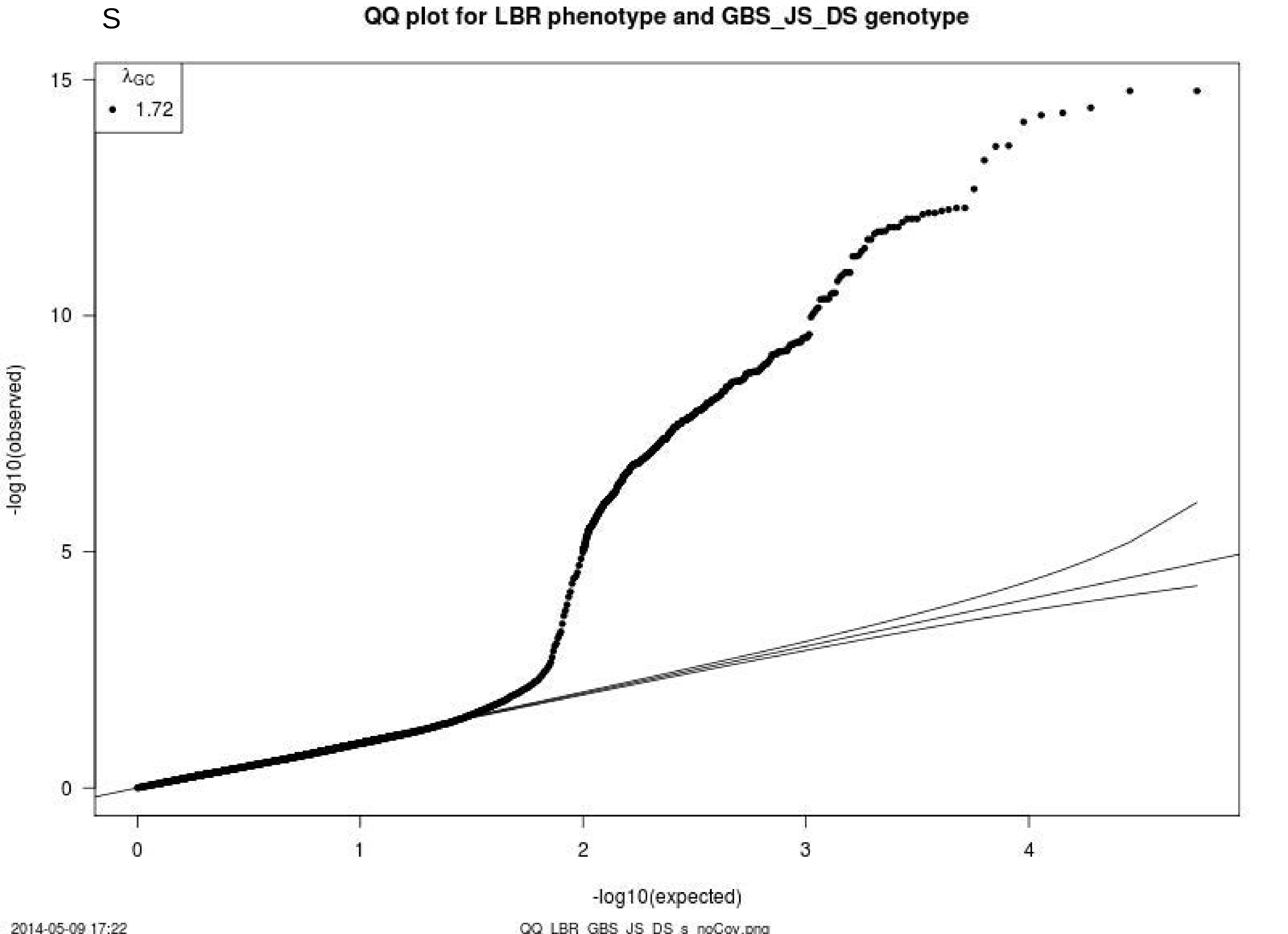
QQ plot for LBR phenotype and GBS_JS_DS genotype
λGC
1.72
15
10
5
0
-log10(observed)
0
1
2
3
4
-log10(expected)
2014-05-09 17:22
QQ_LBR_GBS_JS_DS_s_noCov.png

T

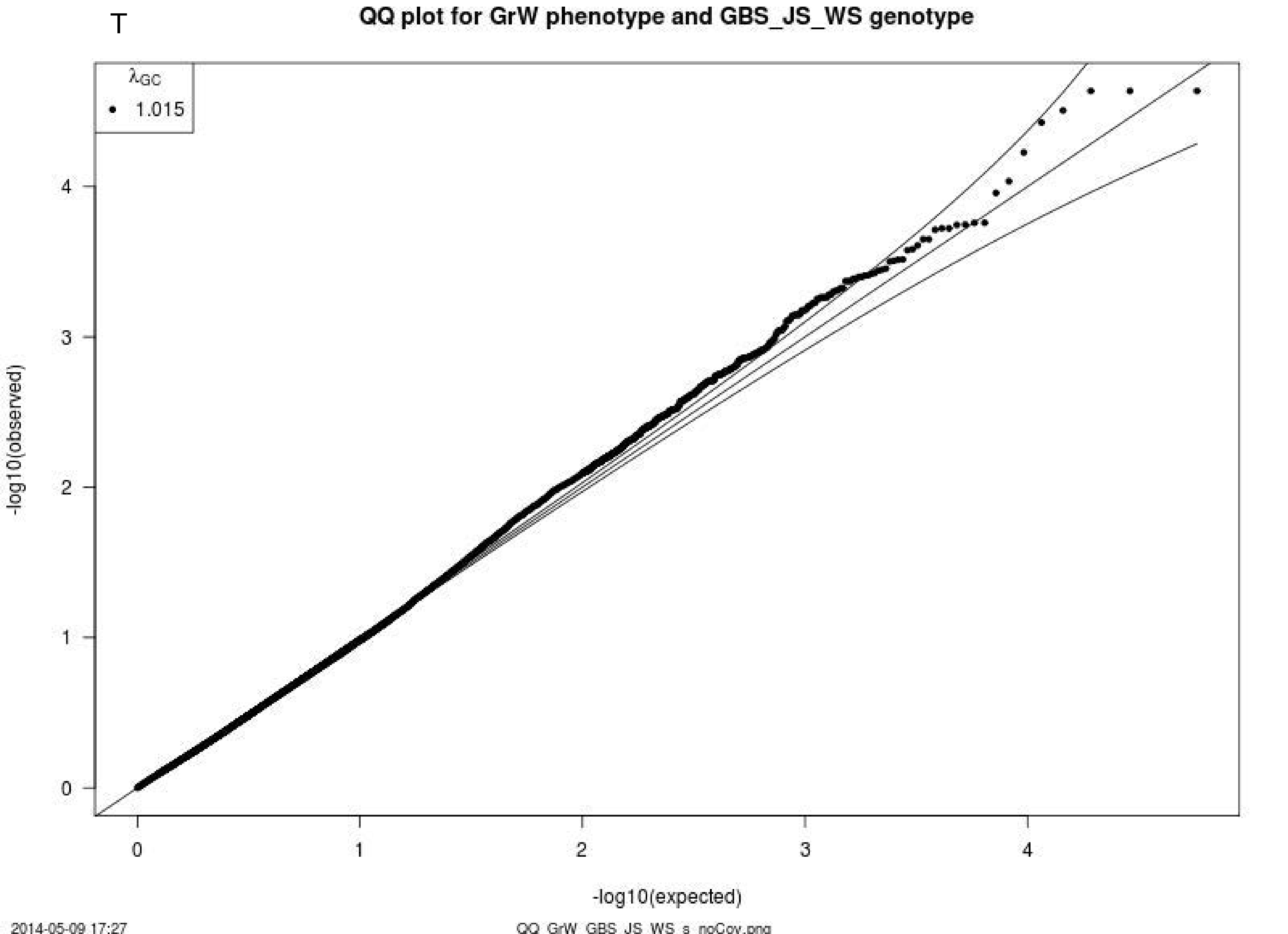
QQ plot for GrW phenotype and GBS_JS_WS genotype
λ_{GC}
1.015
-log10(observed)
-log10(expected)
0
1
2
3
4
2014-05-09 17:27
QQ_GrW_GBS_JS_WS_s_noCov.png

U

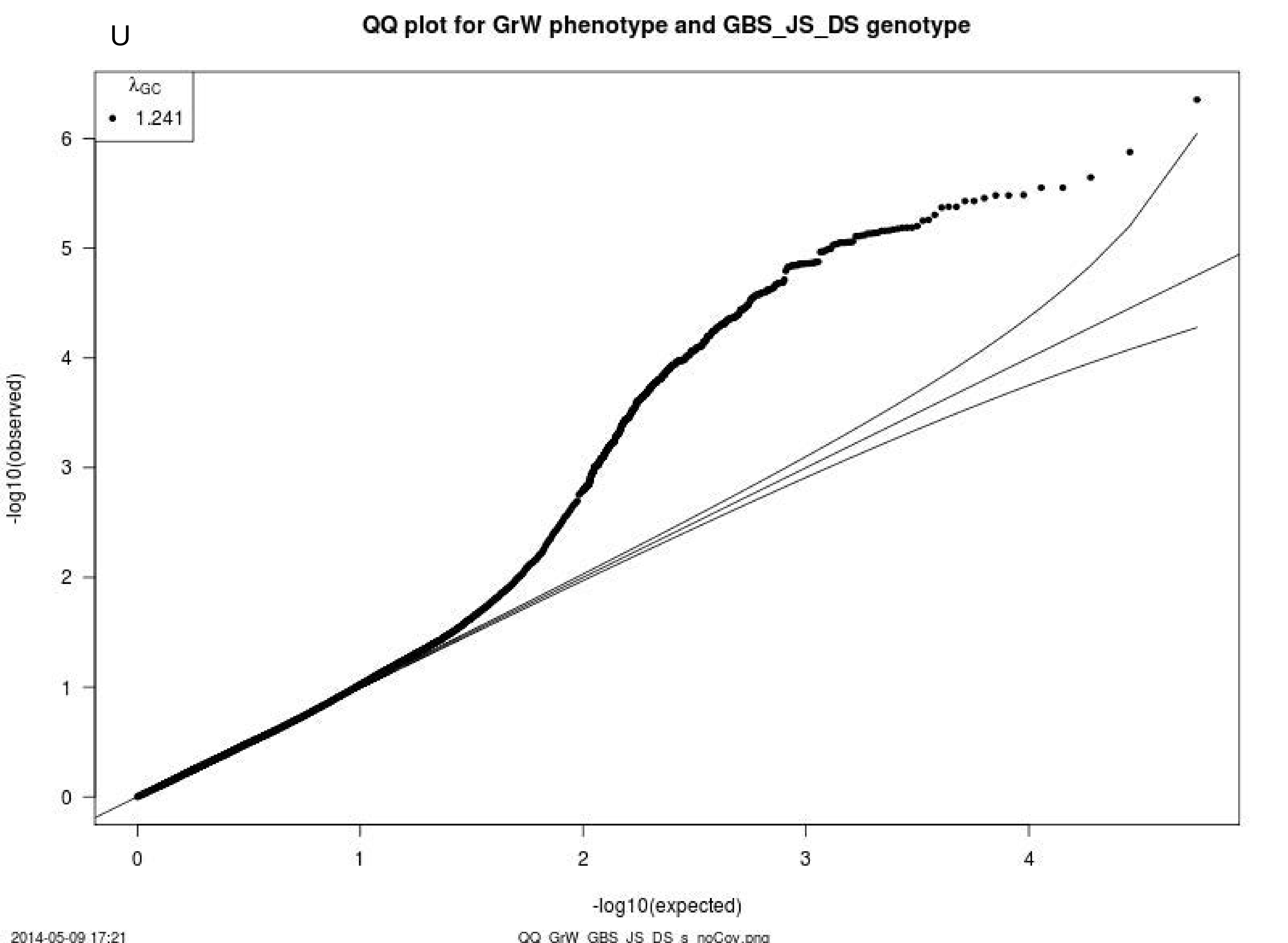
QQ plot for GrW phenotype and GBS_JS_DS genotype
λGC
1.241
-log10(observed)
-log10(expected)
0
1
2
3
4
5
6
2014-05-09 17:21
QQ_GrW_GBS_JS_DS_s_noCov.png

V

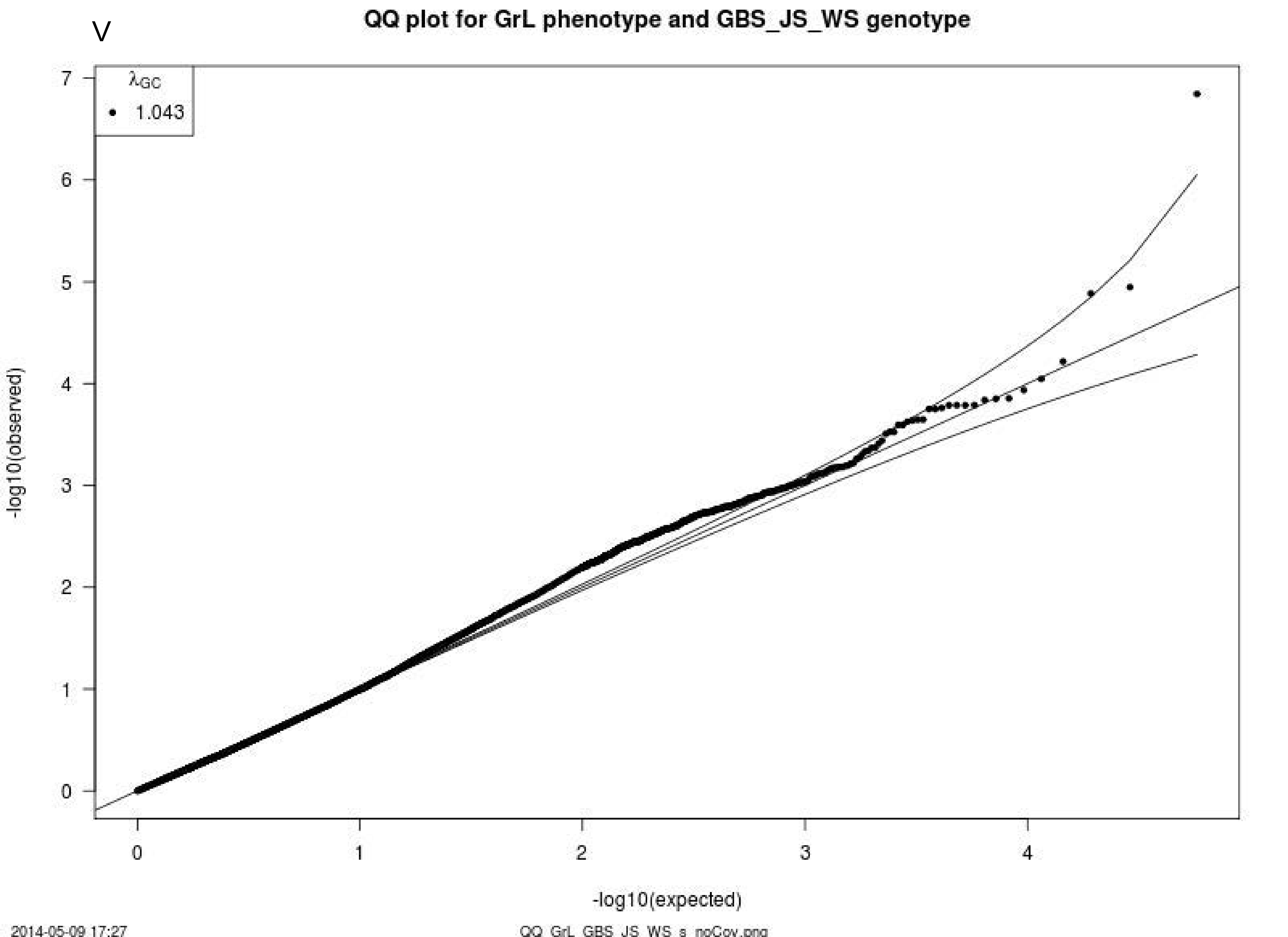
QQ plot for GrL phenotype and GBS_JS_WS genotype
λGC
1.043
-log10(observed)
-log10(expected)
0
1
2
3
4
5
6
7
2014-05-09 17:27
QQ_GrL_GBS_JS_WS_s_noCov.png

W

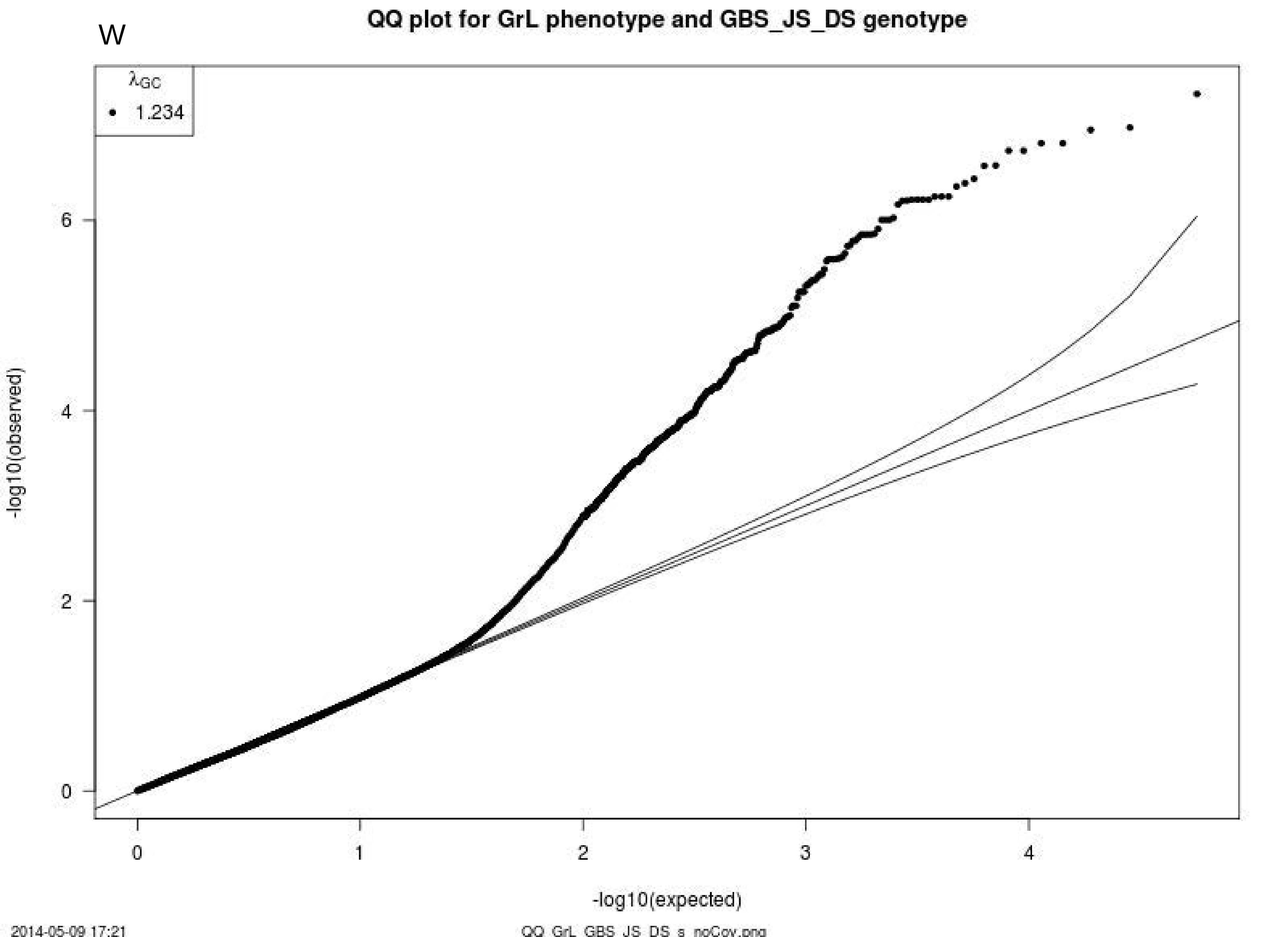
QQ plot for GrL phenotype and GBS_JS_DS genotype
λGC
1.234
6
4
2
0
-log10(observed)
0
1
2
3
4
-log10(expected)
2014-05-09 17:21
QQ_GrL_GBS_JS_DS_s_noCov.png

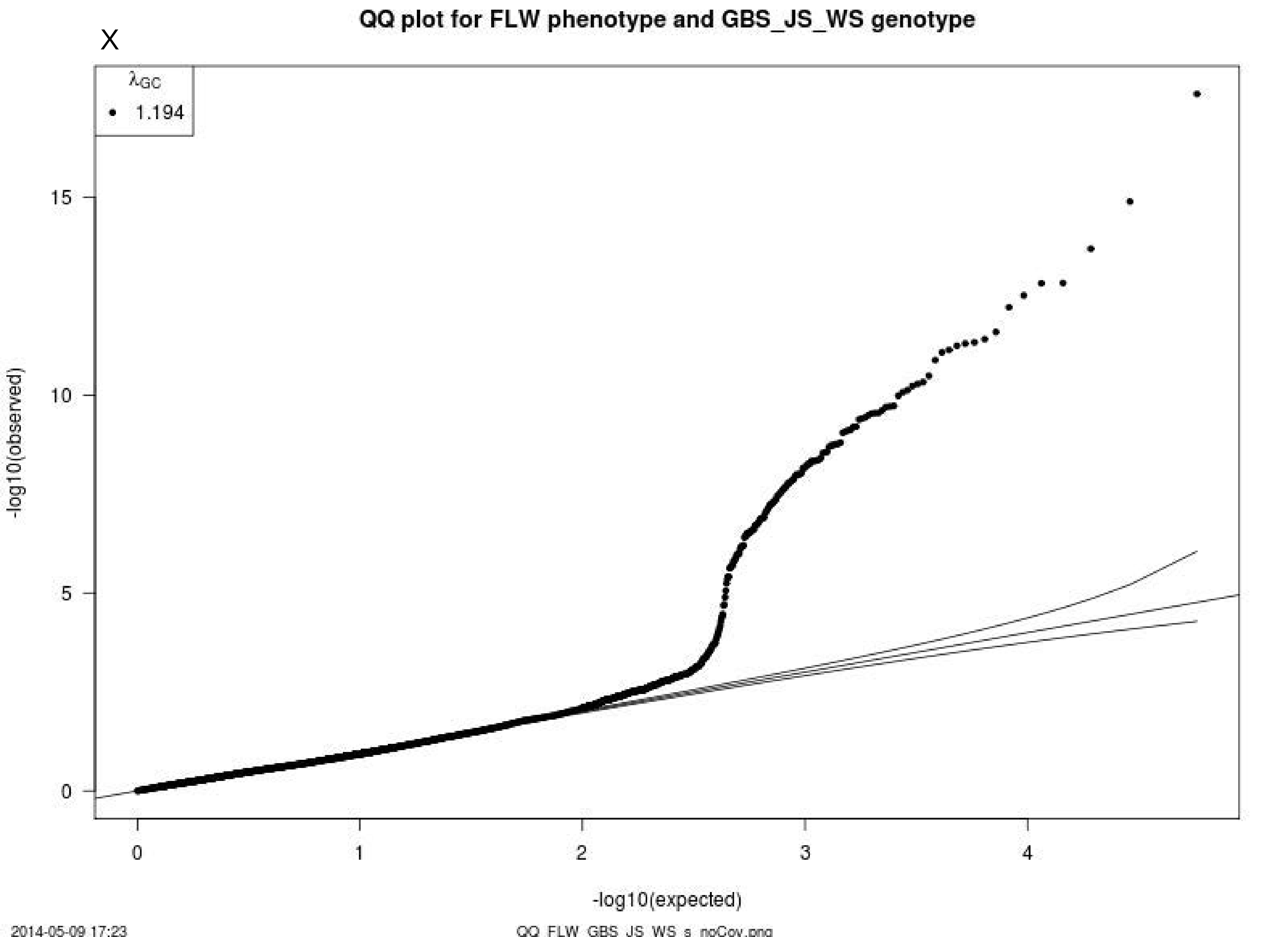
QQ plot for FLW phenotype and GBS_JS_WS genotype
X
λ_{GC}
1.194
15
10
5
0
-log10(observed)
0
1
2
3
4
-log10(expected)
2014-05-09 17:23
QQ_FLW_GBS_JS_WS_s_noCov.png

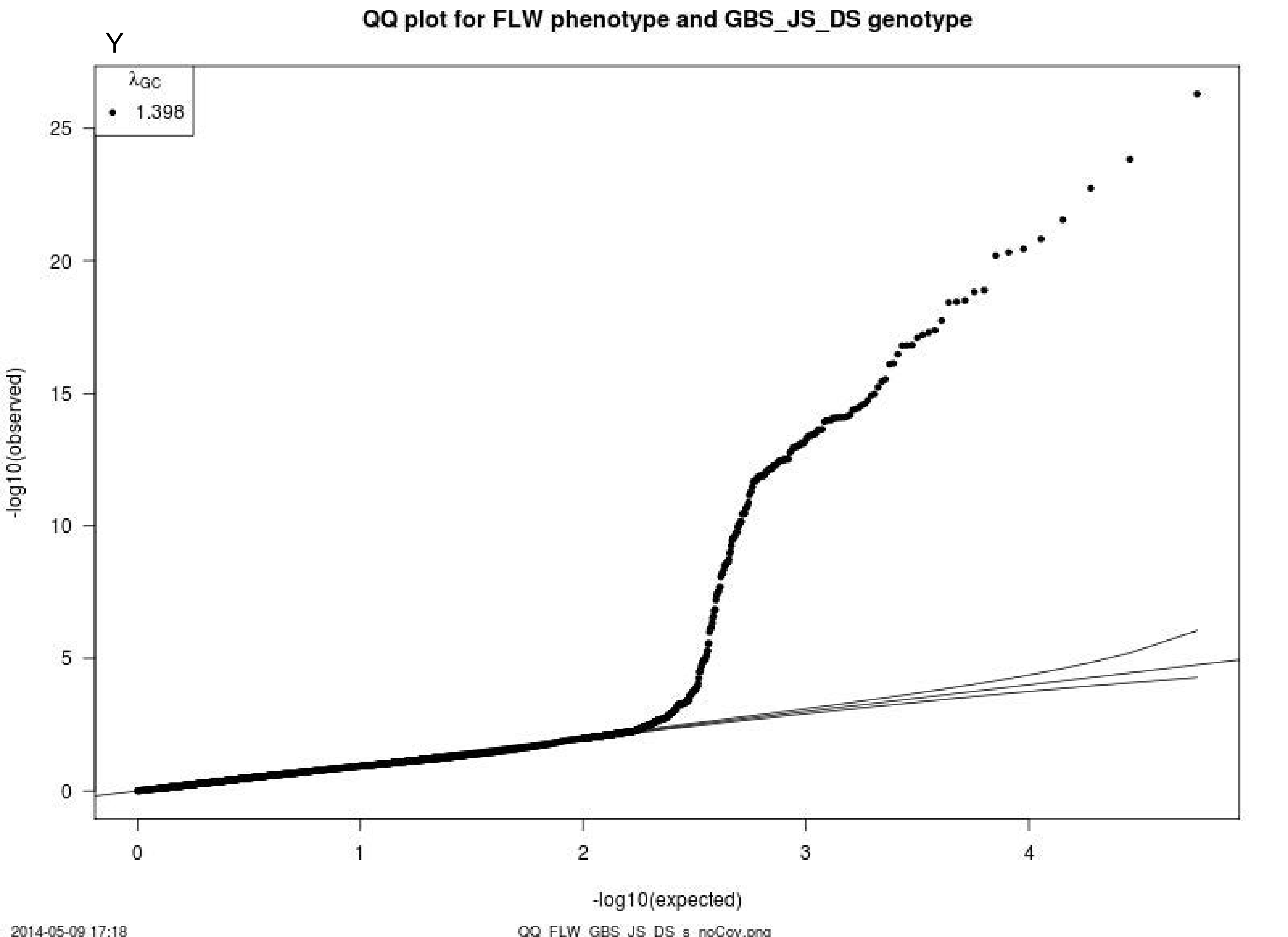

QQ plot for FLW phenotype and GBS_JS_DS genotype
Y
λGC
1.398
-log10(observed)
-log10(expected)
0
5
10
15
20
25
0
1
2
3
4
2014-05-09 17:18
QQ_FLW_GBS_JS_DS_s_noCov.png

Z

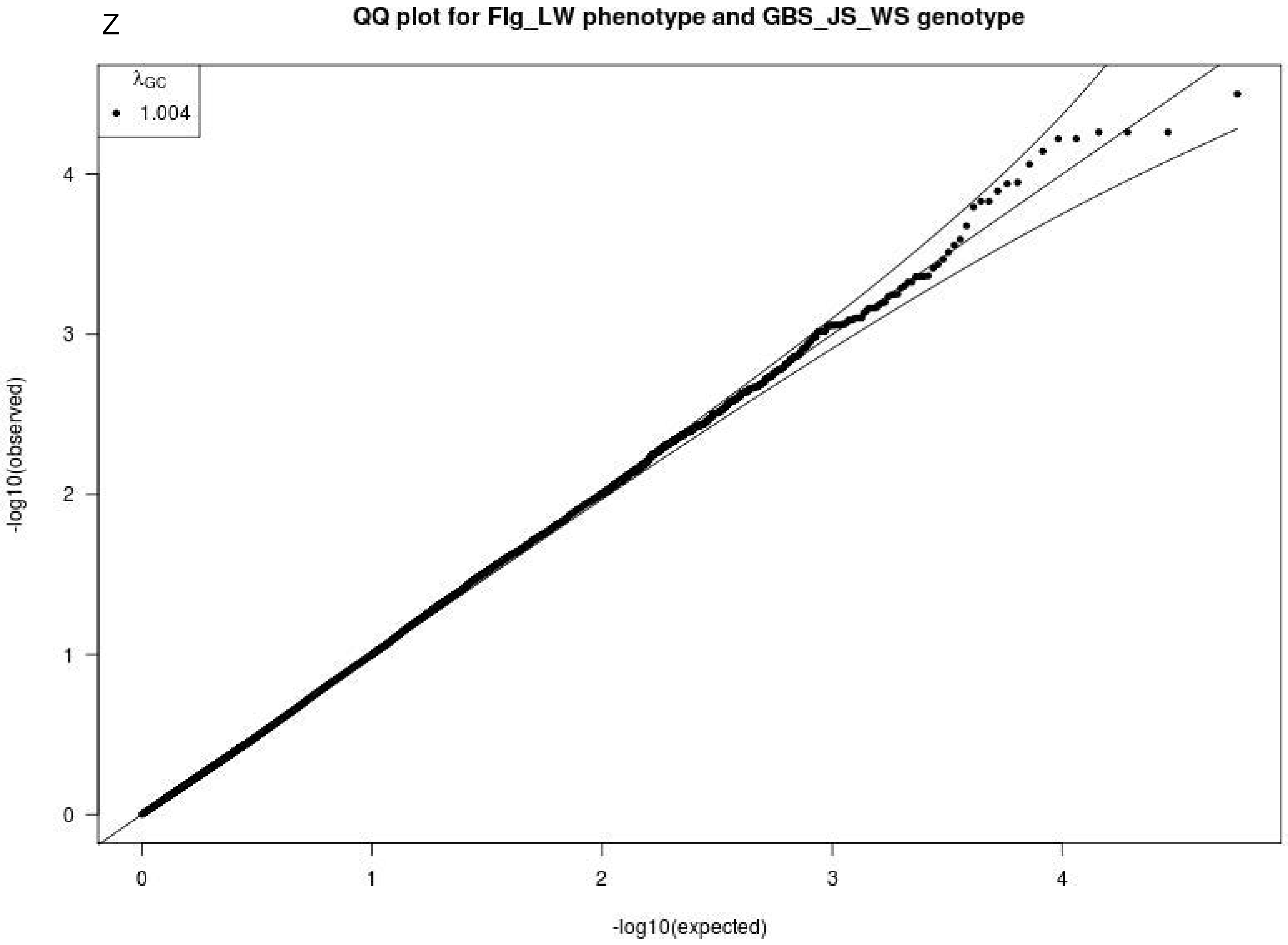
QQ plot for Flg_LW phenotype and GBS_JS_WS genotype
λ_{GC}
1.004
-log10(observed)
-log10(expected)
0
1
2
3
4

AA

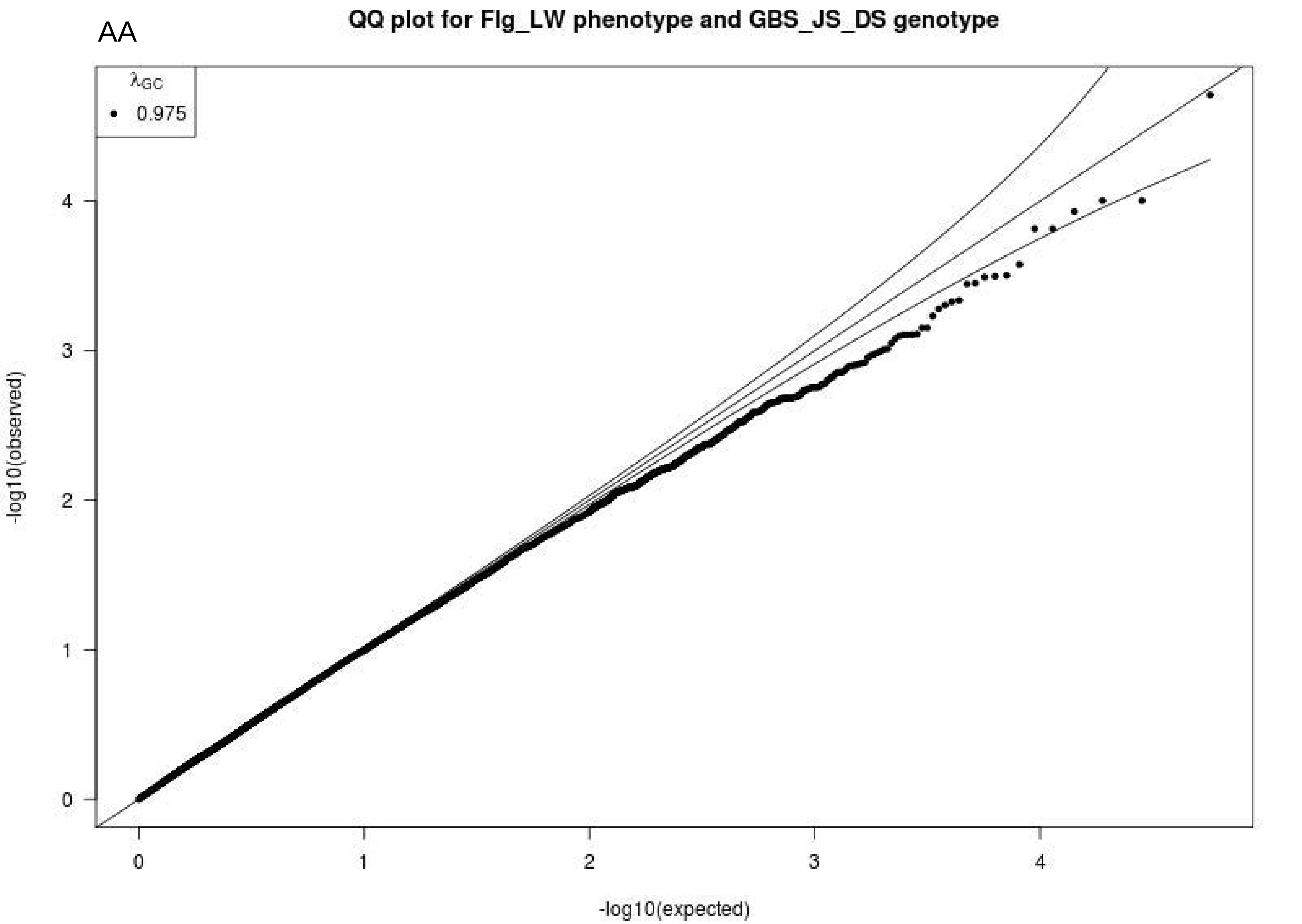

2014-05-09 17:20 QQ_Flg_LW_GBS_JS_DS_s_noCov.png

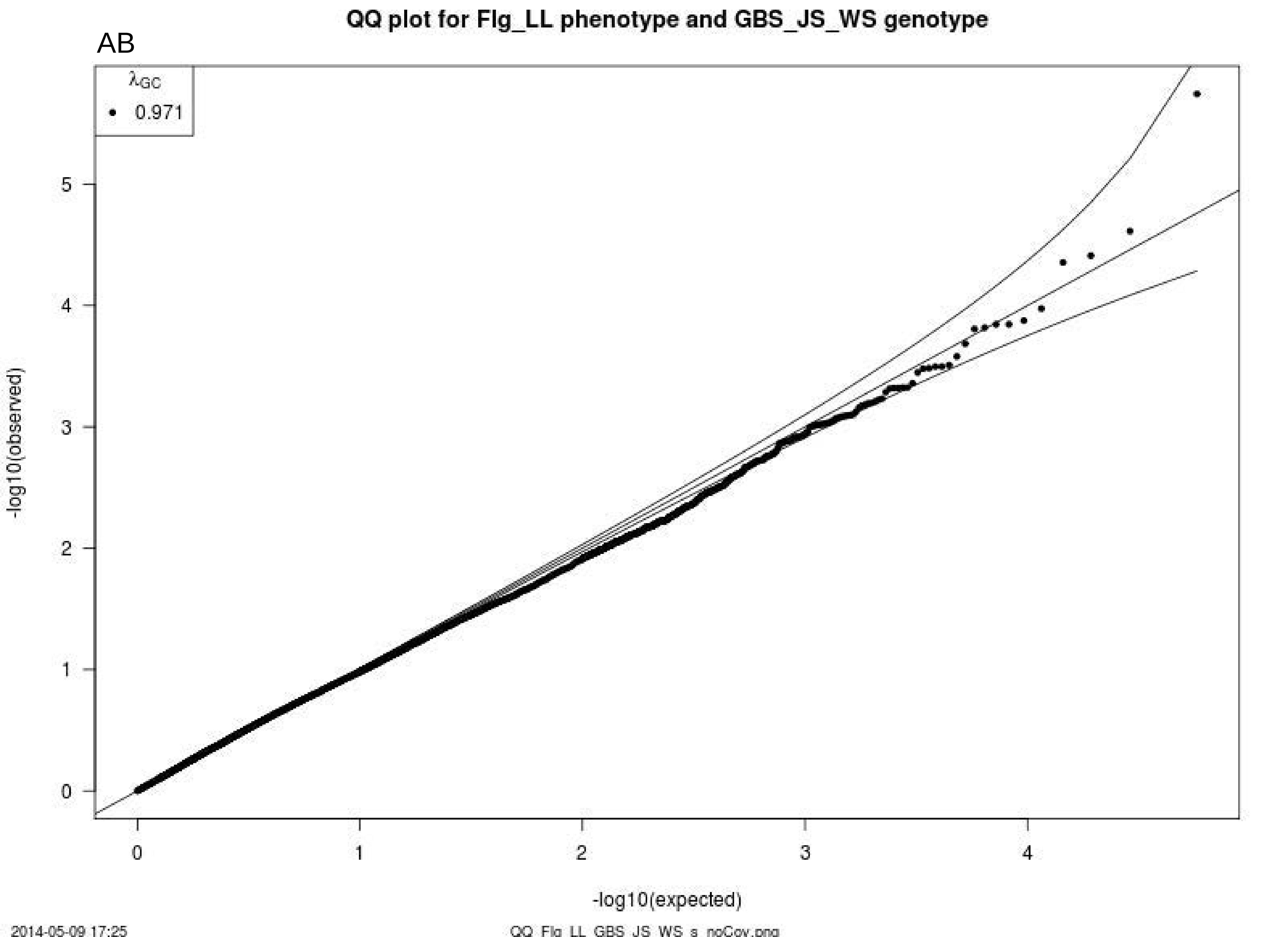

QQ plot for Flg_LL phenotype and GBS_JS_WS genotype
AB
λGC
0.971
5
4
3
2
1
0
-log10(observed)
0
1
2
3
4
-log10(expected)
2014-05-09 17:25
QQ_Flg_LL_GBS_JS_WS_s_noCov.png

AC

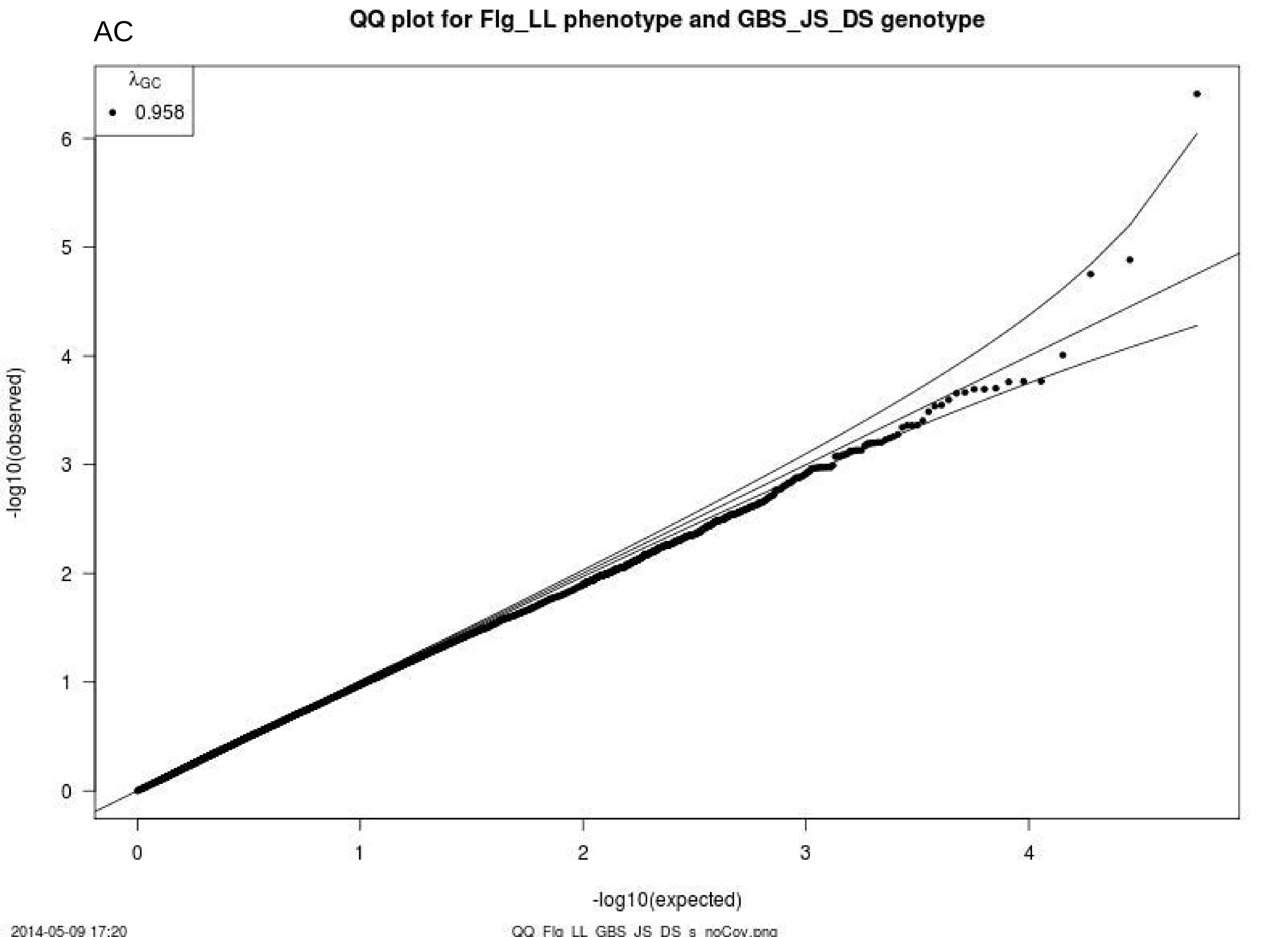
QQ plot for Flg_LL phenotype and GBS_JS_DS genotype
λGC
0.958
-log10(observed)
-log10(expected)
0
1
2
3
4
5
6
0
1
2
3
4
2014-05-09 17:20
QQ_Flg_LL_GBS_JS_DS_s_noCov.png

AD

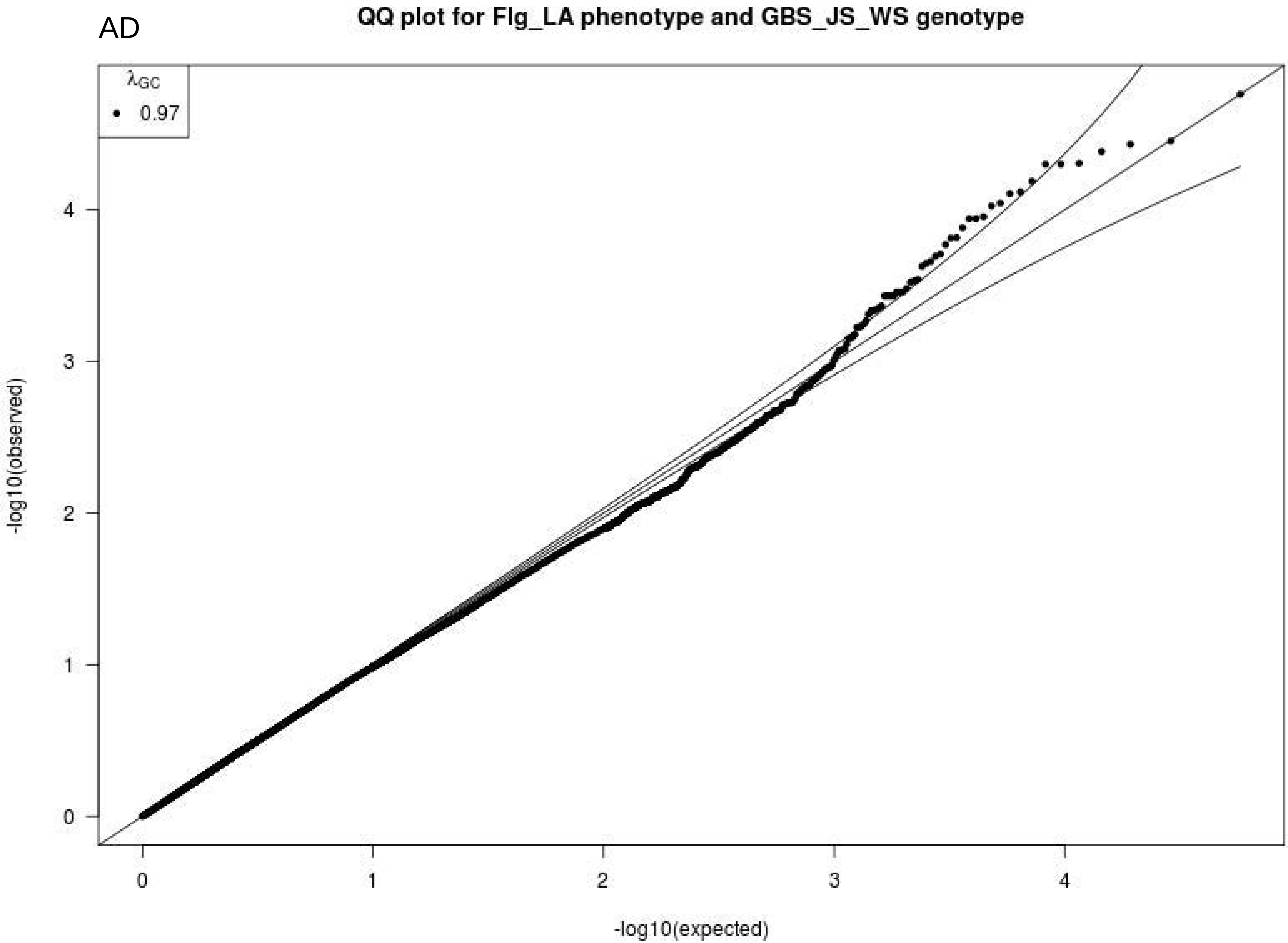

2014-05-09 17:26 QQ_Flg_LA_GBS_JS_WS_s_noCov.png

AE

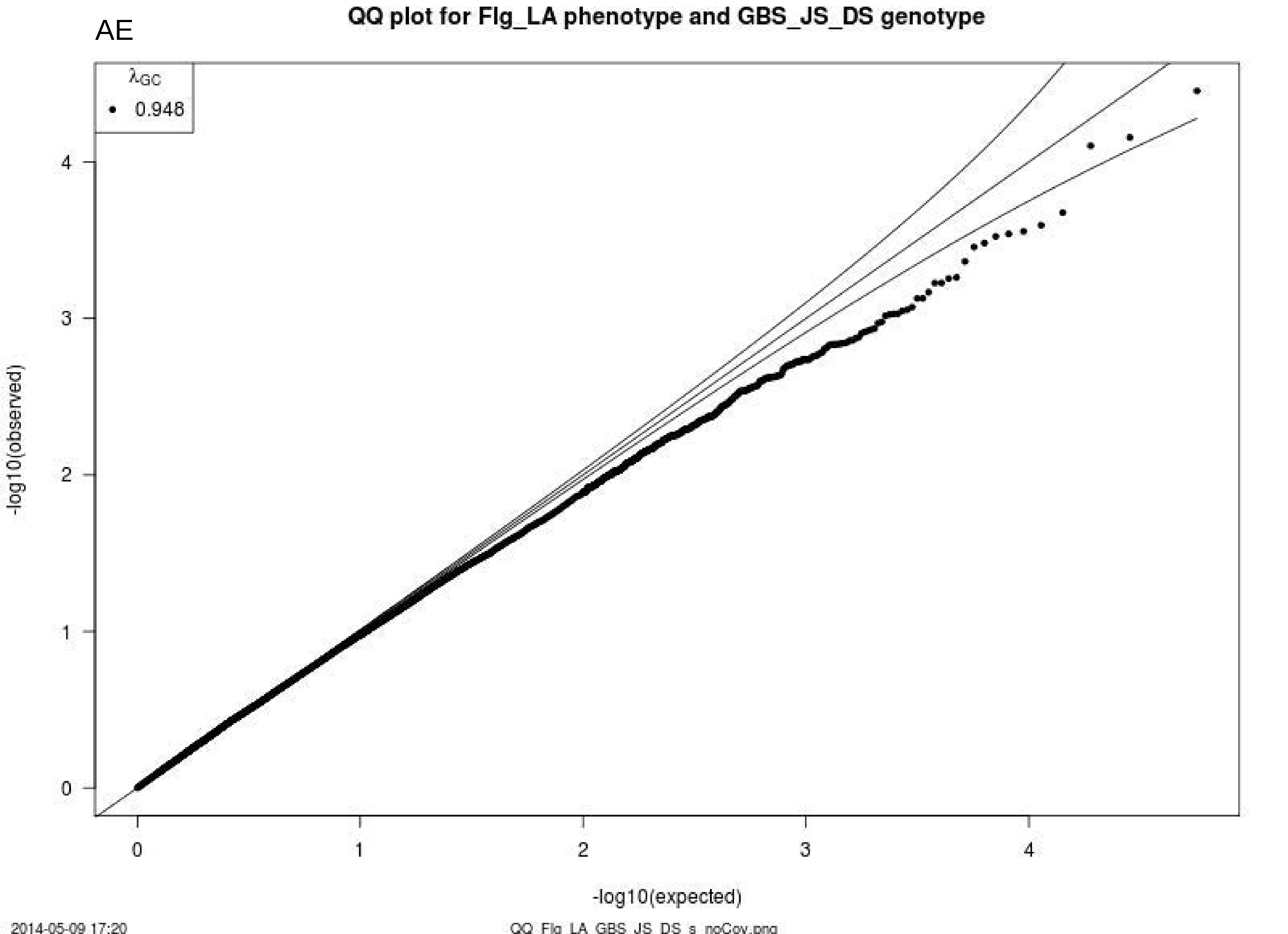
QQ plot for Flg_LA phenotype and GBS_JS_DS genotype
λ_{GC}
0.948
-log10(observed)
-log10(expected)
0
1
2
3
4
2014-05-09 17:20
QQ_Flg_LA_GBS_JS_DS_s_noCov.png

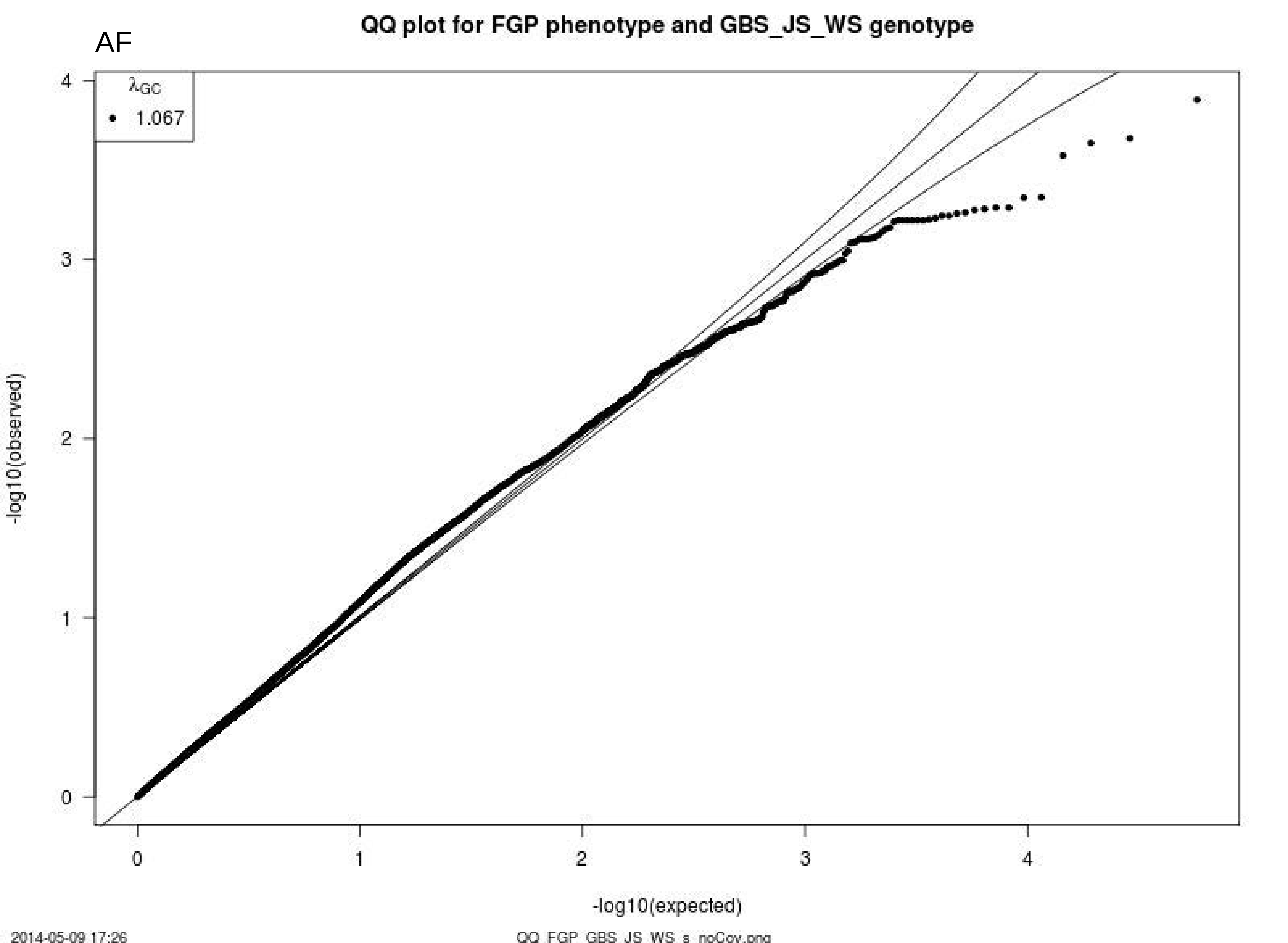

AF
QQ plot for FGP phenotype and GBS_JS_WS genotype
λ_{GC}
1.067
-log10(observed)
-log10(expected)
0
1
2
3
4
2014-05-09 17:26
QQ_FGP_GBS_JS_WS_s_noCov.png

AG

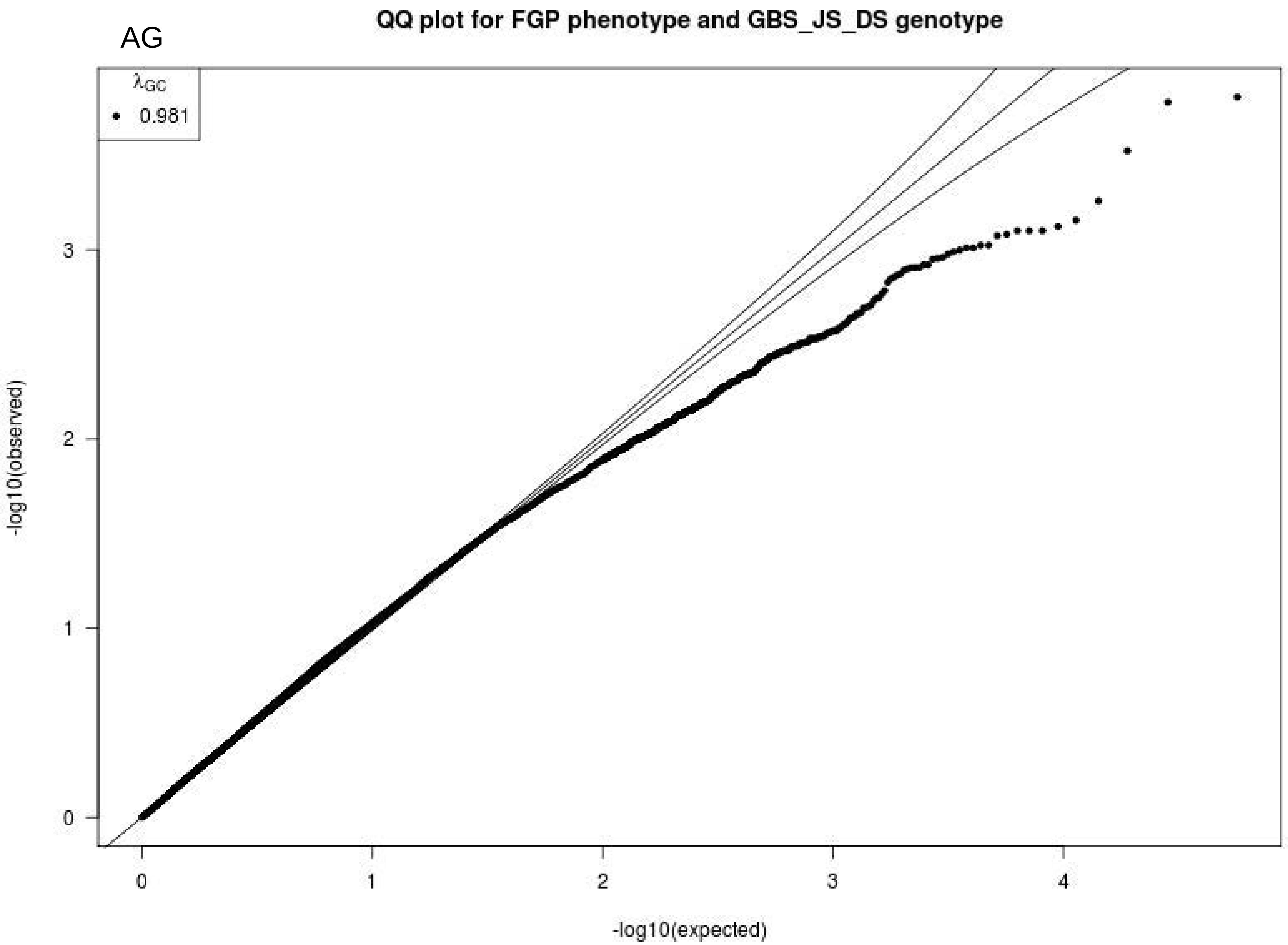

2014-05-09 17:21 QQ_FGP_GBS_JS_DS_s_noCov.png

AH

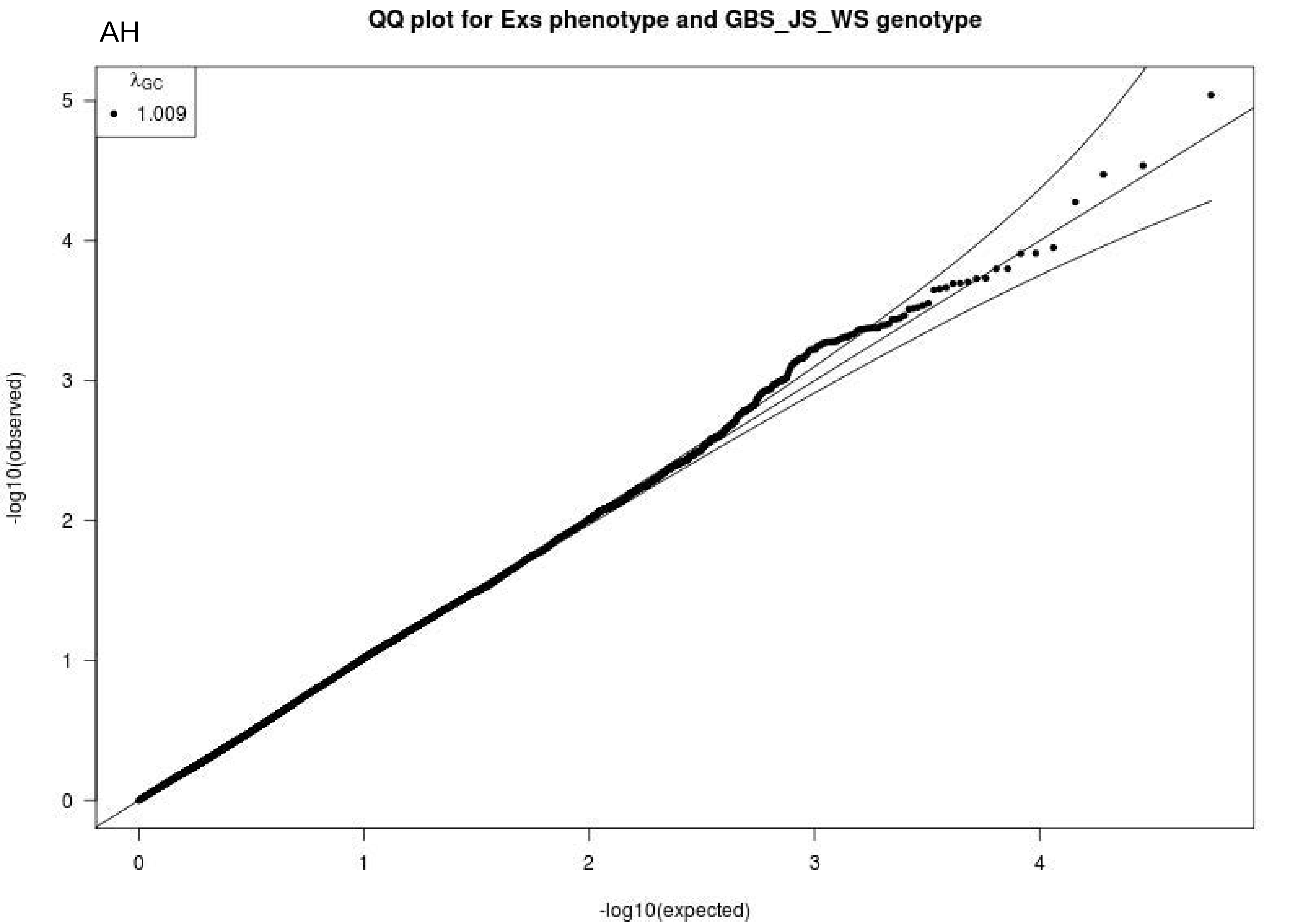

2014-05-09 17:24

QQ_Exs_GBS_JS_WS_s_noCov.png

AI

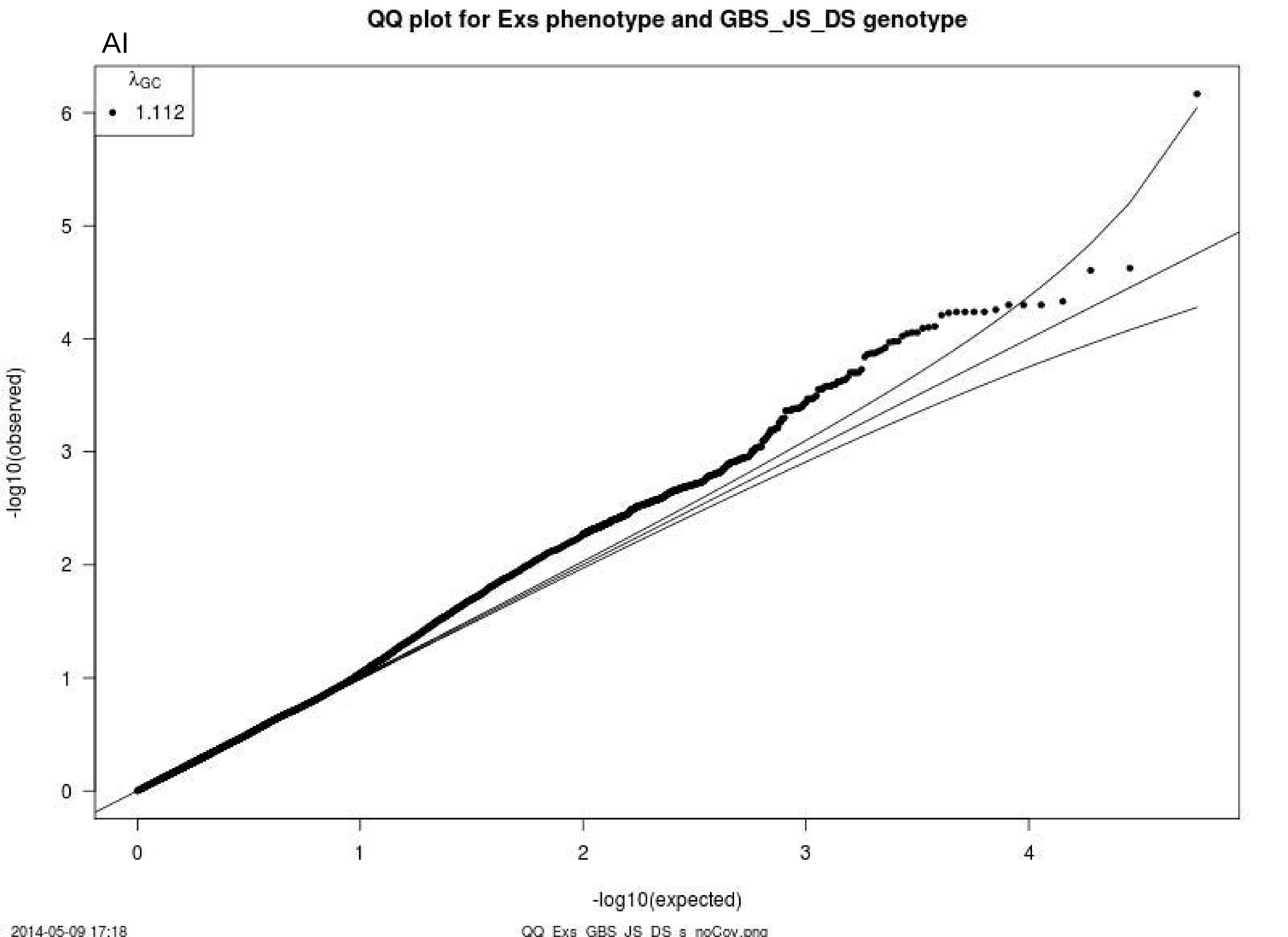
QQ plot for Exs phenotype and GBS_JS_DS genotype
λGC
1.112
-log10(observed)
-log10(expected)
0
1
2
3
4
5
6
0
1
2
3
4
2014-05-09 17:18
QQ_Exs_GBS_JS_DS_s_noCov.png

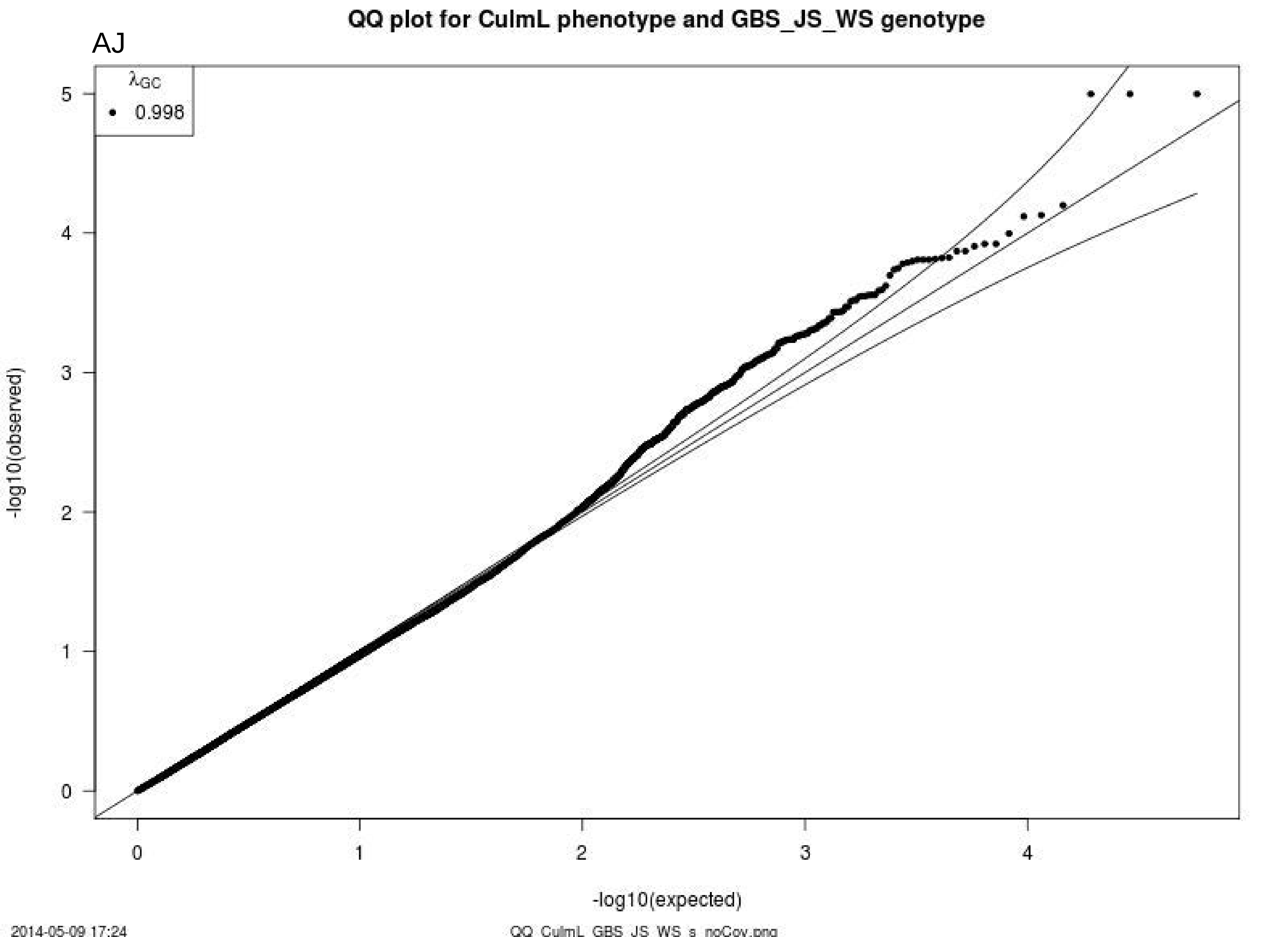
QQ plot for CulmL phenotype and GBS_JS_WS genotype
AJ
λGC
0.998
-log10(observed)
-log10(expected)
0
1
2
3
4
5
2014-05-09 17:24
QQ_CulmL_GBS_JS_WS_s_noCov.png

AK

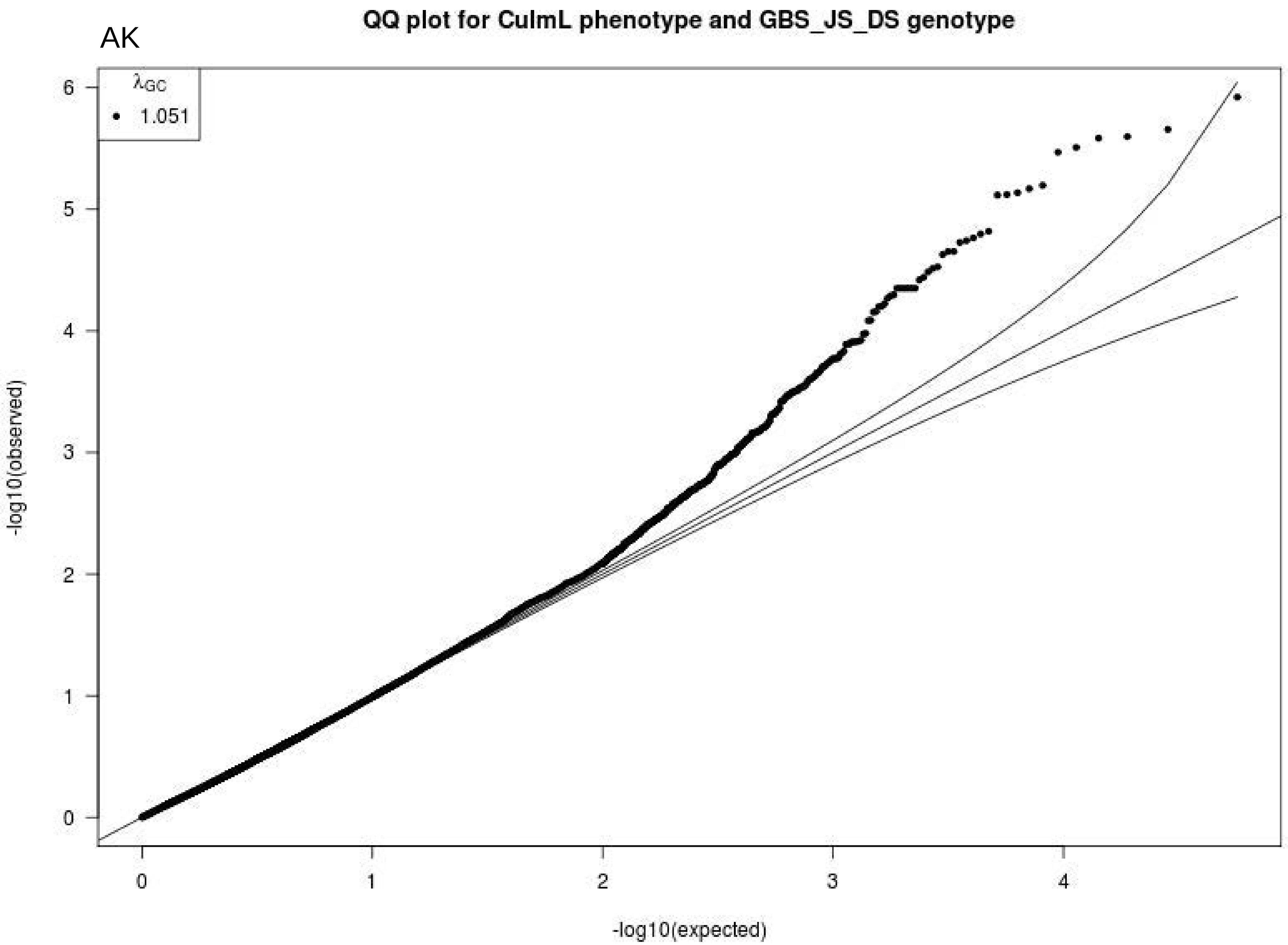

2014-05-09 17:18 QQ_CulmL_GBS_JS_DS_s_noCov.png

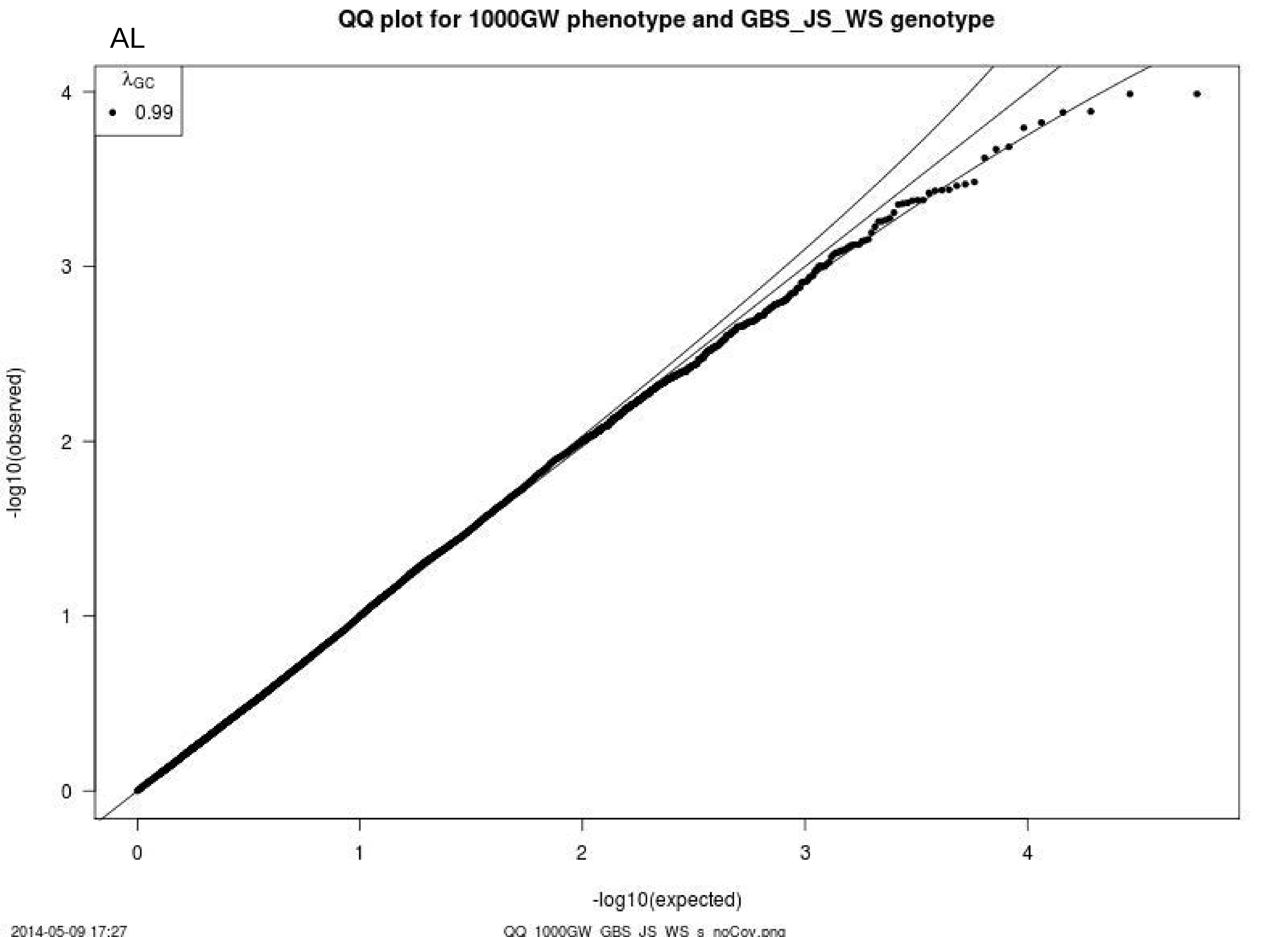
QQ plot for 1000GW phenotype and GBS_JS_WS genotype
AL
λGC
0.99
-log10(observed)
-log10(expected)
0
1
2
3
4
2014-05-09 17:27
QQ_1000GW_GBS_JS_WS_s_noCov.png
