# Supplementary material for: Genome-Wide Association Mapping for Yield and Other Agronomic Traits in an Elite Breeding Population of Tropical Rice (Oryza sativa)
Source: PLoS One. 2015 Mar 18;10(3):e0119873. doi: 10.1371/journal.pone.0119873 (PMC4364887; doi:10.1371/journal.pone.0119873)
Supplement: S3 Fig — Dashed line shows the 0.1 FDR significance threshold. Plots with no dashed line did not have any SNPs that passed the significance threshold. (PDF) [file pone.0119873.s003.pdf]

A

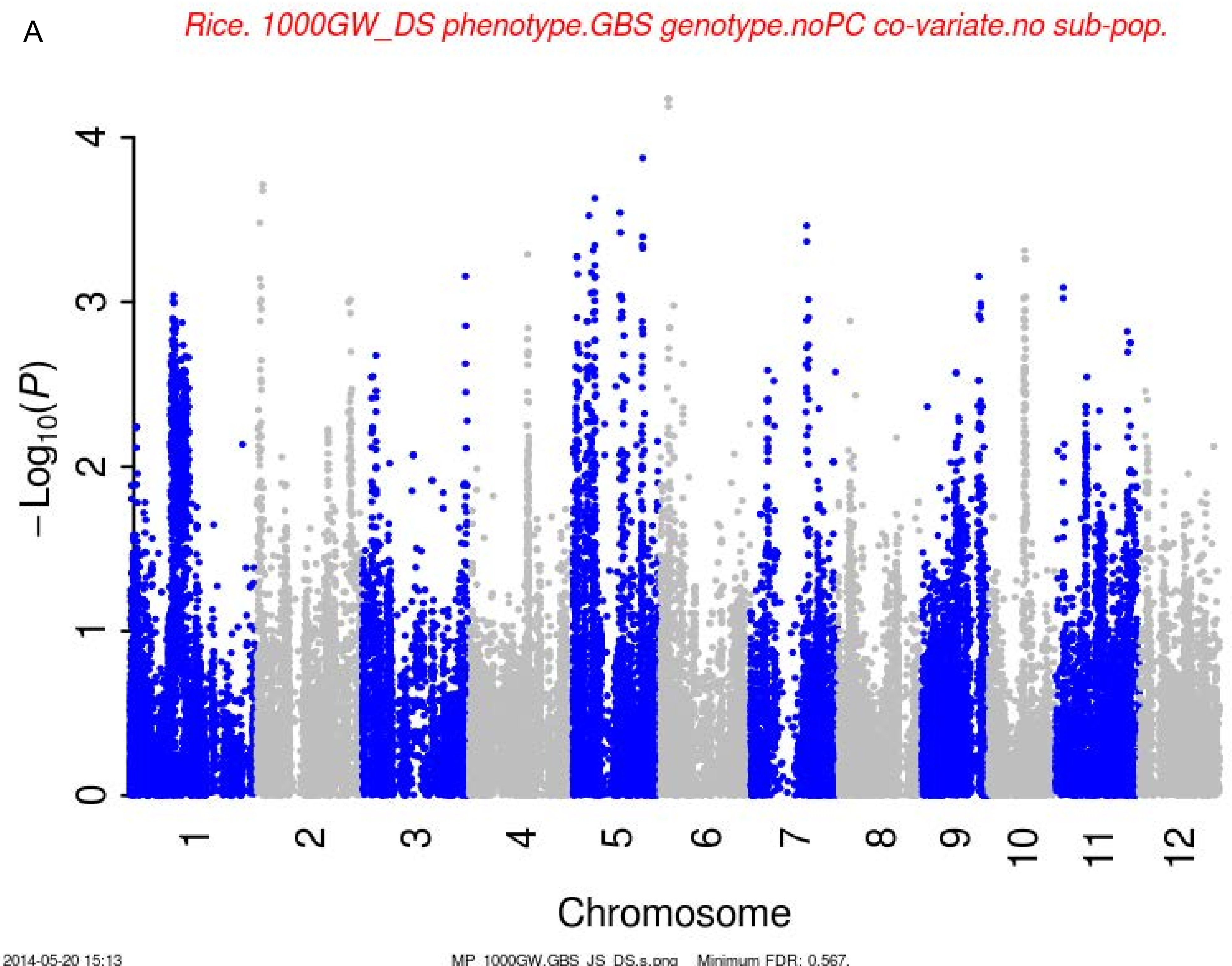
Rice. 1000GW_DS phenotype.GBS genotype.noPC co-variate.no sub-pop.
-Log10(P)
0
1
2
3
4
1
2
3
4
5
6
7
8
9
10
11
12
Chromosome
2014-05-20 15:13
MP_1000GW.GBS_JS_DS.s.png
Minimum FDR: 0.567.

B

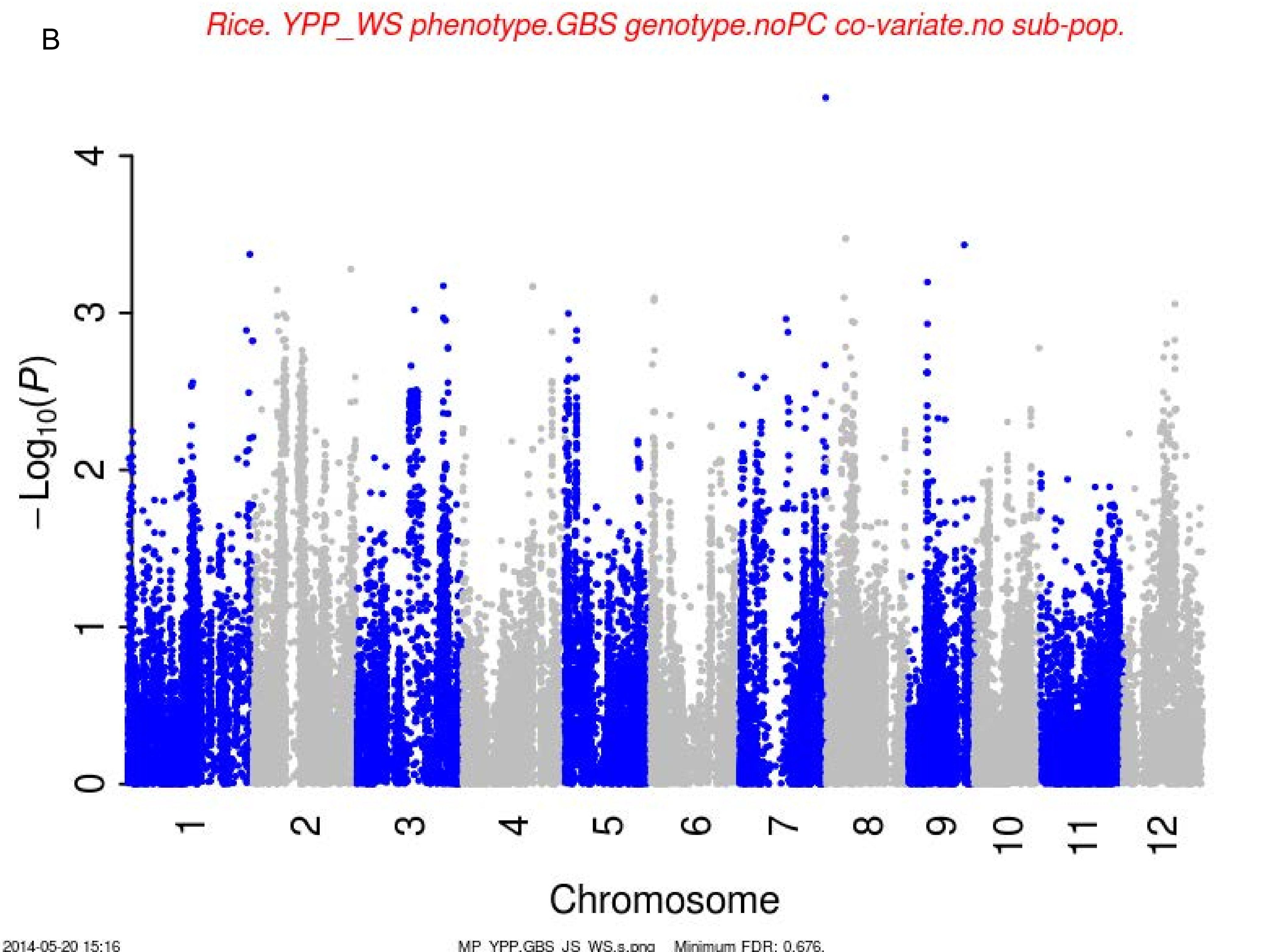
Rice. YPP_WS phenotype.GBS genotype.noPC co-variate.no sub-pop.
$-\mathrm{Log}_{10}(P)$
0
1
2
3
4
1
2
3
4
5
6
7
8
9
10
11
12
Chromosome
2014-05-20 15:16
MP_YPP.GBS_JS_WS.s.png
Minimum FDR: 0.676.

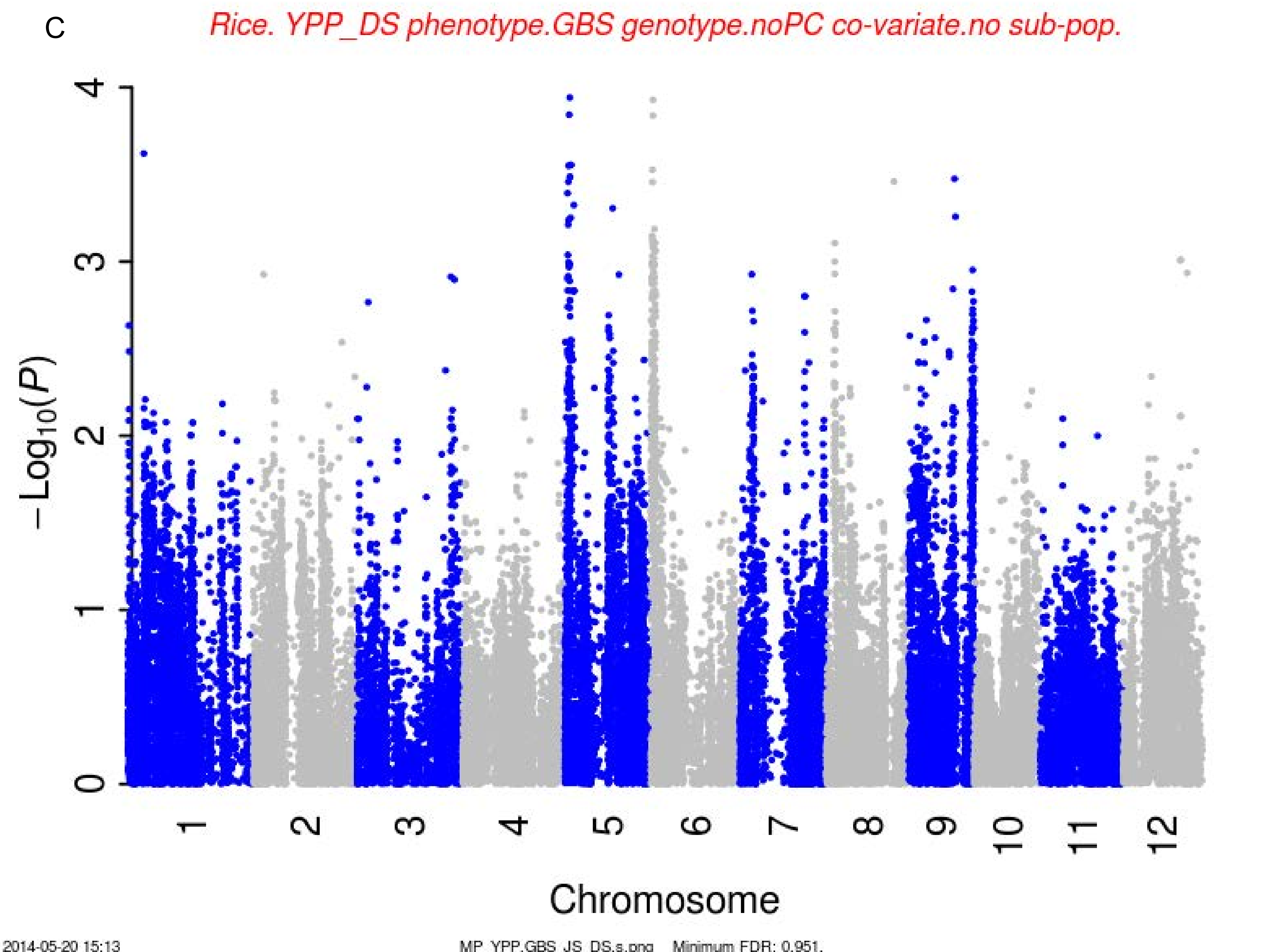
C
Rice. YPP_DS phenotype.GBS genotype.noPC co-variate.no sub-pop.
$-\text{Log}_{10}(P)$
0
1
2
3
4
1 2 3 4 5 6 7 8 9 10 11 12
Chromosome
2014-05-20 15:13
MP_YPP.GBS_JS_DS.s.png Minimum FDR: 0.951.

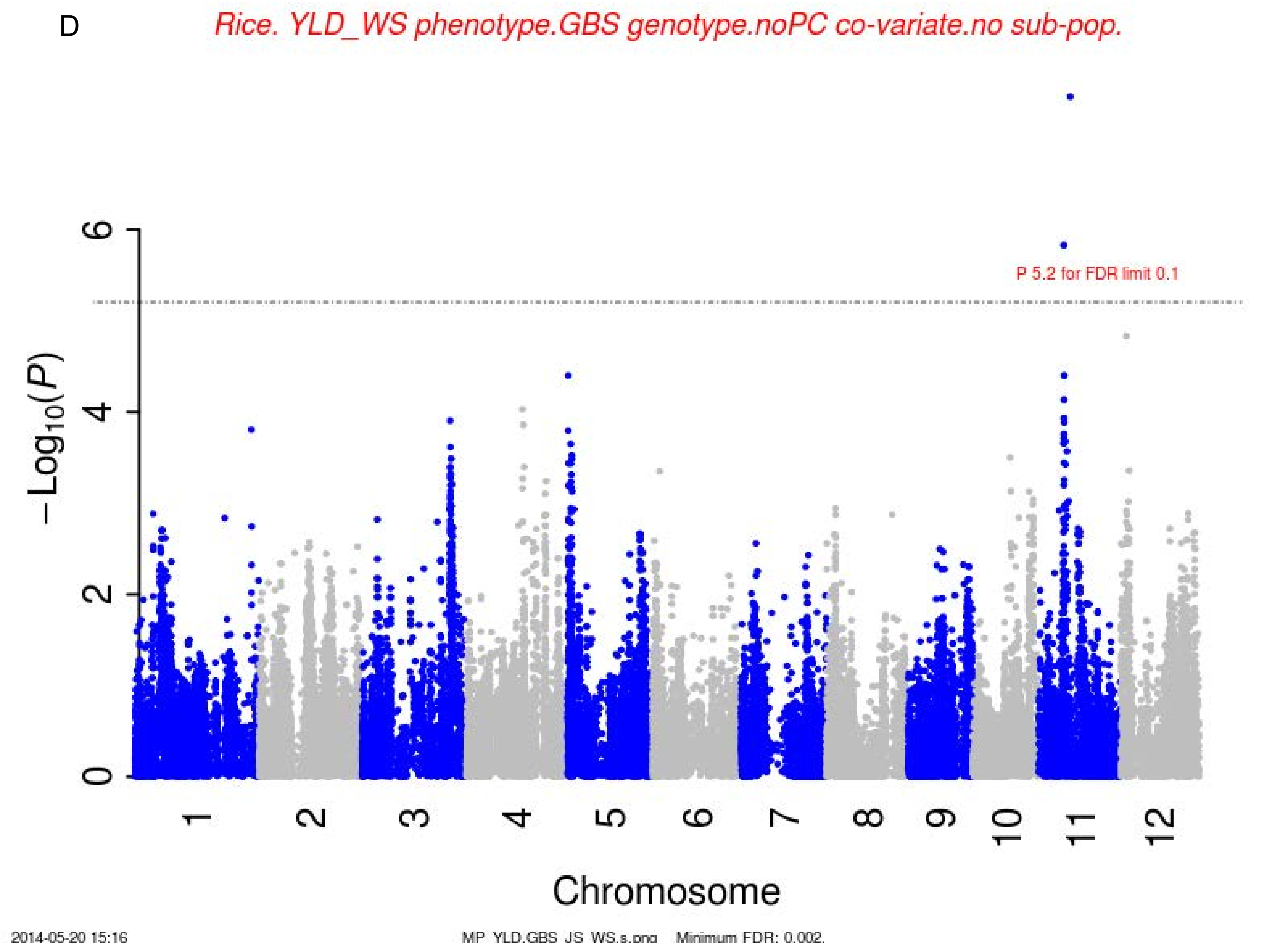
D
Rice. YLD_WS phenotype.GBS genotype.noPC co-variate.no sub-pop.
P 5.2 for FDR limit 0.1
−Log10(P)
0
2
4
6
1
2
3
4
5
6
7
8
9
10
11
12
Chromosome
2014-05-20 15:16
MP_YLD.GBS_JS_WS.s.png Minimum FDR: 0.002.

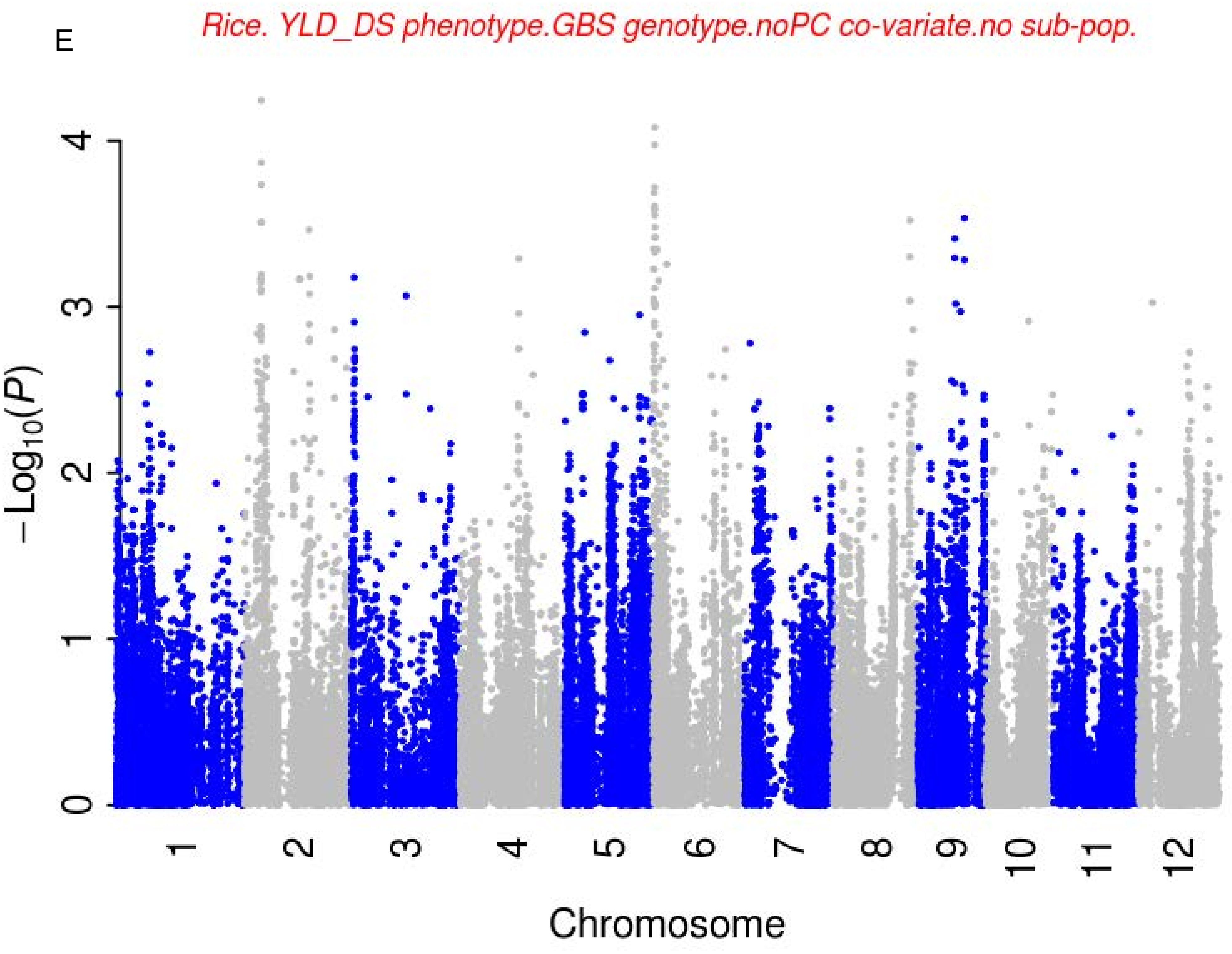

2014-05-20 15:13 MP_YLD.GBS_JS_DS.s.png Minimum FDR: 0.965.

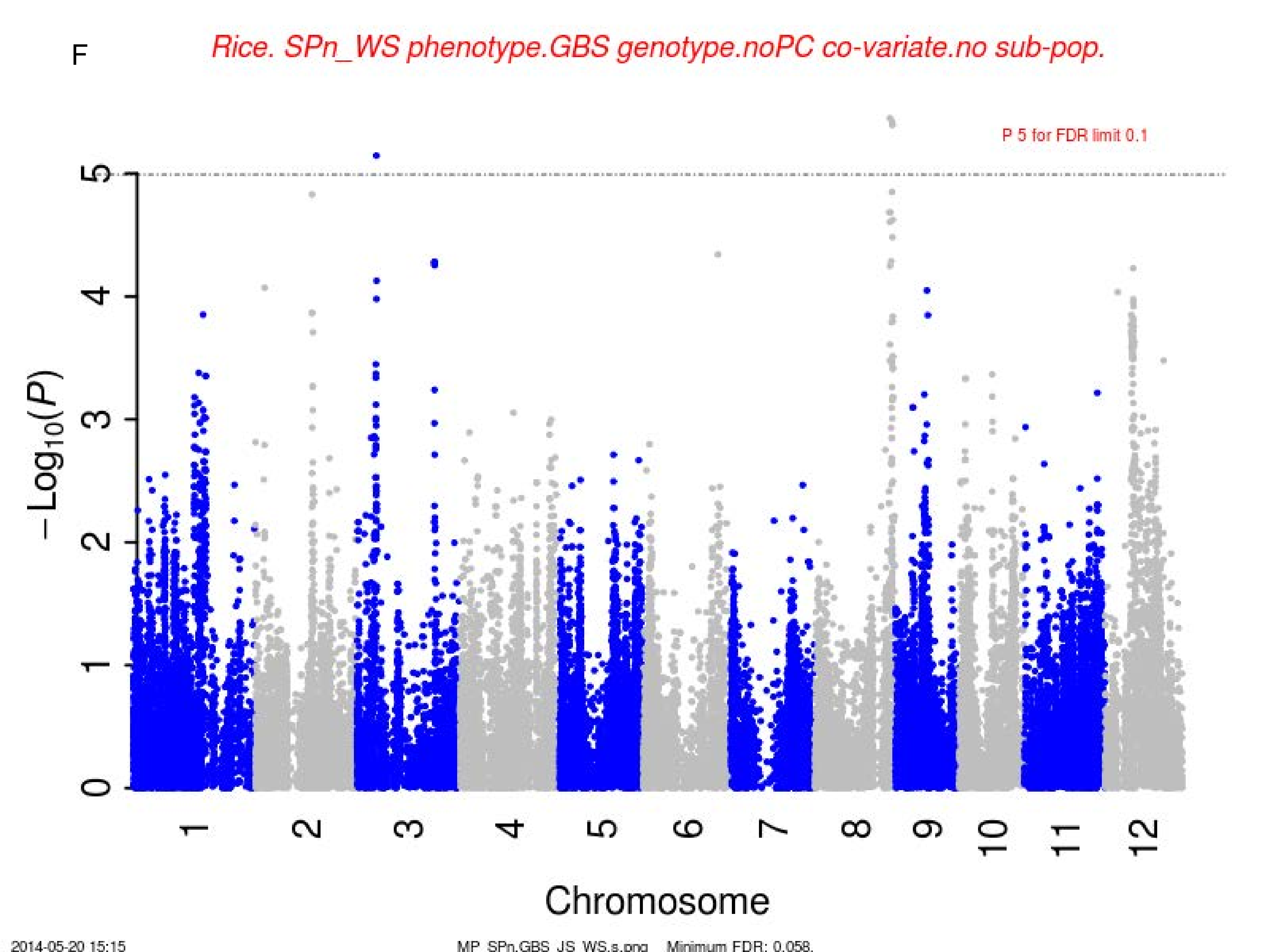
F
Rice. SPn_WS phenotype.GBS genotype.noPC co-variate.no sub-pop.
P 5 for FDR limit 0.1
−Log10(P)
0
1
2
3
4
5
1
2
3
4
5
6
7
8
9
10
11
12
Chromosome
2014-05-20 15:15
MP_SPn.GBS_JS_WS.s.png Minimum FDR: 0.058.

G

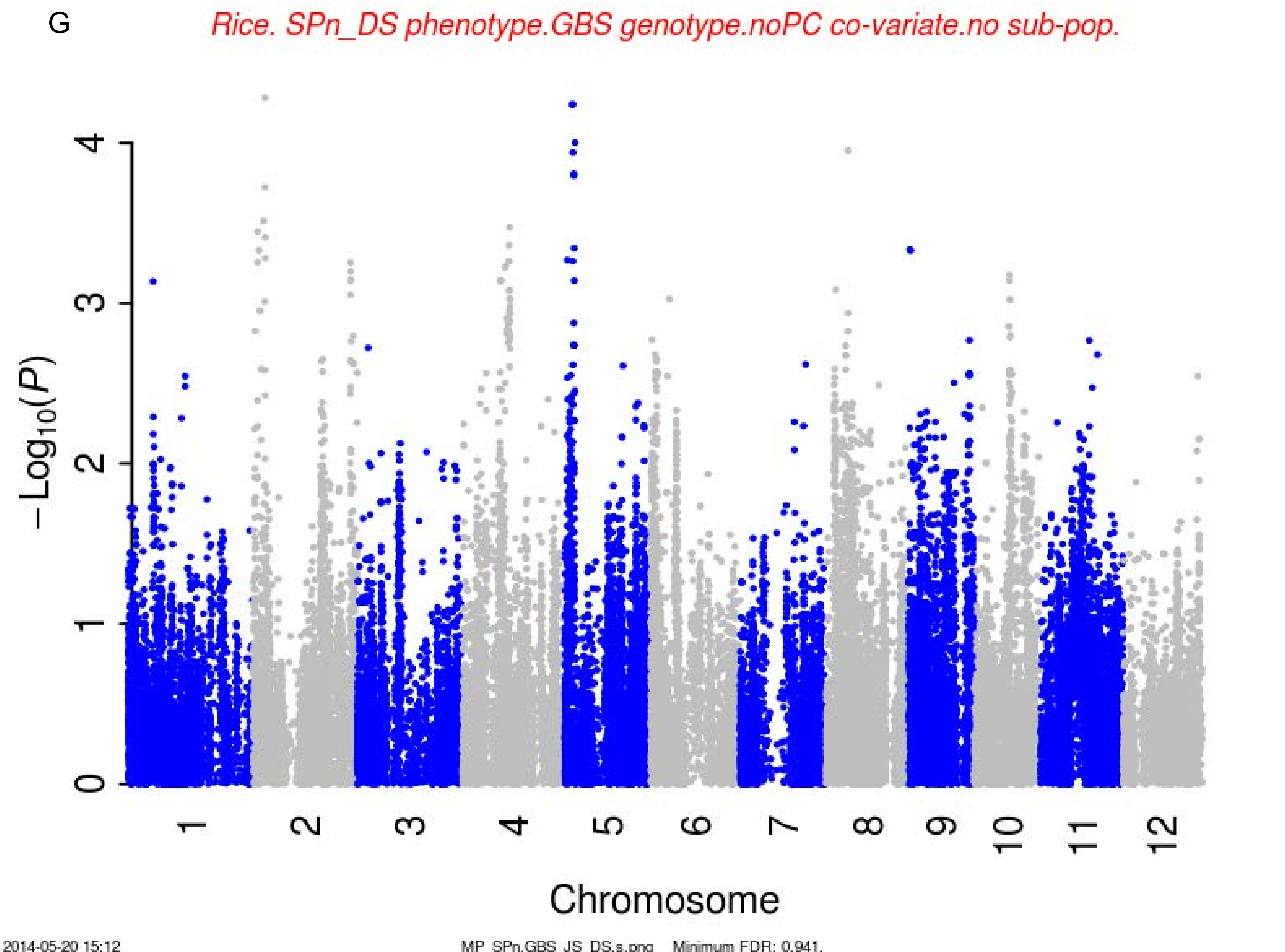
Rice. SPn_DS phenotype.GBS genotype.noPC co-variate.no sub-pop.
−Log10(P)
0
1
2
3
4
1
2
3
4
5
6
7
8
9
10
11
12
Chromosome
2014-05-20 15:12
MP_SPn.GBS_JS_DS.s.png Minimum FDR: 0.941.

H

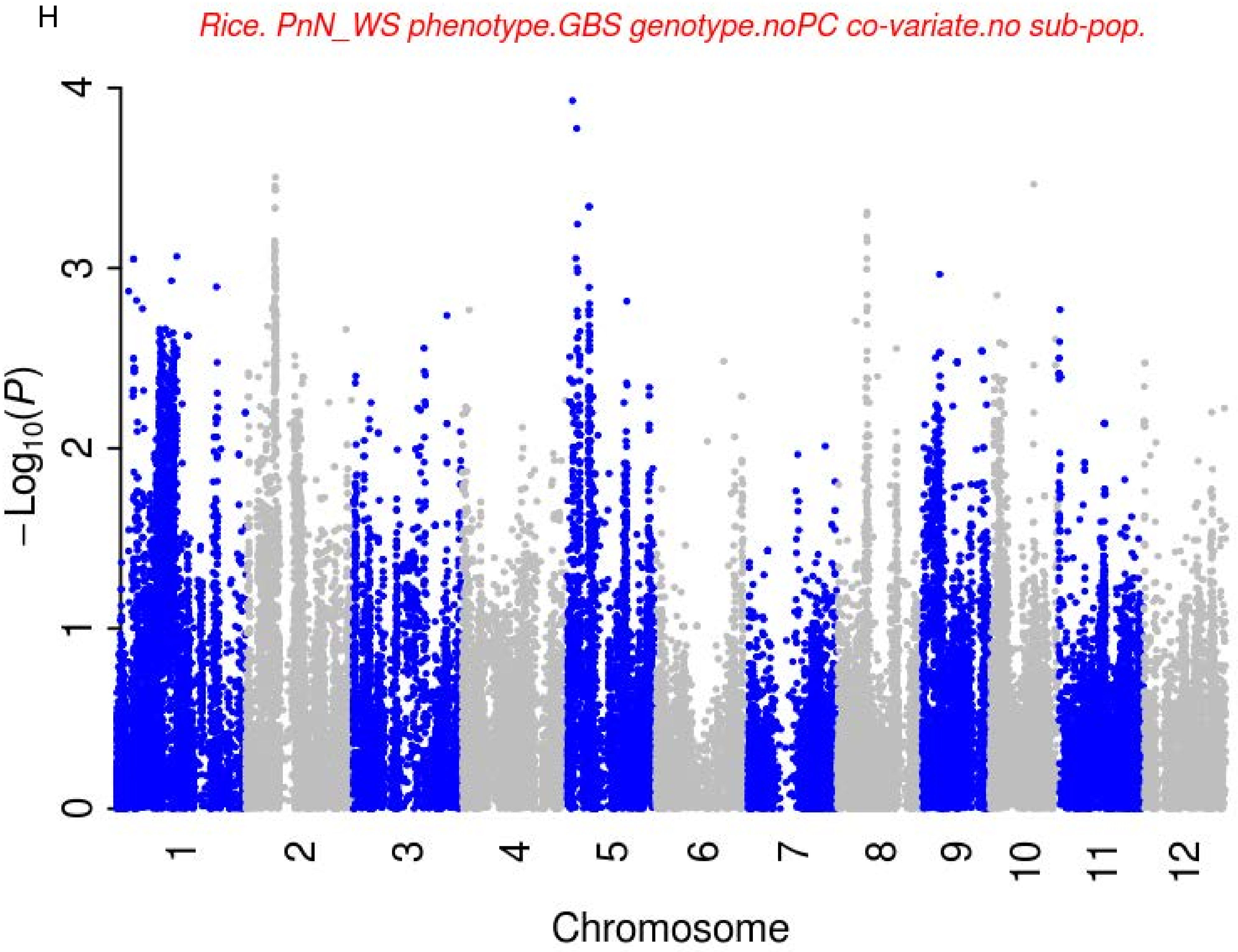

2014-05-20 15:14 MP_PnN.GBS_JS_WS.s.png Minimum FDR: 0.651.

Rice. PnN_DS phenotype.GBS genotype.noPC co-variate.no sub-pop.

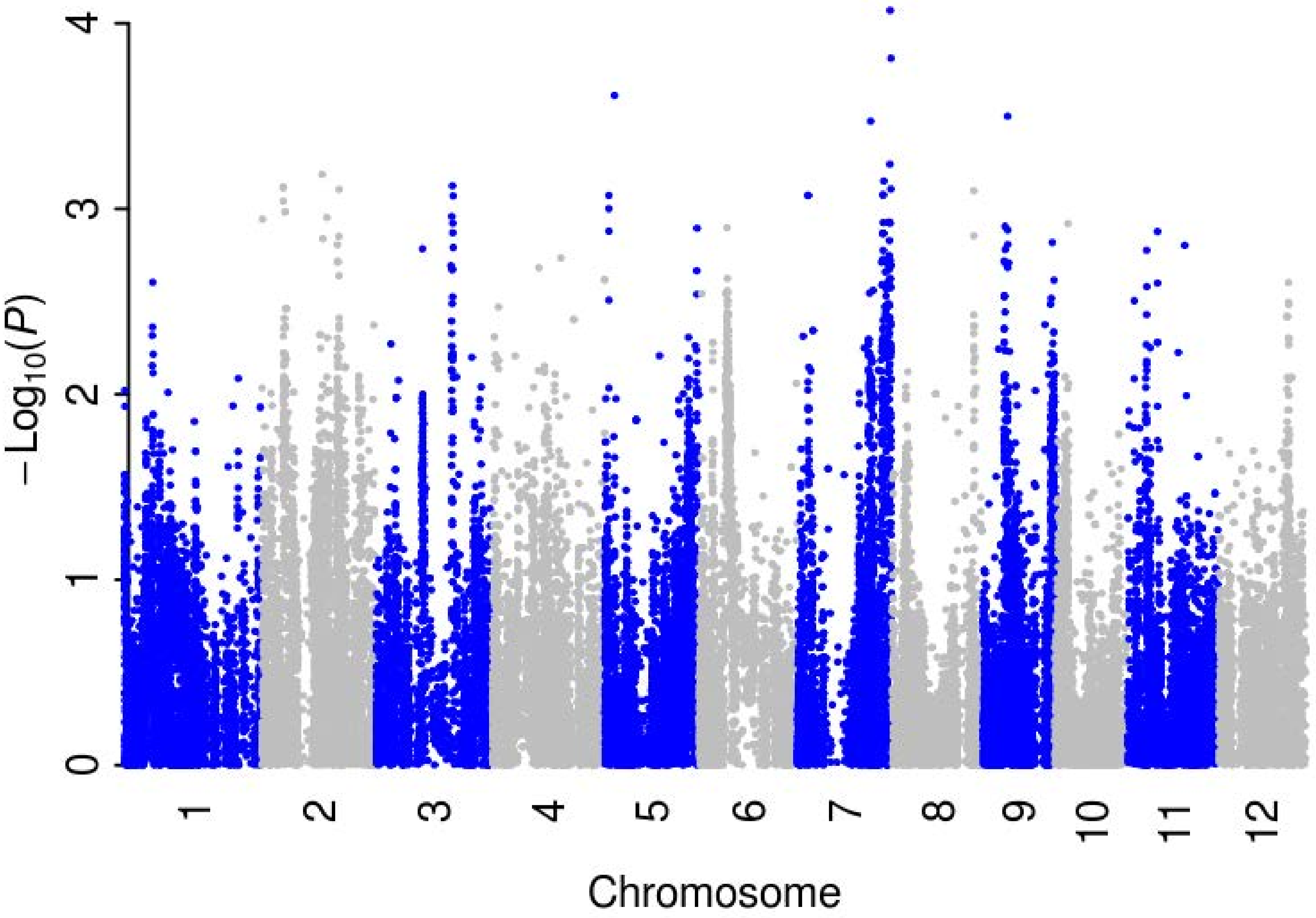

2014-05-20 15:12 MP_PnN.GBS_JS_DS.s.png Minimum FDR: 0.832.

J

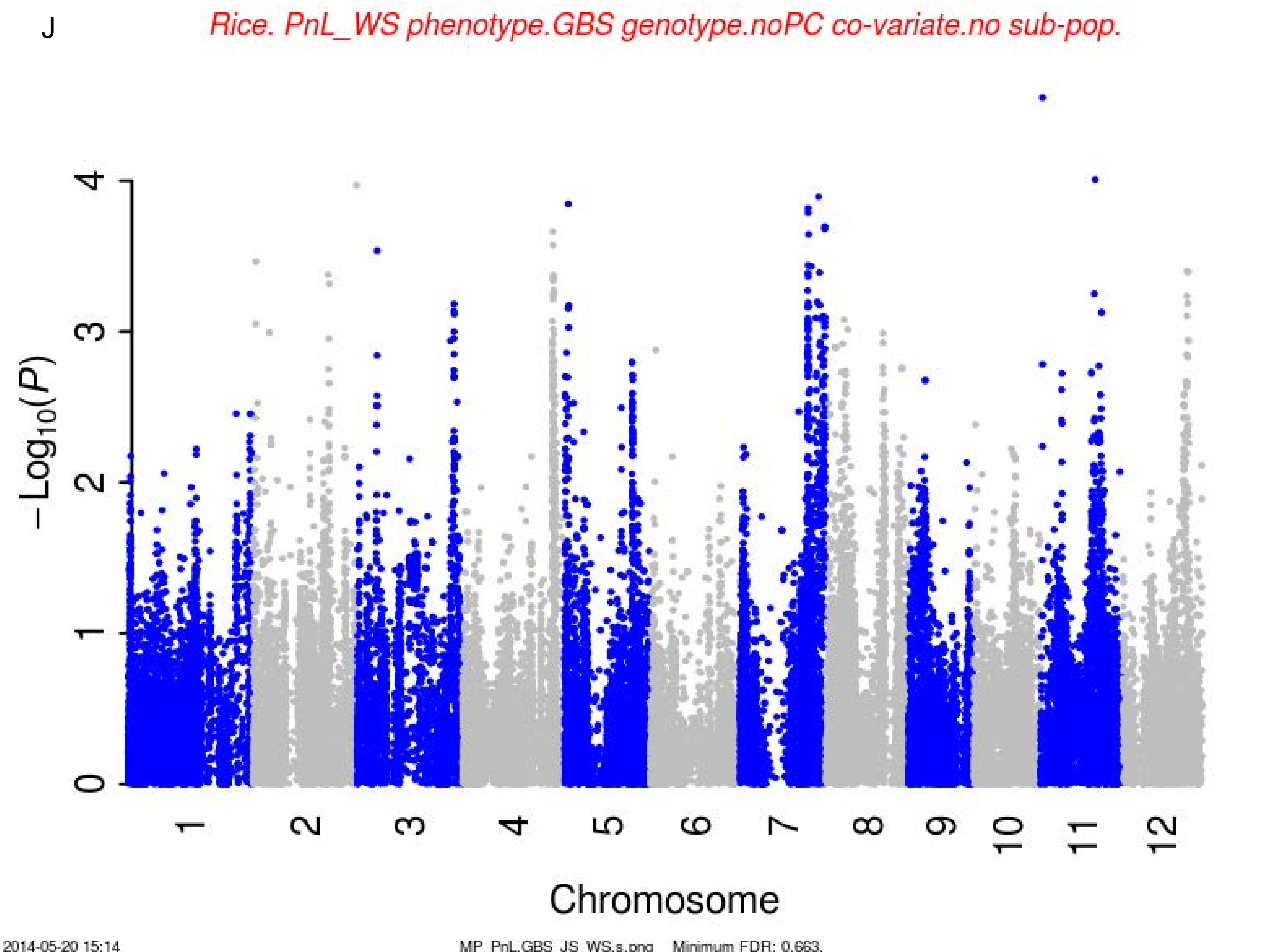
Rice. PnL_WS phenotype.GBS genotype.noPC co-variate.no sub-pop.
$-\mathrm{Log}_{10}(P)$
0
1
2
3
4
1
2
3
4
5
6
7
8
9
10
11
12
Chromosome
2014-05-20 15:14
MP_PnL.GBS_JS_WS.s.png Minimum FDR: 0.663.

K

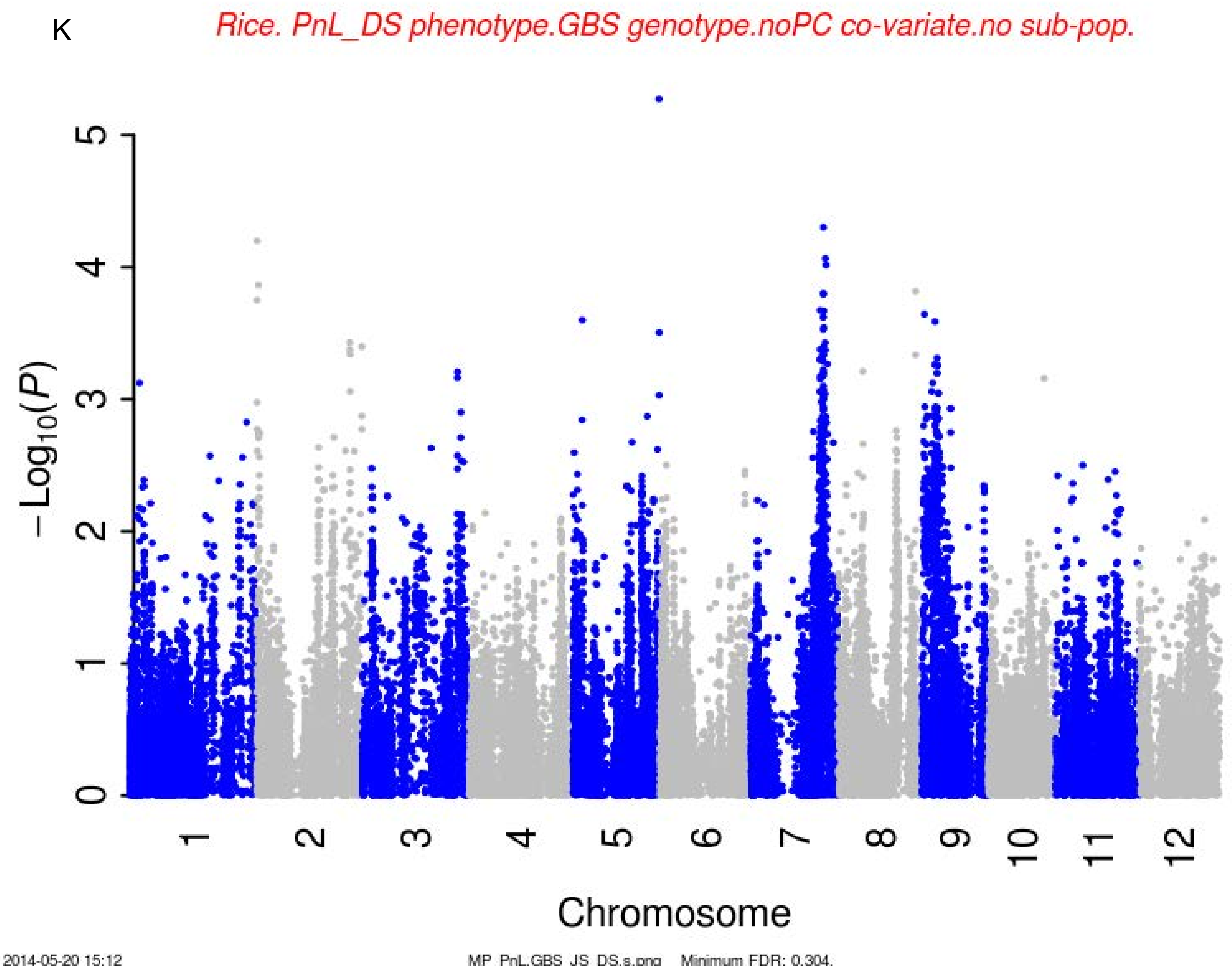

Rice. PnL_DS phenotype.GBS genotype.noPC co-variate.no sub-pop.
$-\mathrm{Log}_{10}(P)$
0
1
2
3
4
5
1
2
3
4
5
6
7
8
9
10
11
12
Chromosome
2014-05-20 15:12
MP_PnL.GBS_JS_DS.s.png
Minimum FDR: 0.304.

L

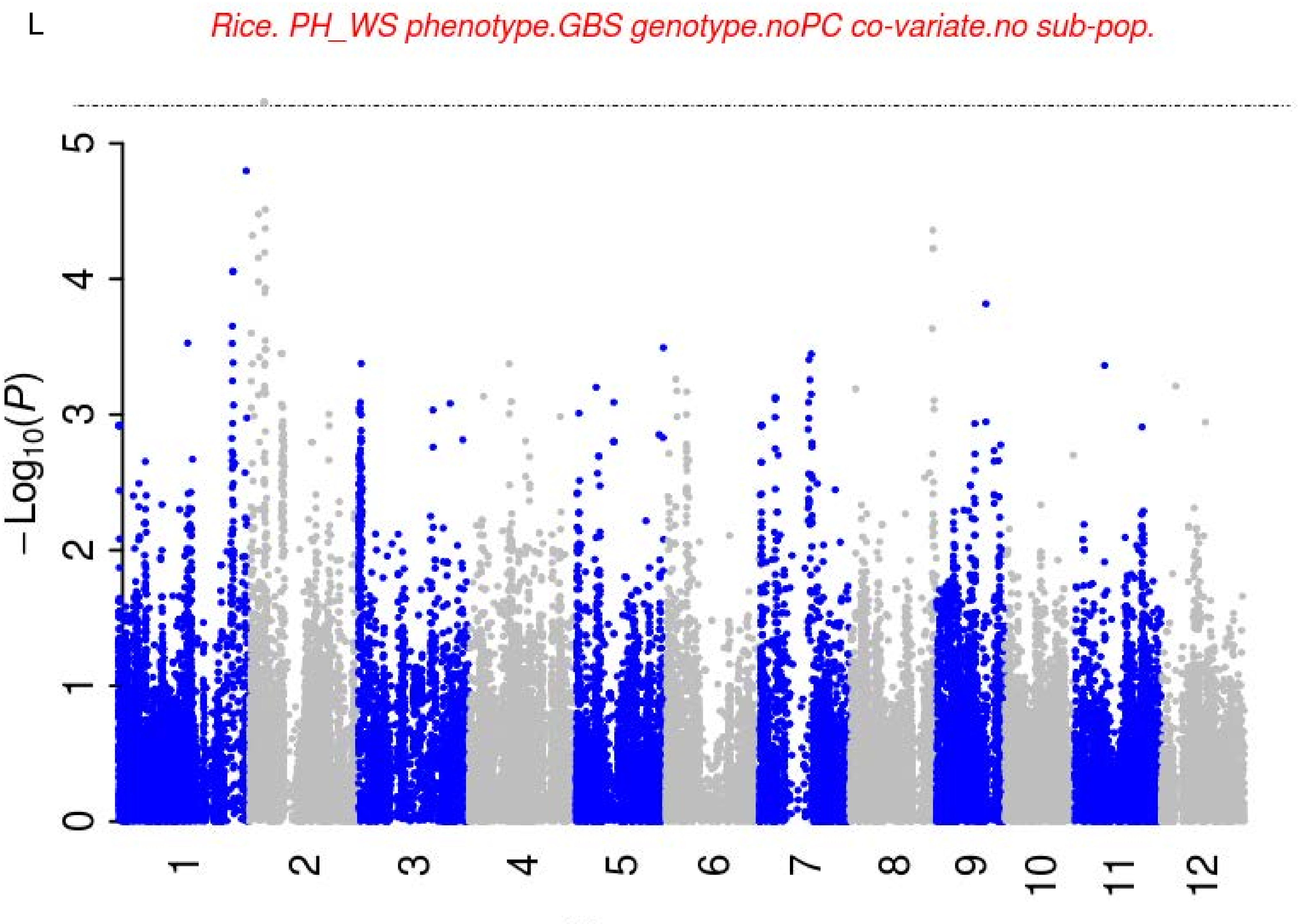

2014-05-20 15:13 MP_PH.GBS_JS_WS.s.png Minimum FDR: 0.096.

M

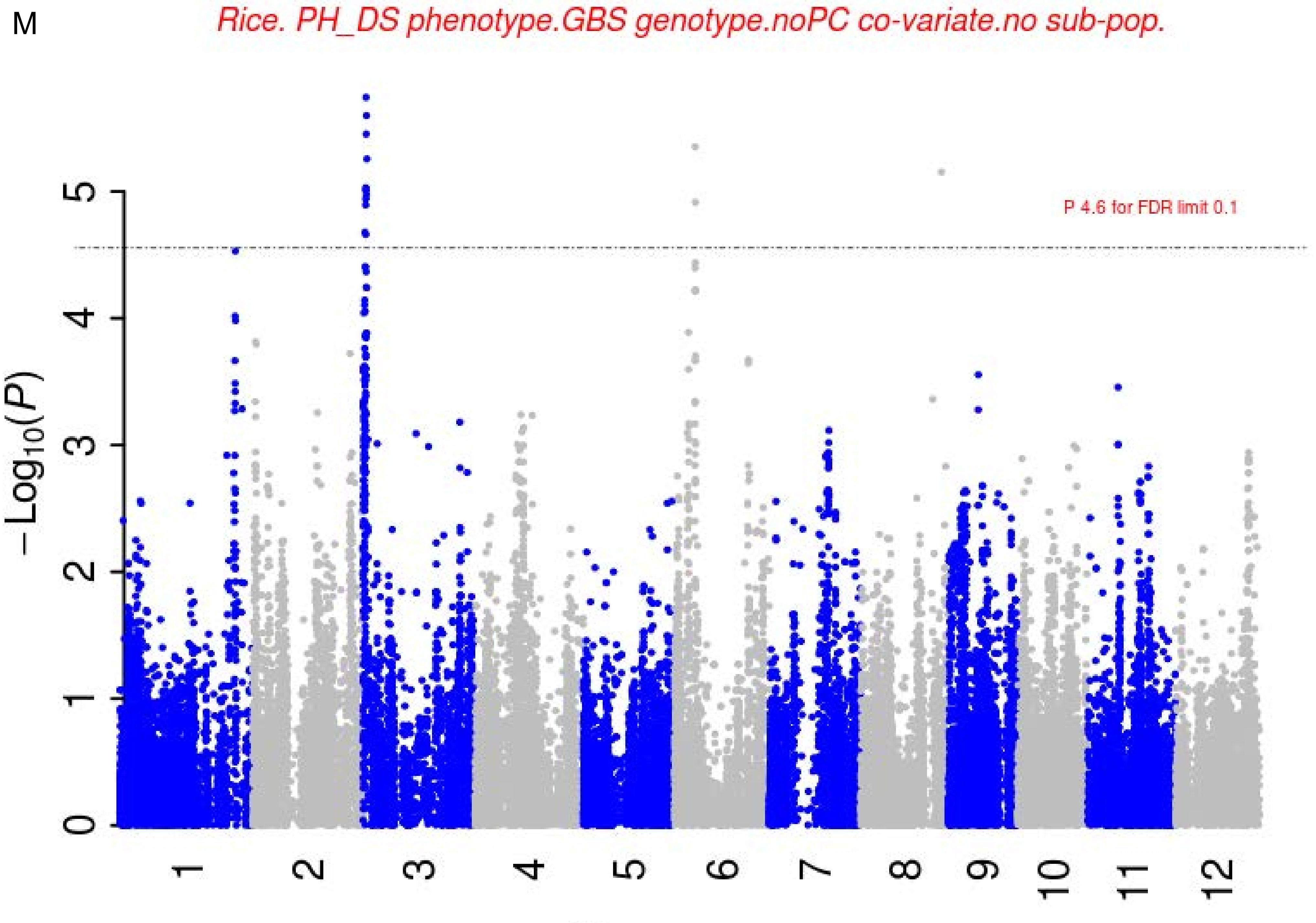

2014-05-20 15:11 MP_PH.GBS_JS_DS.s.png Minimum FDR: 0.056.

N

Rice. PedL_WS phenotype.GBS genotype.noPC co-variate.no sub-pop.

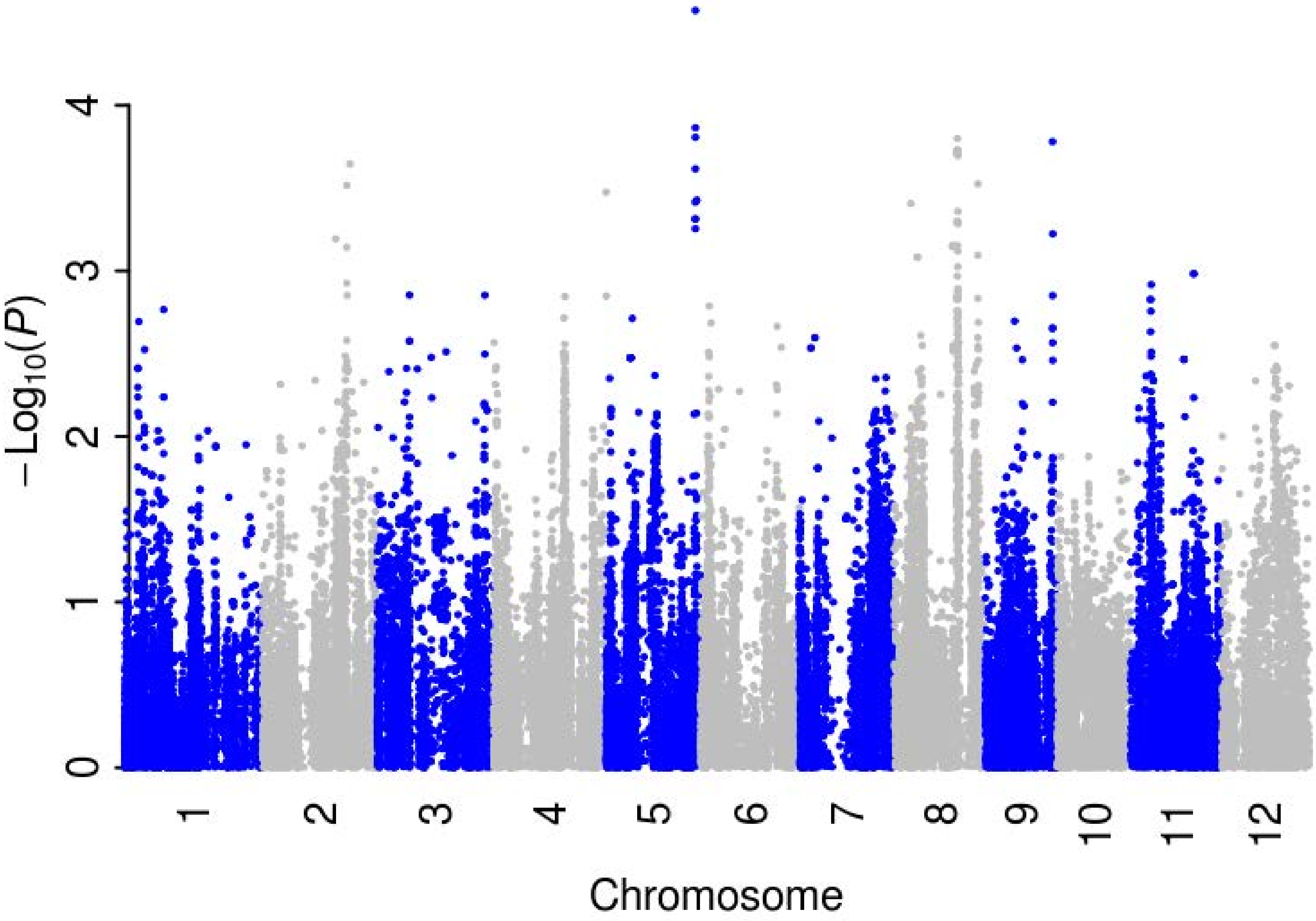

2014-05-20 15:14 MP_PedL.GBS_JS_WS.s.png Minimum FDR: 0.832.

O

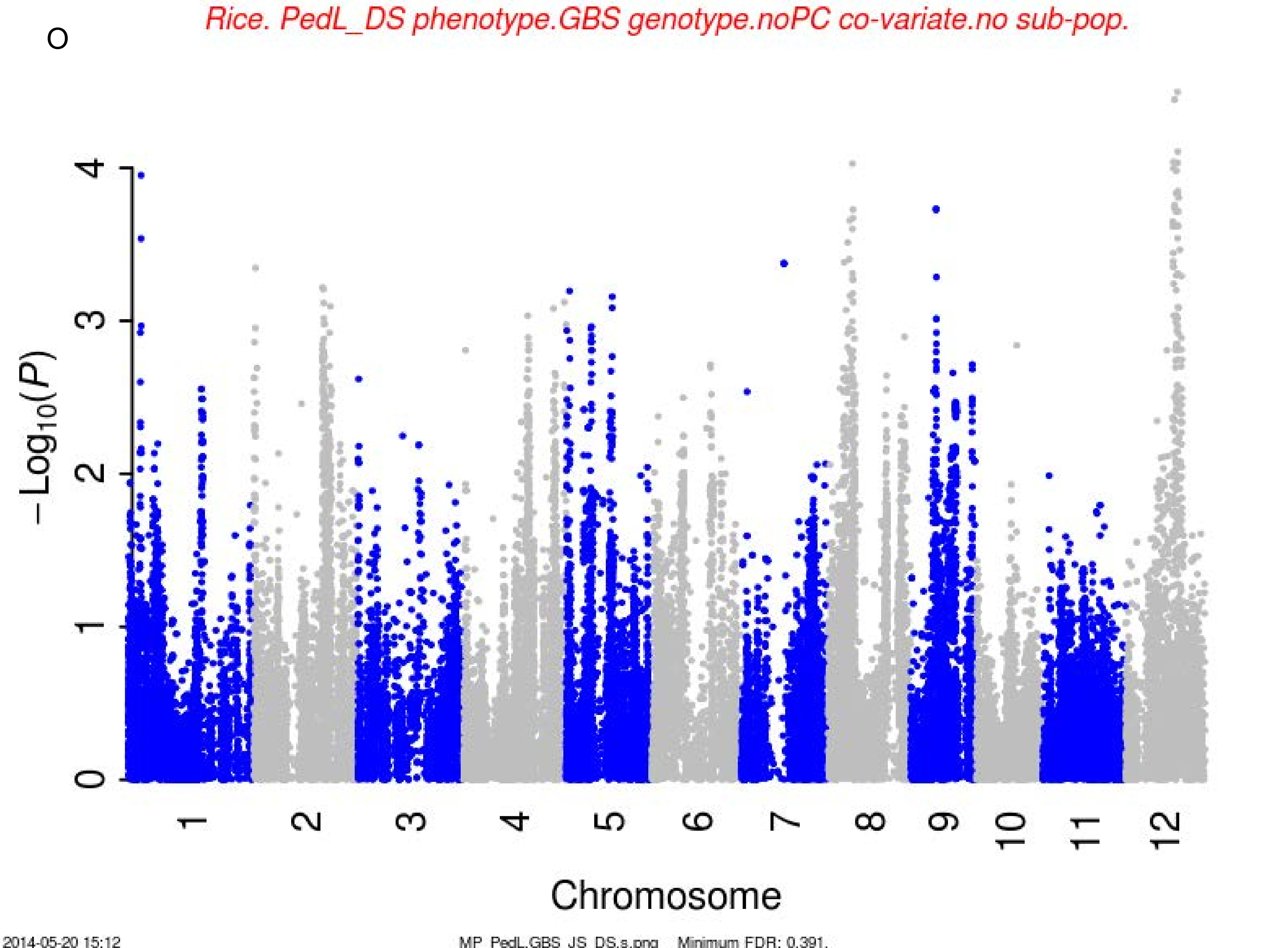
Rice. PedL_DS phenotype.GBS genotype.noPC co-variate.no sub-pop.
−Log10(P)
0
1
2
3
4
1
2
3
4
5
6
7
8
9
10
11
12
Chromosome
2014-05-20 15:12
MP_PedL.GBS_JS_DS.s.png Minimum FDR: 0.391.

P

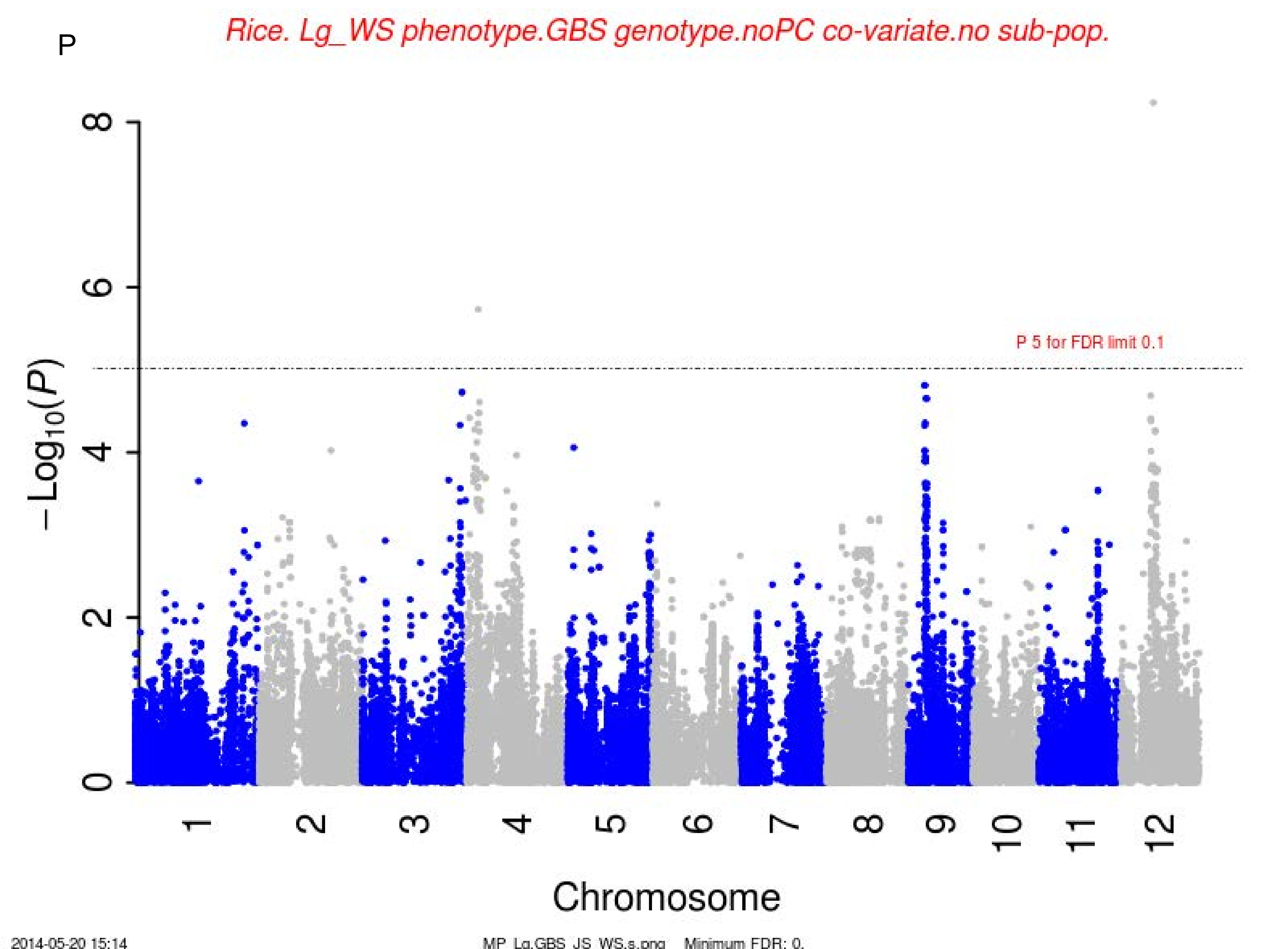
Rice. Lg_WS phenotype.GBS genotype.noPC co-variate.no sub-pop.
P 5 for FDR limit 0.1
−Log10(P)
0
2
4
6
8
1
2
3
4
5
6
7
8
9
10
11
12
Chromosome
2014-05-20 15:14
MP_Lg.GBS_JS_WS.s.png
Minimum FDR: 0.

Q

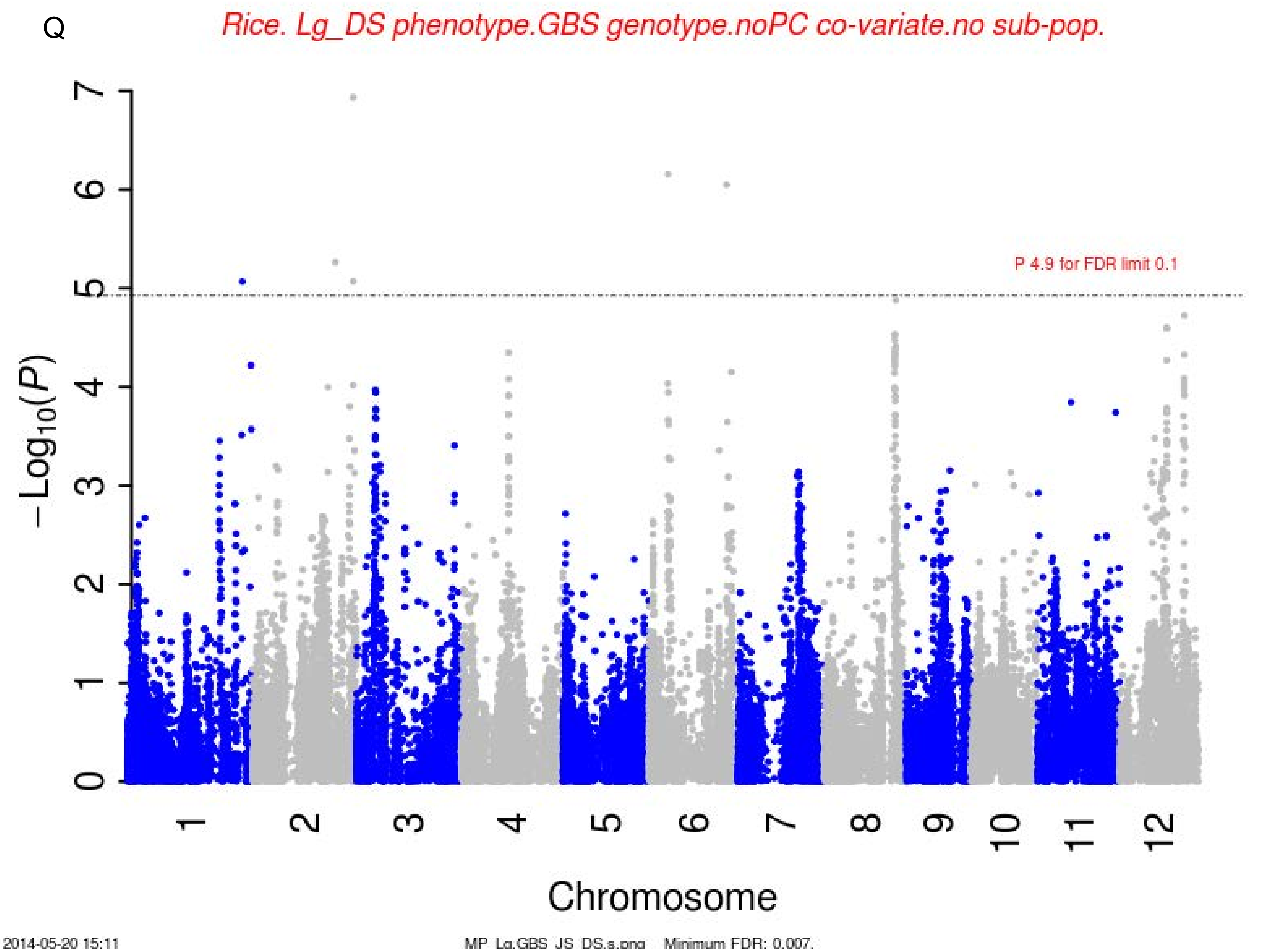

Rice. Lg_DS phenotype.GBS genotype.noPC co-variate.no sub-pop.
P 4.9 for FDR limit 0.1
$-\mathrm{Log}_{10}(P)$
0
1
2
3
4
5
6
7
1
2
3
4
5
6
7
8
9
10
11
12
Chromosome
2014-05-20 15:11
MP_Lg.GBS_JS_DS.s.png Minimum FDR: 0.007.

R

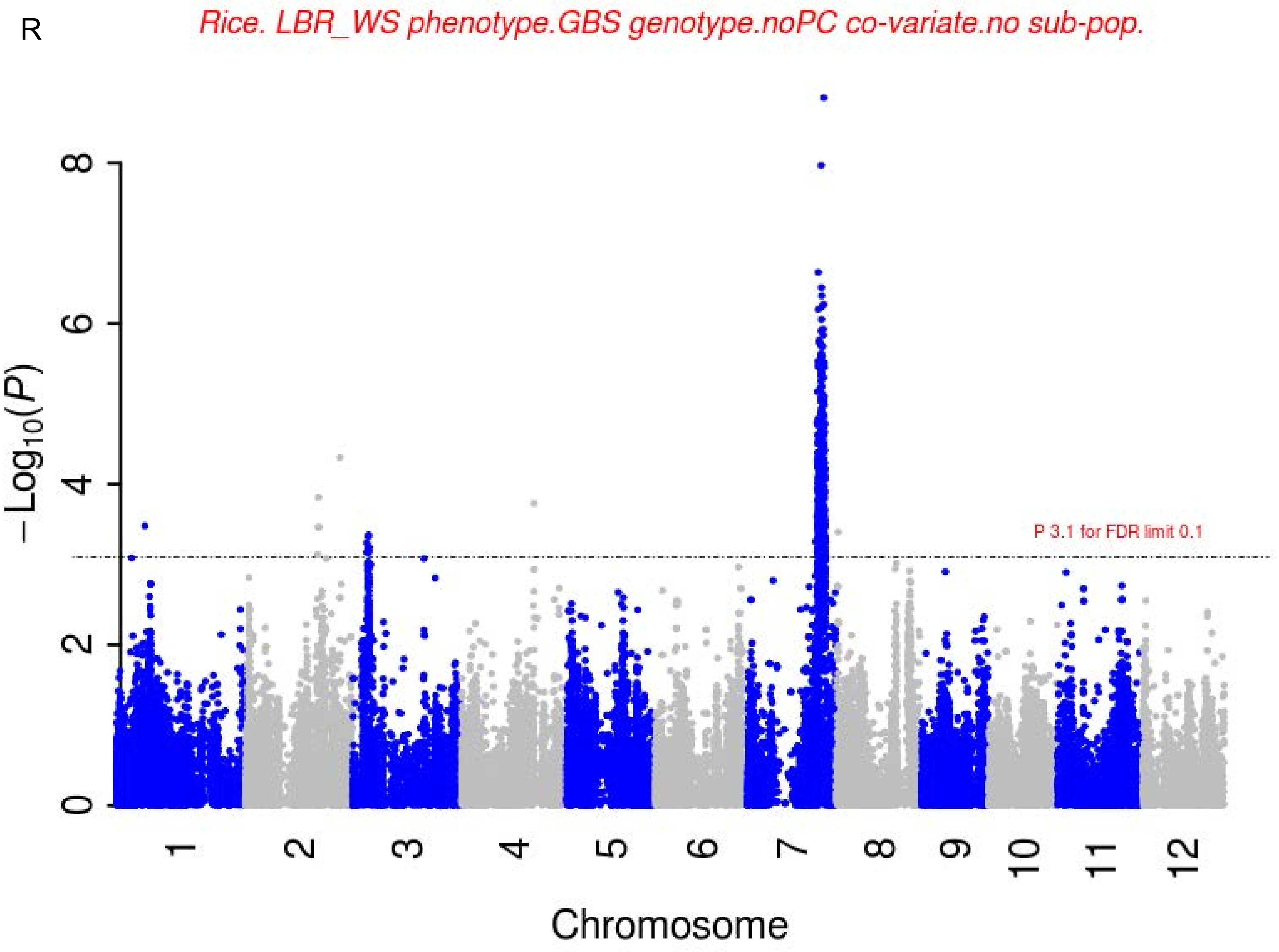

2014-05-20 15:15 MP_LBR.GBS_JS_WS.s.png Minimum FDR: 0.

S

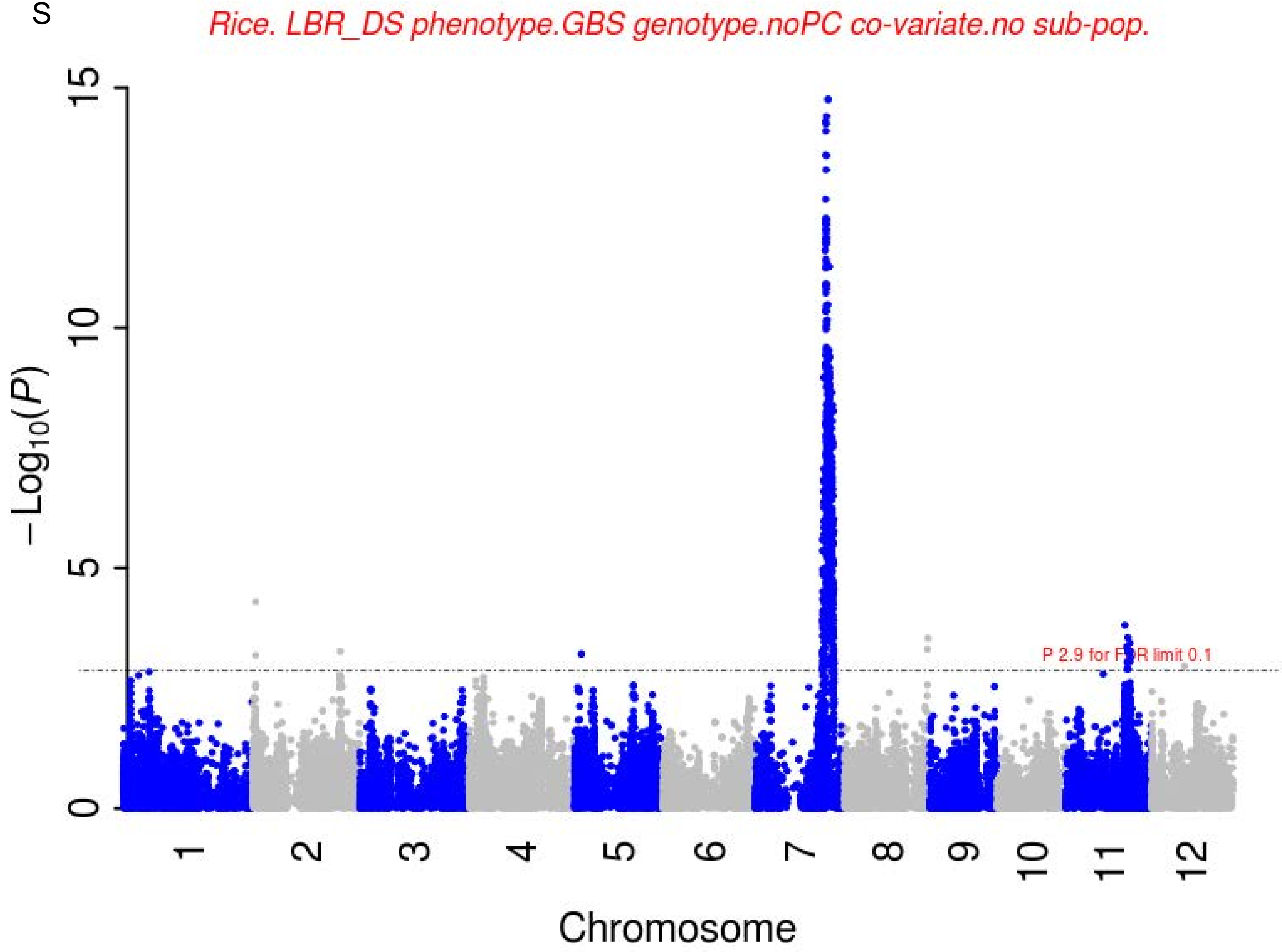

2014-05-20 15:13 MP_LBR.GBS_JS_DS.s.png Minimum FDR: 0.

T

Rice. GrW_WS phenotype.GBS genotype.noPC co-variate.no sub-pop.

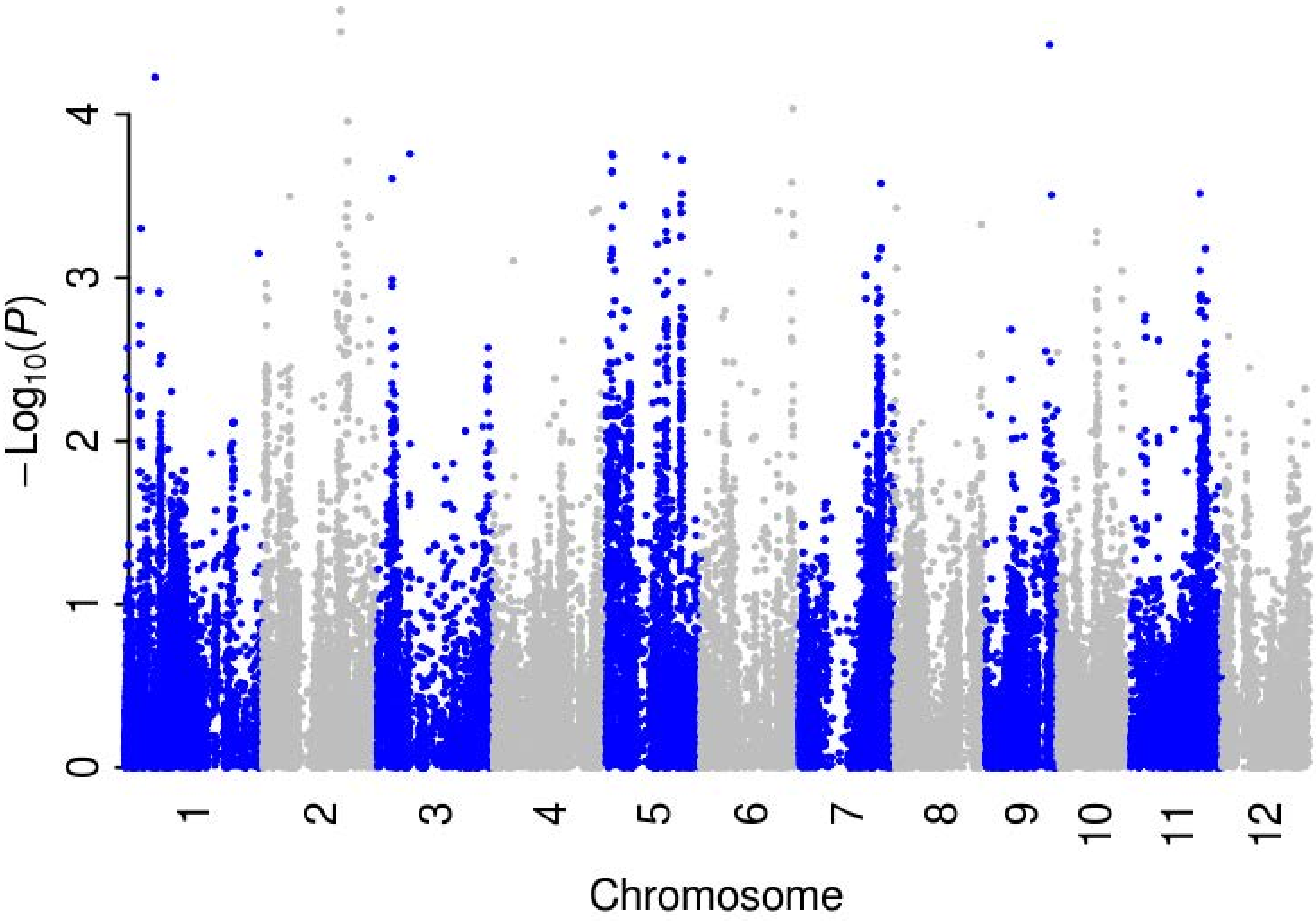

2014-05-20 15:15 MP_GrW.GBS_JS_WS.s.png Minimum FDR: 0.432.

U

Rice. GrW_DS phenotype.GBS genotype.noPC co-variate.no sub-pop.

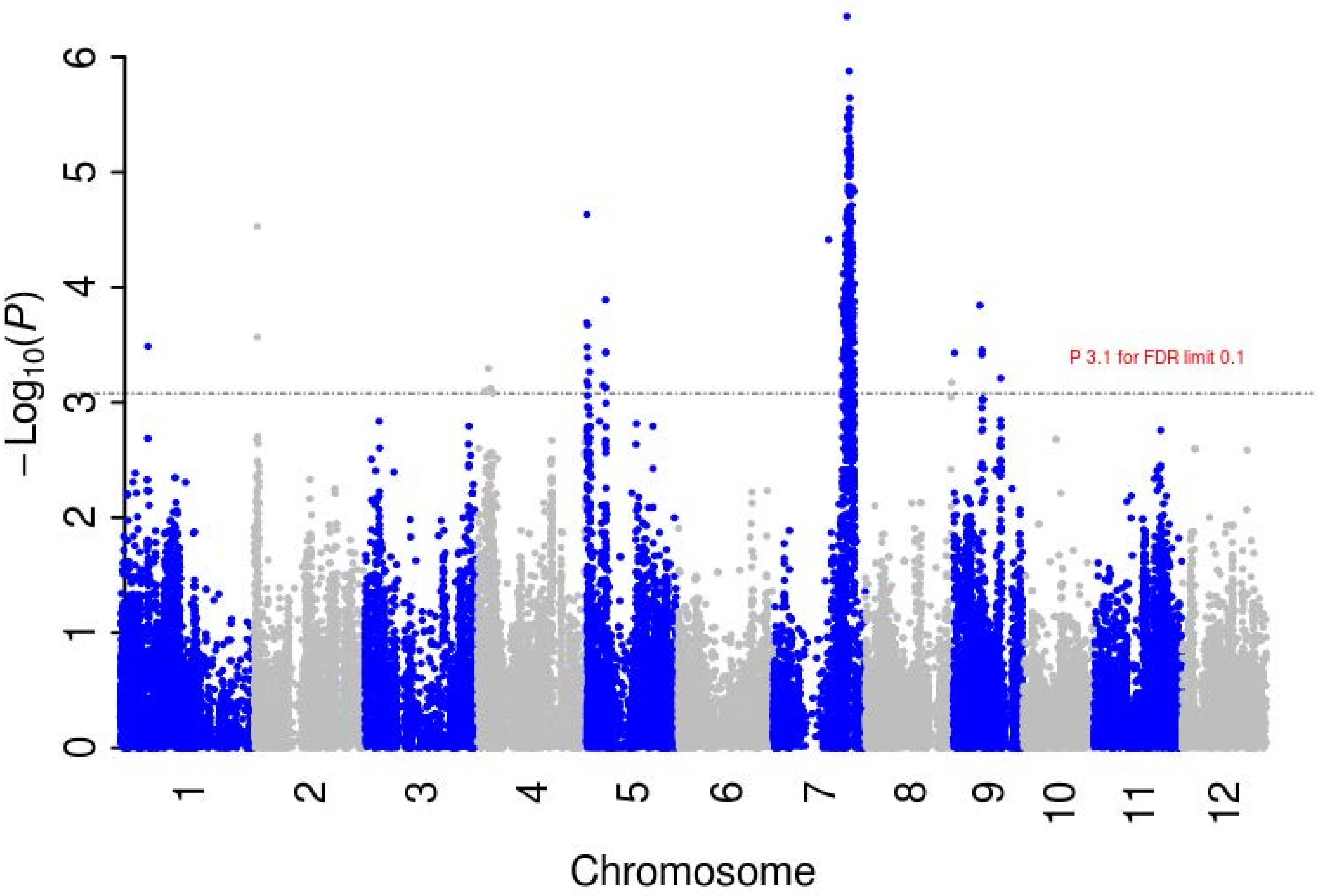

2014-05-20 15:13 MP_GrW.GBS_JS_DS.s.png Minimum FDR: 0.012.

V

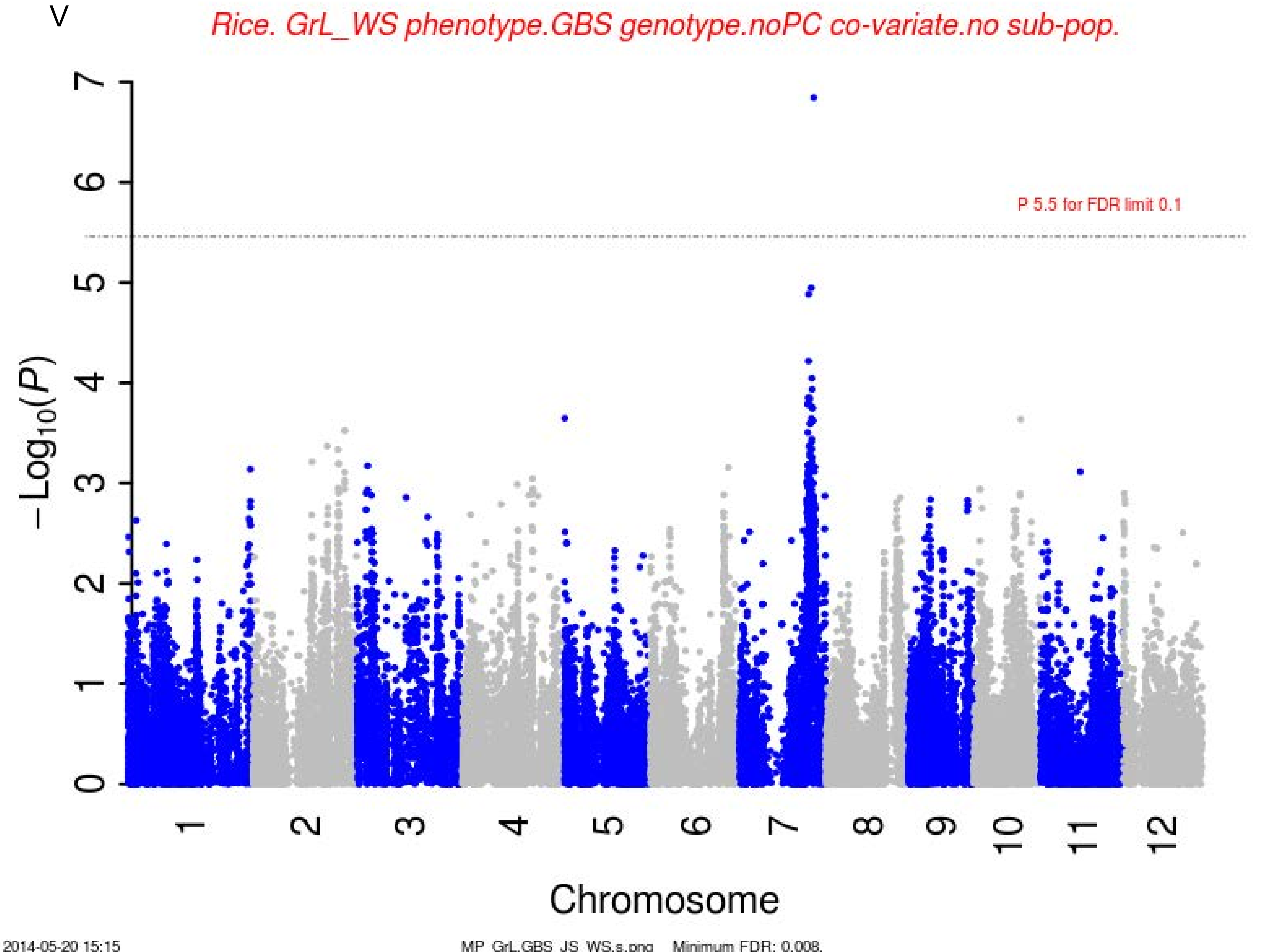
Rice. GrL_WS phenotype.GBS genotype.noPC co-variate.no sub-pop.
P 5.5 for FDR limit 0.1
$-Log_{10}(P)$
0
1
2
3
4
5
6
7
1
2
3
4
5
6
7
8
9
10
11
12
Chromosome
2014-05-20 15:15
MP_GrL.GBS_JS_WS.s.png
Minimum FDR: 0.008.

W

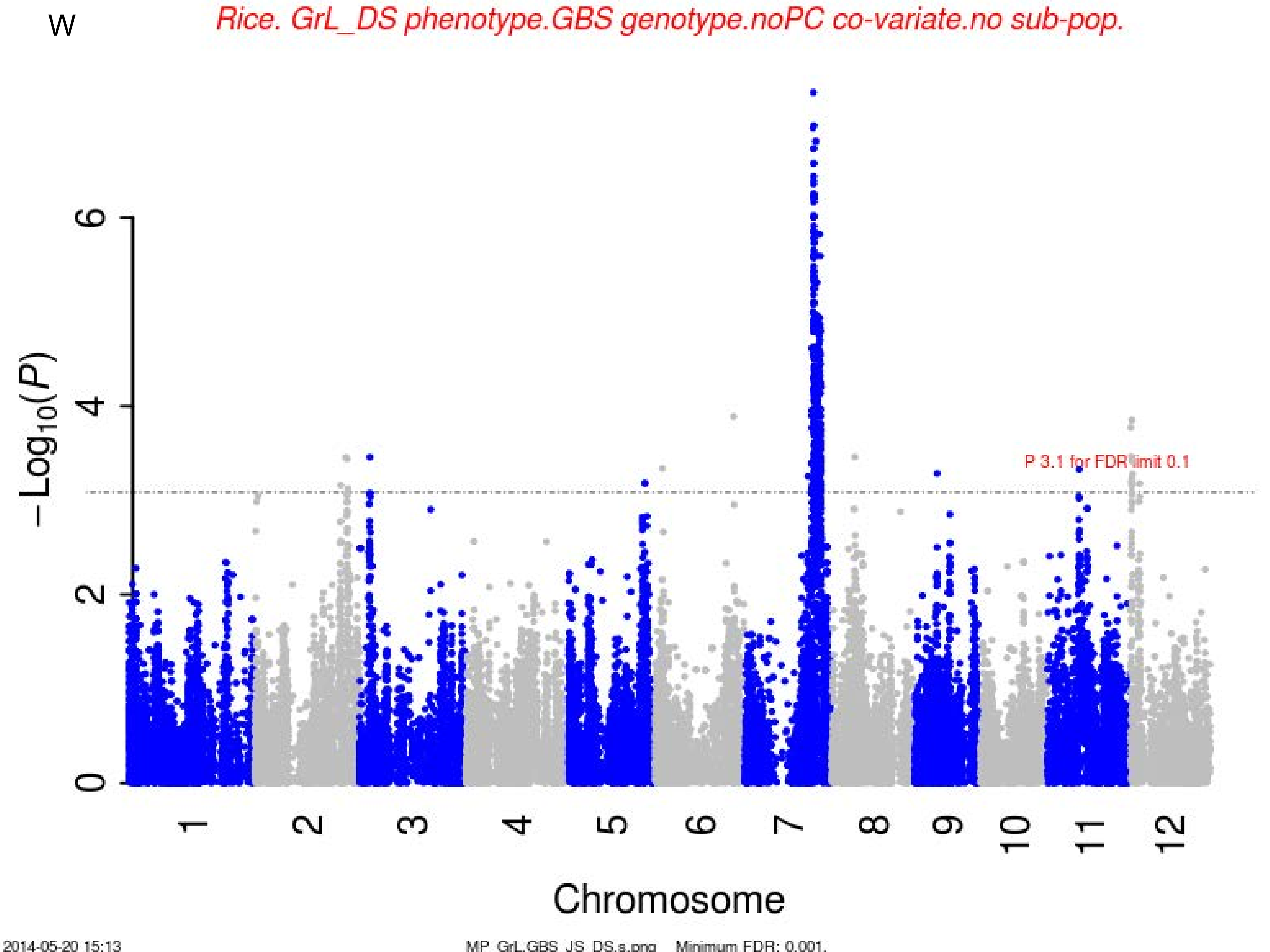

Rice. GrL_DS phenotype.GBS genotype.noPC co-variate.no sub-pop.
P 3.1 for FDR limit 0.1
−Log10(P)
0
2
4
6
1
2
3
4
5
6
7
8
9
10
11
12
Chromosome
2014-05-20 15:13
MP_GrL.GBS_JS_DS.s.png Minimum FDR: 0.001.

X

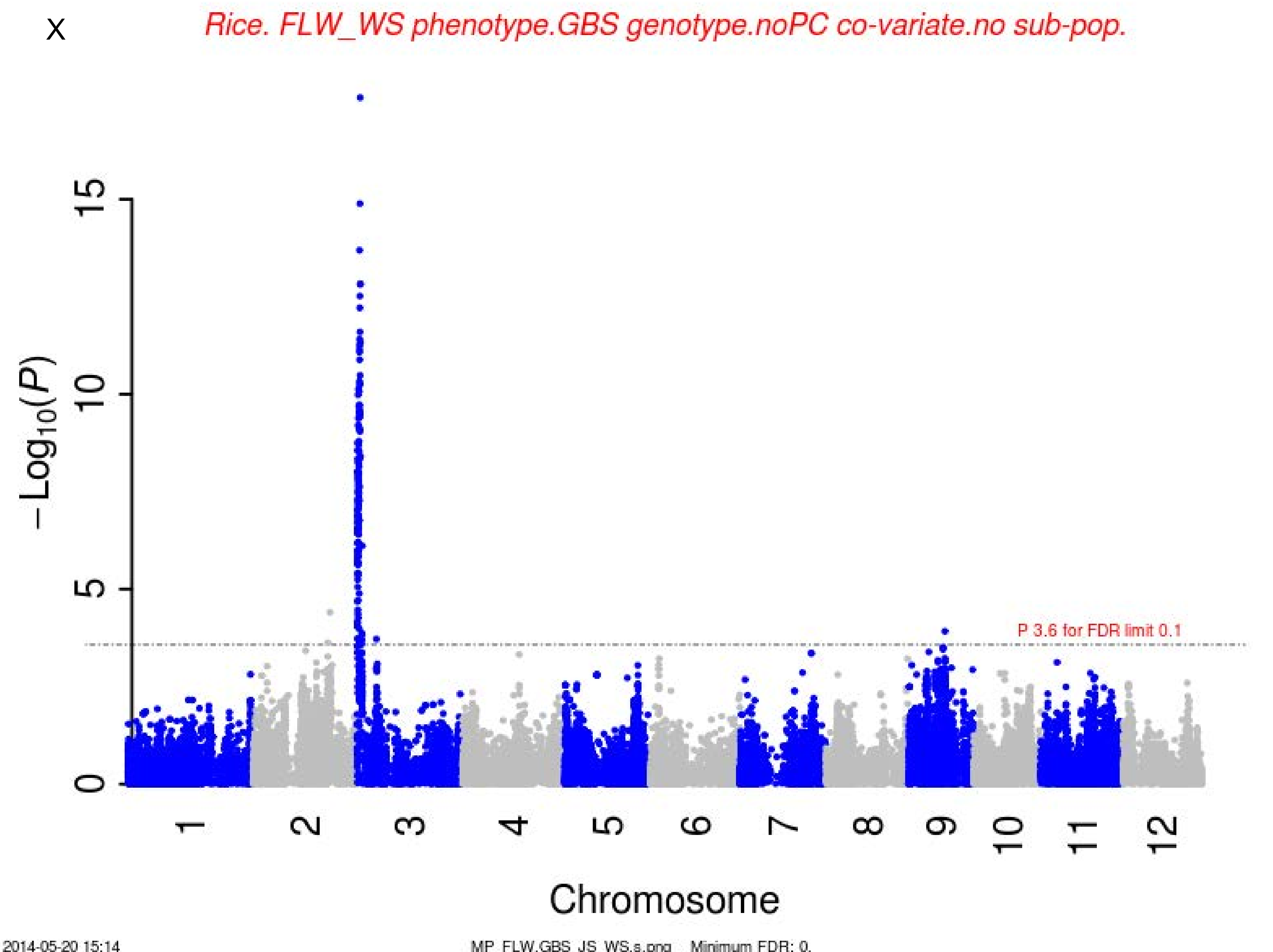
Rice. FLW_WS phenotype.GBS genotype.noPC co-variate.no sub-pop.
$-\mathrm{Log}_{10}(P)$
0
5
10
15
P 3.6 for FDR limit 0.1
1
2
3
4
5
6
7
8
9
10
11
12
Chromosome
2014-05-20 15:14
MP_FLW.GBS_JS_WS.s.png
Minimum FDR: 0.

Y

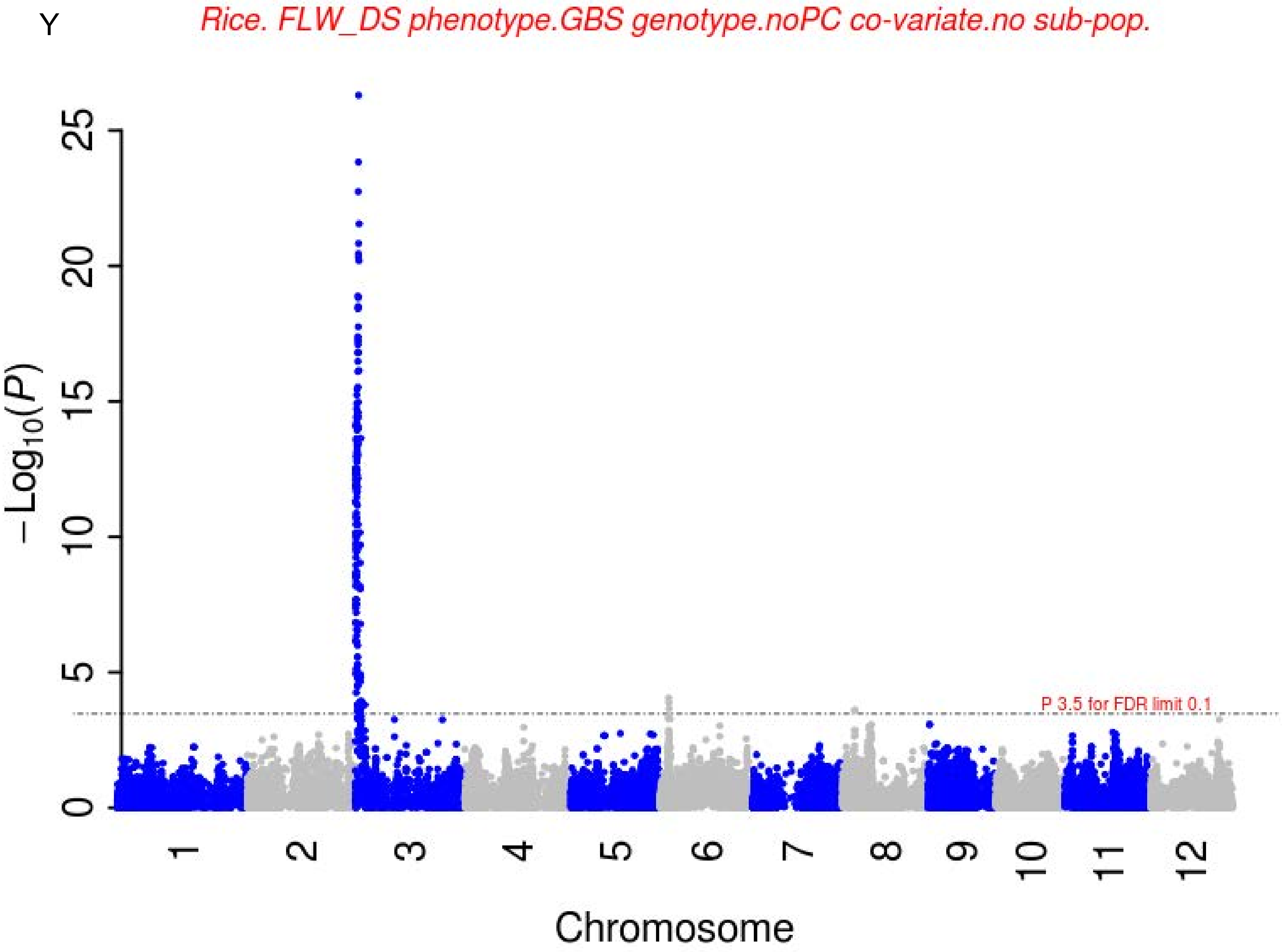

2014-05-20 15:11 MP_FLW.GBS_JS_DS.s.png Minimum FDR: 0.

Z

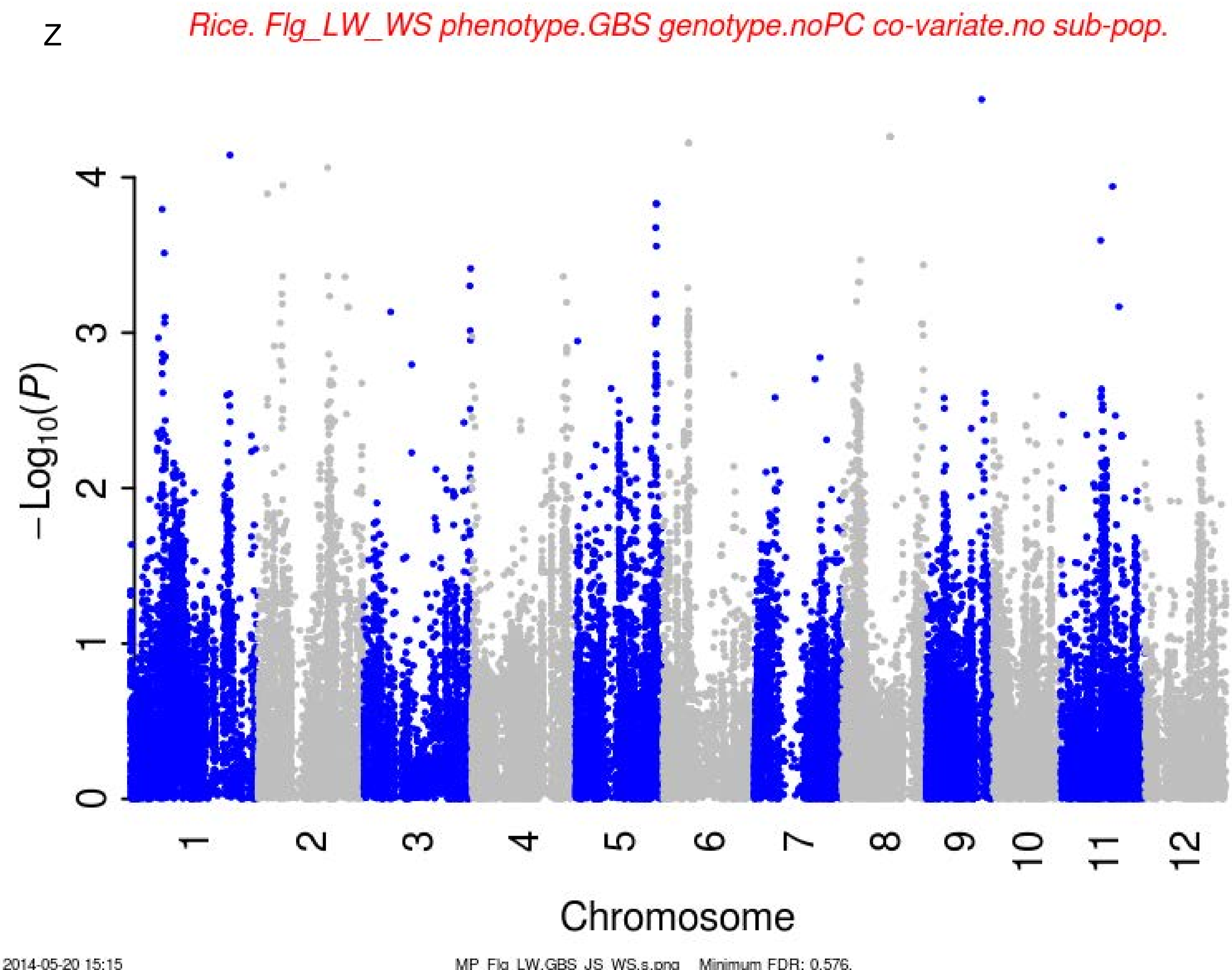
Rice. Flg_LW_WS phenotype.GBS genotype.noPC co-variate.no sub-pop.
−Log10(P)
0
1
2
3
4
1
2
3
4
5
6
7
8
9
10
11
12
Chromosome
2014-05-20 15:15
MP_Flg_LW.GBS_JS_WS.s.png Minimum FDR: 0.576.

AA

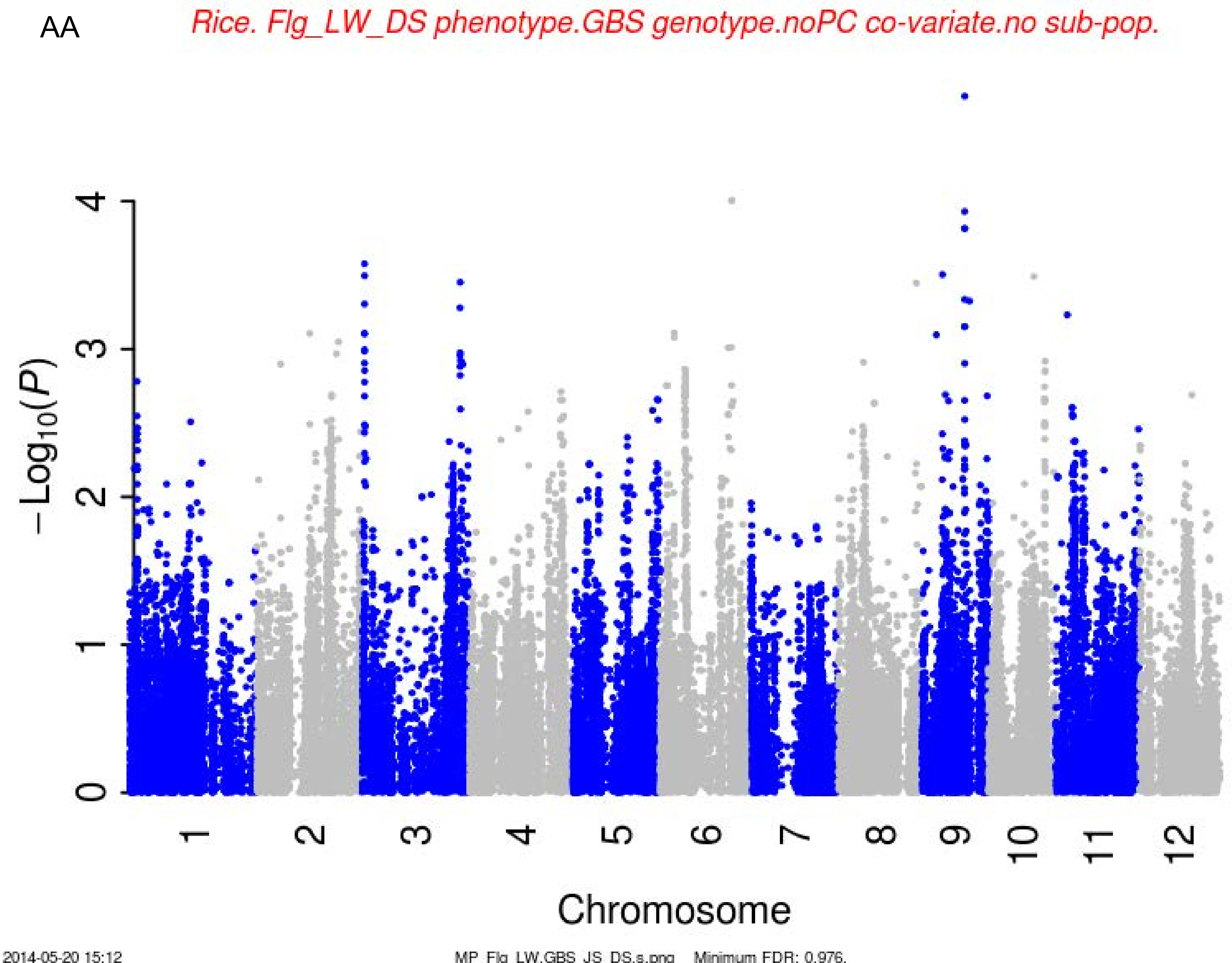
Rice. Flg_LW_DS phenotype.GBS genotype.noPC co-variate.no sub-pop.
$-\mathrm{Log}_{10}(P)$
0
1
2
3
4
1
2
3
4
5
6
7
8
9
10
11
12
Chromosome
2014-05-20 15:12
MP_Flg_LW.GBS_JS_DS.s.png Minimum FDR: 0.976.

AB

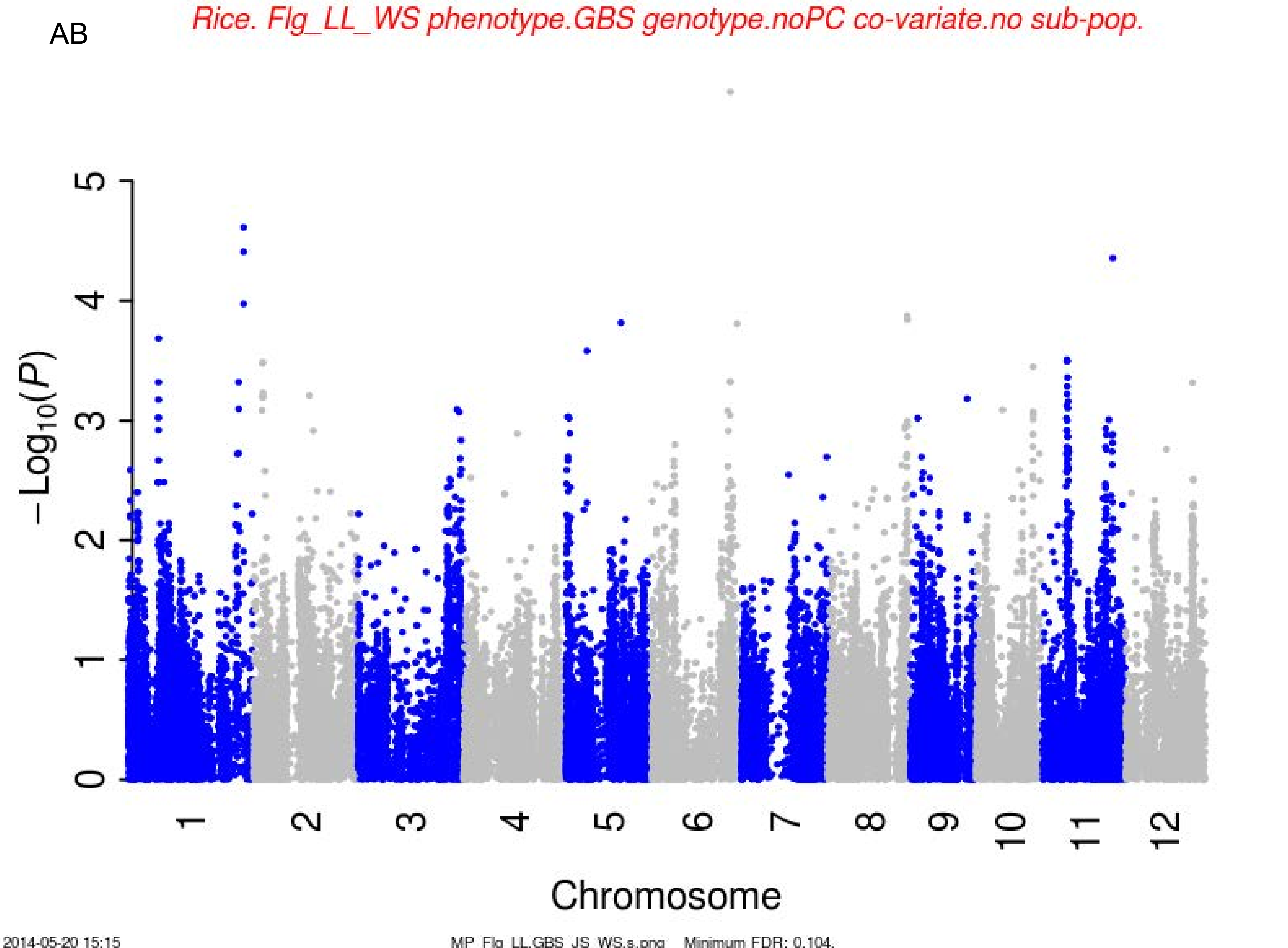

Rice. Flg_LL_WS phenotype.GBS genotype.noPC co-variate.no sub-pop.
−Log10(P)
0
1
2
3
4
5
1
2
3
4
5
6
7
8
9
10
11
12
Chromosome
2014-05-20 15:15
MP Flg LL.GBS JS WS.s.png Minimum FDR: 0.104.

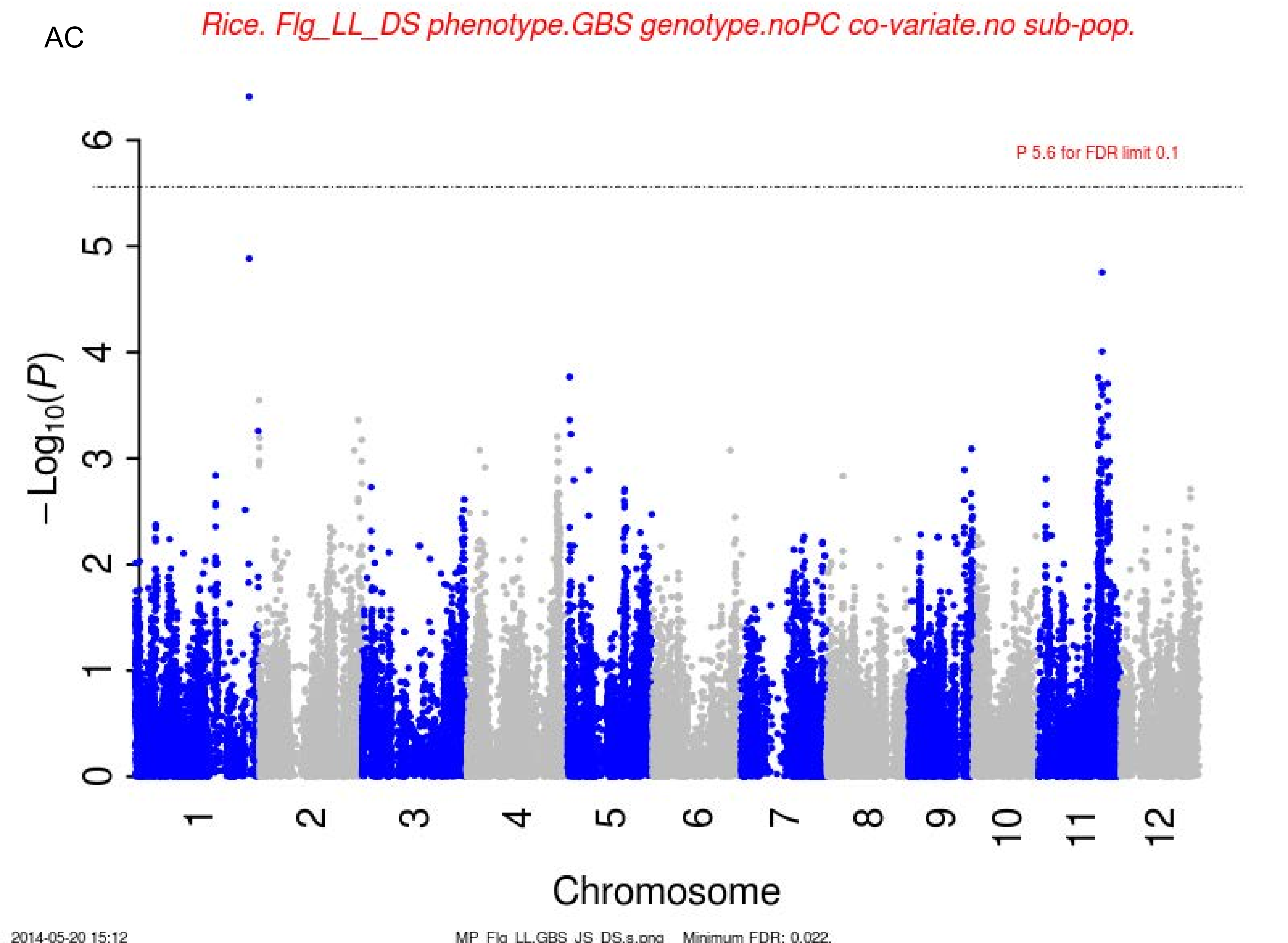
AC
Rice. Flg_LL_DS phenotype.GBS genotype.noPC co-variate.no sub-pop.
P 5.6 for FDR limit 0.1
$-\mathrm{Log}_{10}(P)$
0
1
2
3
4
5
6
1
2
3
4
5
6
7
8
9
10
11
12
Chromosome
2014-05-20 15:12
MP_Flg_LL.GBS_JS_DS.s.png
Minimum FDR: 0.022.

AD

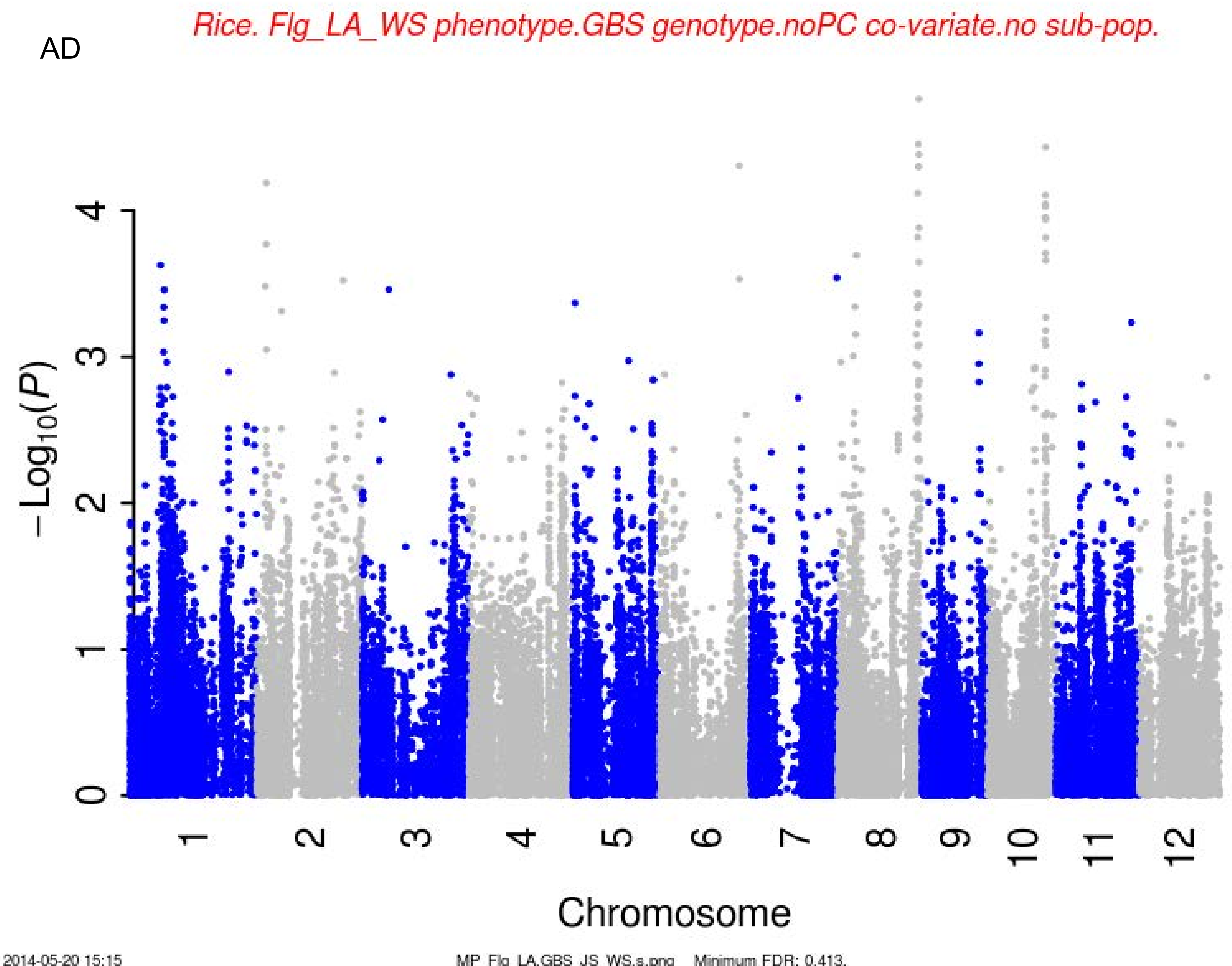
Rice. Flg_LA_WS phenotype.GBS genotype.noPC co-variate.no sub-pop.
$-\mathrm{Log}_{10}(P)$
0
1
2
3
4
1
2
3
4
5
6
7
8
9
10
11
12
Chromosome
2014-05-20 15:15
MP_Flg_LA.GBS_JS_WS.s.png
Minimum FDR: 0.413.

AE

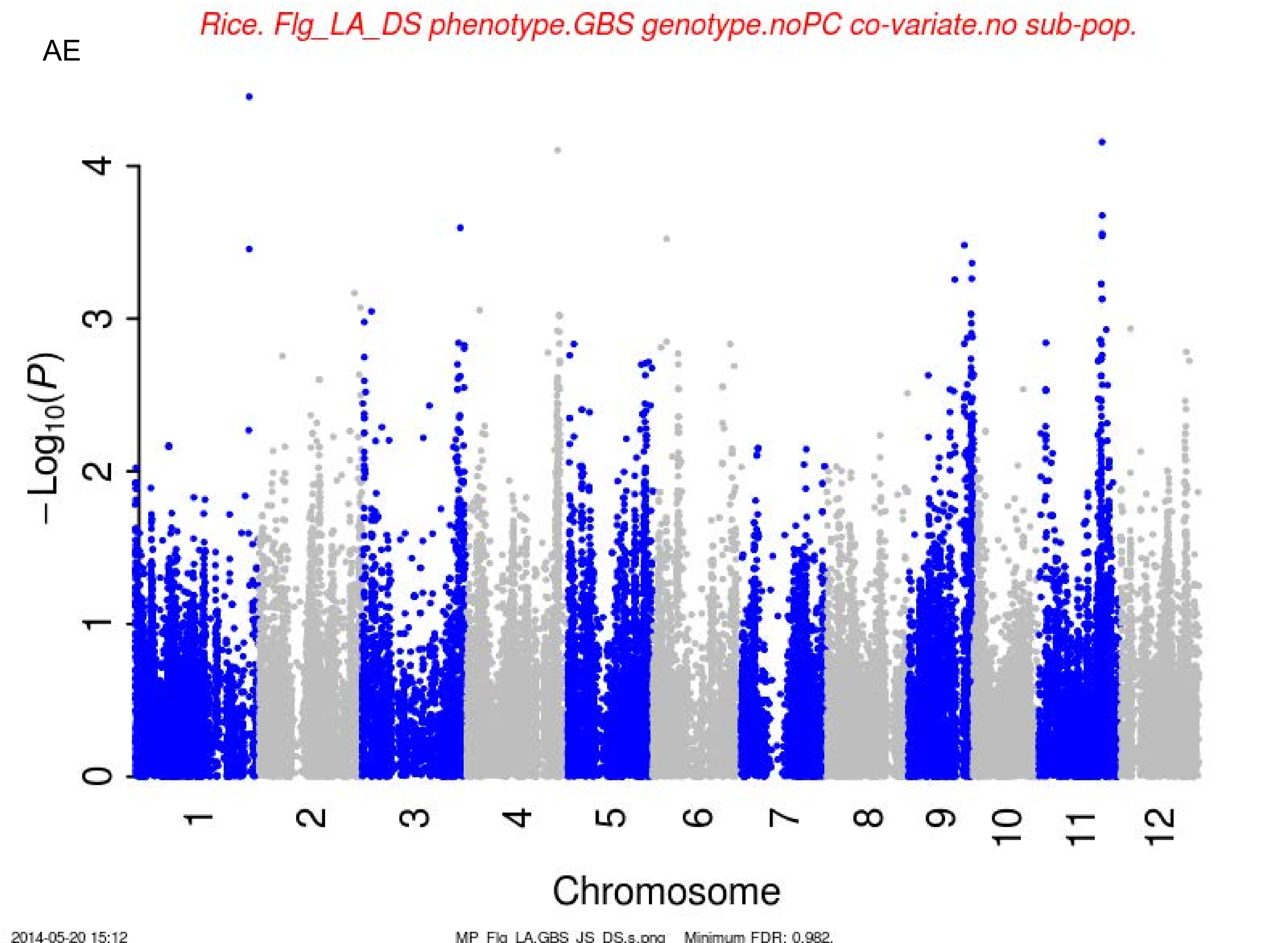
Rice. Flg_LA_DS phenotype.GBS genotype.noPC co-variate.no sub-pop.
−Log10(P)
0
1
2
3
4
1
2
3
4
5
6
7
8
9
10
11
12
Chromosome
2014-05-20 15:12
MP_Flg_LA.GBS_JS_DS.s.png   Minimum FDR: 0.982.

AF

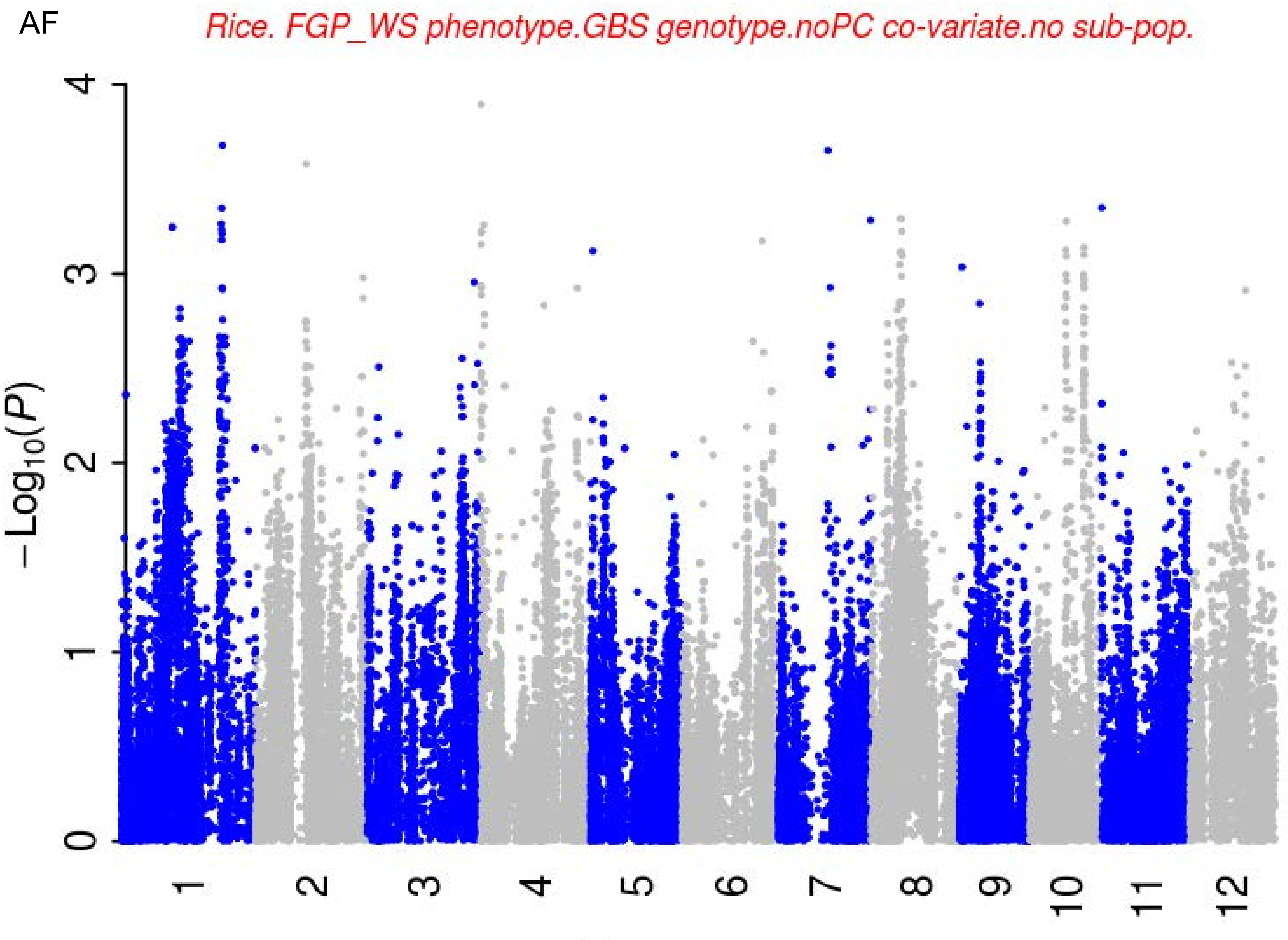

2014-05-20 15:15

MP_FGP.GBS_JS_WS.s.png Minimum FDR: 0.756.

AG

Rice. FGP_DS phenotype.GBS genotype.noPC co-variate.no sub-pop.

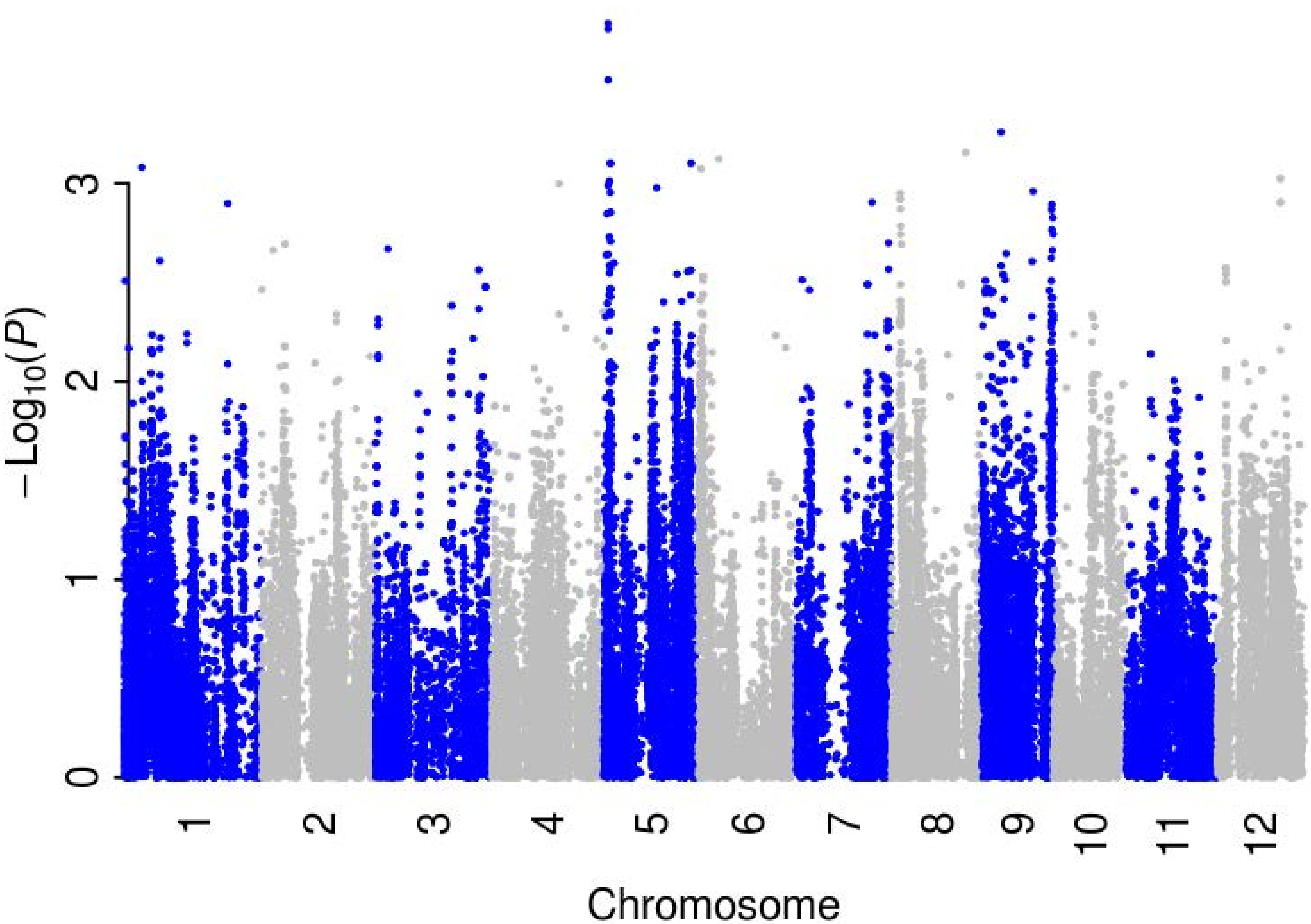

2014-05-20 15:13 MP_FGP.GBS_JS_DS.s.png Minimum FDR: 0.915.

AH

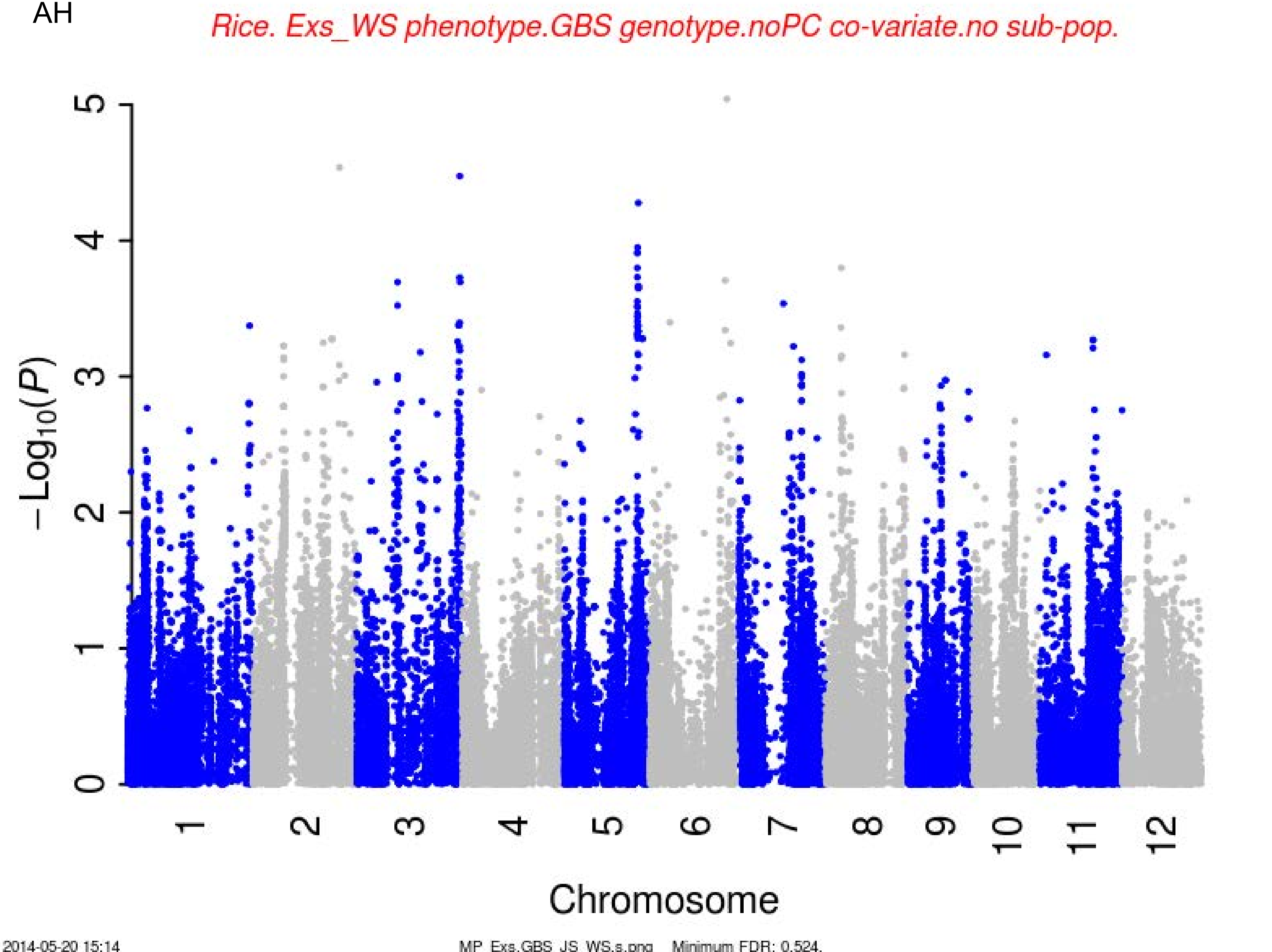

Rice. Exs_WS phenotype.GBS genotype.noPC co-variate.no sub-pop.
$-\mathrm{Log}_{10}(P)$
0
1
2
3
4
5
1
2
3
4
5
6
7
8
9
10
11
12
Chromosome
2014-05-20 15:14
MP Exs.GBS JS WS.s.png Minimum FDR: 0.524.

AI

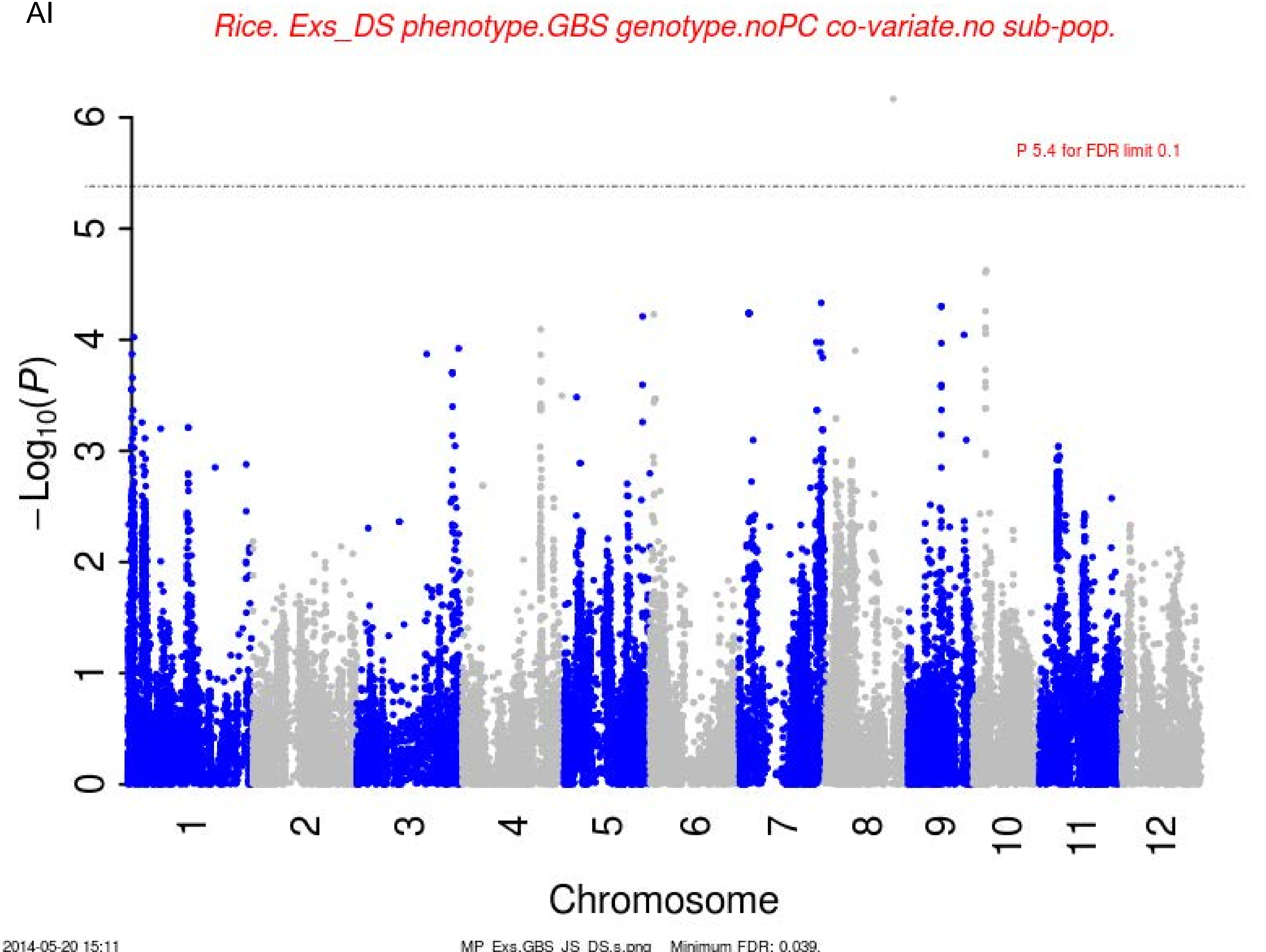
Rice. Exs_DS phenotype.GBS genotype.noPC co-variate.no sub-pop.
P 5.4 for FDR limit 0.1
$-\text{Log}_{10}(P)$
0
1
2
3
4
5
6
1
2
3
4
5
6
7
8
9
10
11
12
Chromosome
2014-05-20 15:11
MP_Exs.GBS_JS_DS.s.png Minimum FDR: 0.039.

AJ

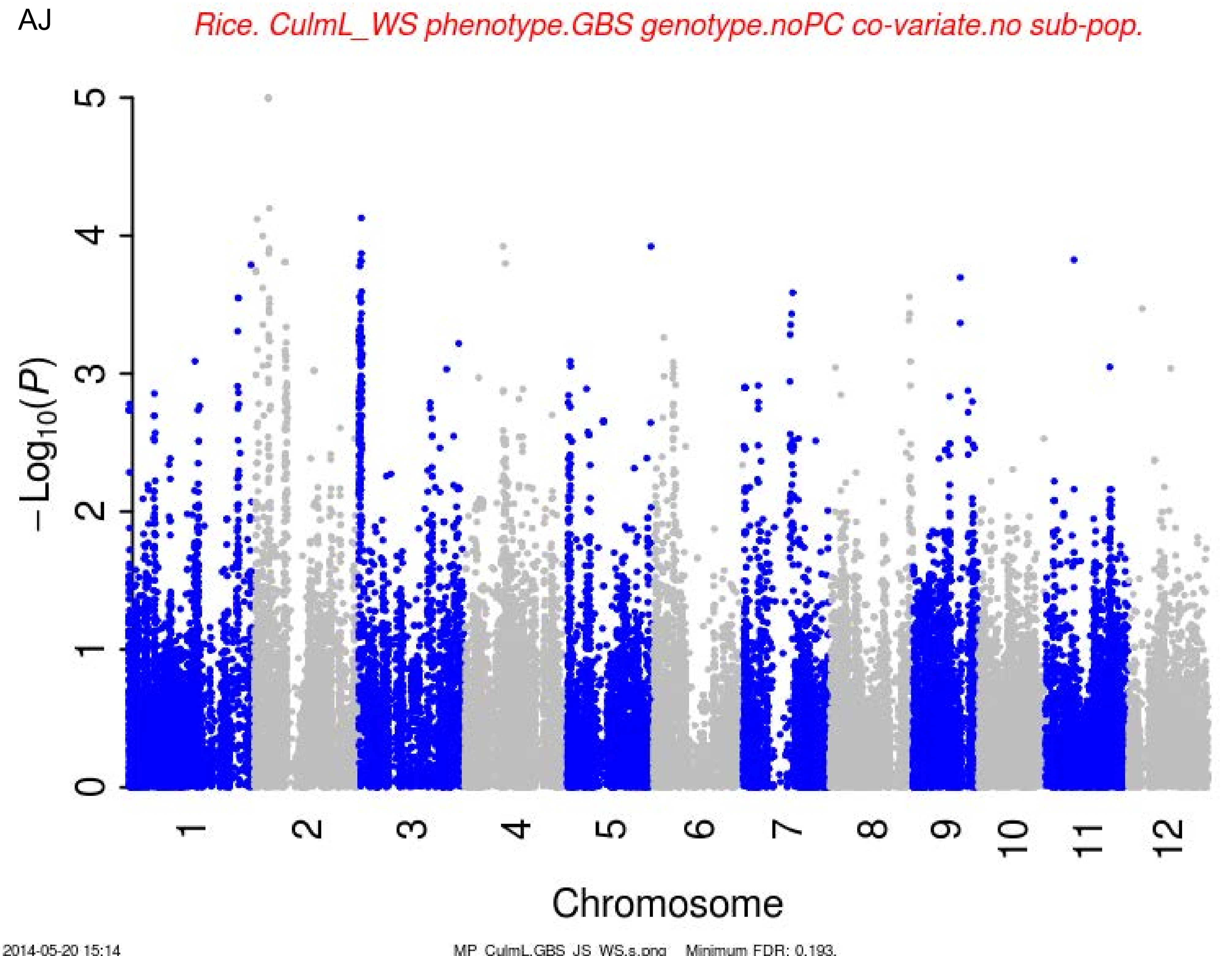
Rice. CulmL_WS phenotype.GBS genotype.noPC co-variate.no sub-pop.
5
4
3
2
1
0
$-\text{Log}_{10}(P)$
1
2
3
4
5
6
7
8
9
10
11
12
Chromosome
2014-05-20 15:14
MP_CulmL.GBS_JS_WS.s.png   Minimum FDR: 0.193.

AK

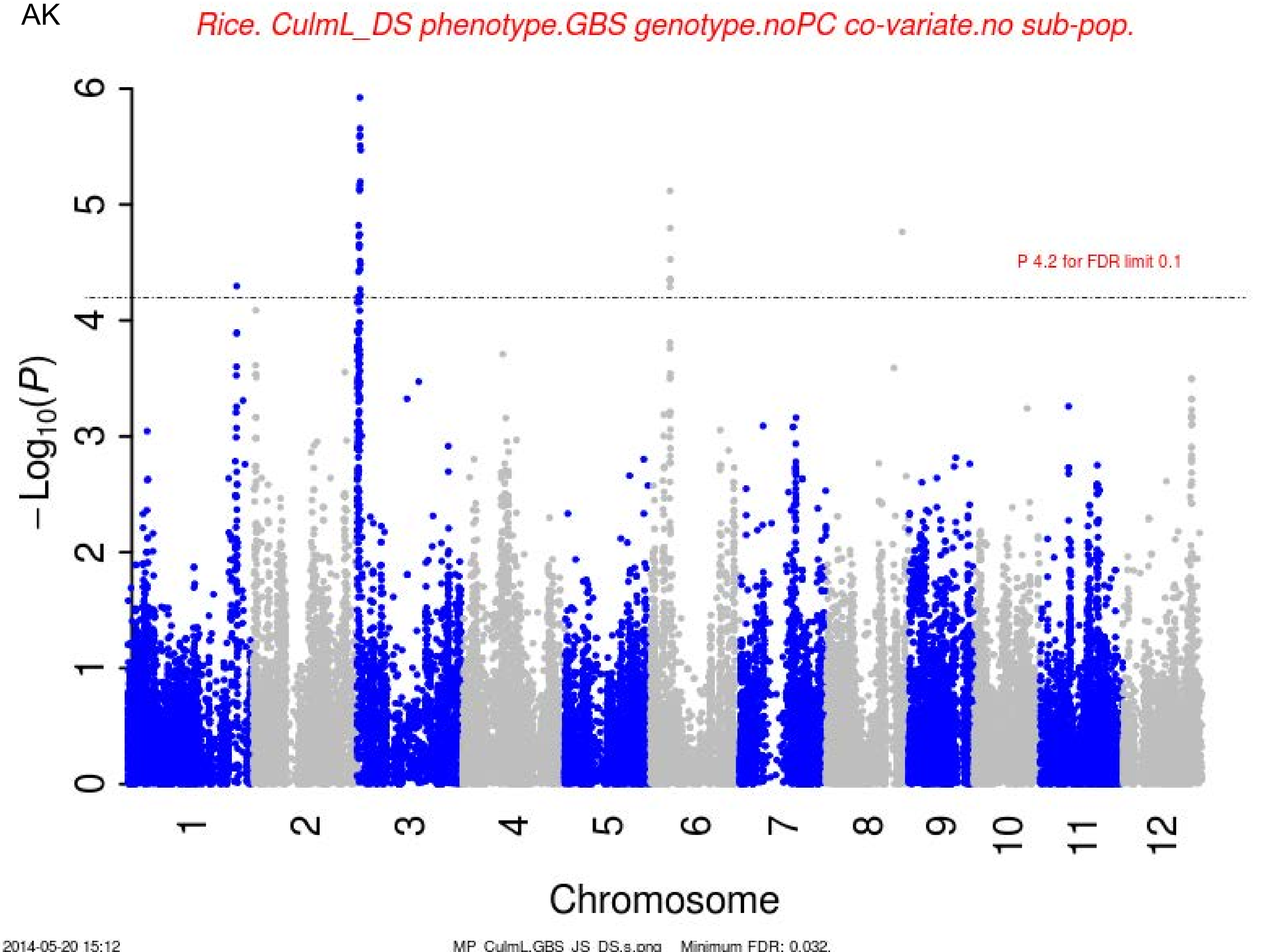

Rice. CulmL_DS phenotype.GBS genotype.noPC co-variate.no sub-pop.
P 4.2 for FDR limit 0.1
−Log10(P)
0
1
2
3
4
5
6
1
2
3
4
5
6
7
8
9
10
11
12
Chromosome
2014-05-20 15:12
MP_CulmL.GBS_JS_DS.s.png Minimum FDR: 0.032.

AL

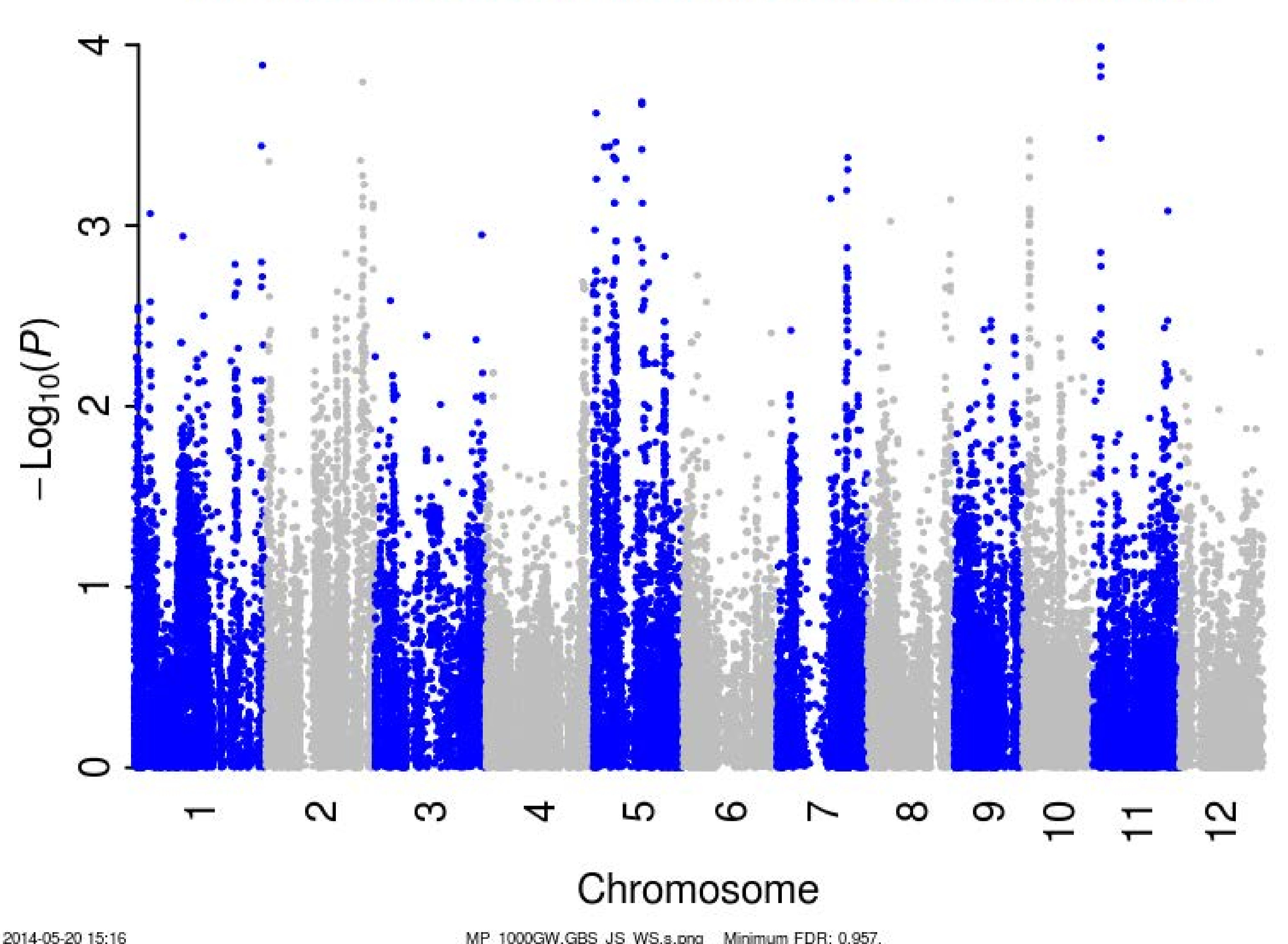
Rice. 1000GW_WS phenotype.GBS genotype.noPC co-variate.no sub-pop.
−Log10(P)
0
1
2
3
4
1
2
3
4
5
6
7
8
9
10
11
12
Chromosome
2014-05-20 15:16
MP_1000GW.GBS_JS_WS.s.png Minimum FDR: 0.957.
